# A Multistrategy Enhanced Starfish Optimization Algorithm for Wireless Sensor Network Deployment

**DOI:** 10.3390/biomimetics11100752

**Published:** 2026-10-18

**Authors:** Jianfu Luo, Mingyue Wang, Xu Ma, Hui Jie, Zhenyu Fan

**Affiliations:** 1 School of Engineering, Hulunbuir University, Hailar 021008, China; luojianfu@hlbec.edu.cn; 2 School of Business, Wuyi University, Wuyishan 354300, China; 3 School of Management Science and Engineering, Tianjin University of Finance and Economics, Tianjin 300222, China; maxu@tjufe.edu.cn; 4 China Aero Geophysical Survey and Remote Sensing Center for Natural Resources, Beijing 100083, China; fanzhenyu@mail.cgs.gov.cn

**Keywords:** starfish optimization algorithm, multistrategy enhancement, wireless sensor networks, deployment optimization

## Abstract

Wireless sensor network (WSN) deployment optimization is crucial for IoT sensing, requiring high coverage with limited nodes in complex search spaces. This paper proposes a multistrategy enhanced starfish optimization algorithm (MSESFOA) to address weaknesses of SFOA. MSESFOA introduces adaptive exploration regulation, a stage-wise combined mutation, and multiarm cooperative recombination, improving exploration flexibility, diversity preservation, and information fusion. Experiments on CEC 2017 and CEC 2022 show competitive accuracy, stability, and low deviation over 30 runs. In the tested 2D and 3D WSN cases, MSESFOA achieves lower mean uncovered ratios than SFOA, while the displayed deployment maps generally show fewer coverage gaps. The results support the competitiveness of MSESFOA for coverage optimization across the tested 2D and 3D scenarios with homogeneous sensors, a fixed sensor count, and a fixed sensing radius.

## 1. Introduction

Wireless sensor networks (WSNs), as parts of the fundamental sensing infrastructure of the Internet of Things, have been widely deployed in environmental monitoring, smart agriculture, industrial inspection, and emergency management. The deployment configuration of sensor nodes has a critical influence on coverage capability, monitoring reliability, and overall deployment and maintenance costs. As application scenarios extend from regular planar regions to complex terrains and spatial environments, developing high-quality and scalable deployment schemes under resource limitations remains a pressing challenge and requires efficient and robust intelligent optimization methods.

### 1.1. Research Background

For complex optimization problems with nonconvex and multimodal landscapes and potentially expensive objective evaluations, conventional deterministic approaches often struggle to achieve both solution quality and robustness within a reasonable computational budget. Consequently, swarm intelligence and metaheuristic algorithms, which are gradient-free and flexible in terms of implementation, have been widely adopted. The effectiveness of such methods largely depends on maintaining a proper balance between global exploration and local exploitation: insufficient exploration tends to cause premature convergence, whereas insufficient exploitation may prevent high-accuracy solutions from being obtained. As an emerging swarm optimizer, the starfish optimization algorithm (SFOA) [1] has attracted increasing interest because of its simple structure and intuitive mechanisms; however, in complex search spaces, it may still exhibit limitations such as inflexible exploration regulation, rapid diversity loss, and weak information fusion, which can decrease convergence efficiency and solution stability. Therefore, developing a multistrategy enhancement to strengthen the search process and improve robustness and efficiency should be highly prioritized.

WSN deployment optimization provides a representative engineering testbed for the above algorithmic studies. In cases with a certain monitored region and a limited number of sensors, the task is to determine sensor locations to achieve high coverage quality while satisfying practical resource and deployment constraints. Since coverage performance is strongly coupled with the geometric distribution of nodes and is typically evaluated via grid discretization or sampling-based approximation, the deployment problem is typically nonlinear and highly multimodal. When extended from 2D planar settings to 3D environments, the decision dimensionality and spatial structural complexity increase further, which substantially increases the search difficulty. Moreover, factors such as connectivity, energy/lifetime considerations, obstacle or no-go zones, and heterogeneous sensors can further complicate the formulation. Therefore, developing an optimizer that maintains stable performance across both 2D and 3D scenarios with strong cross-scenario applicability is of practical significance for enhancing the engineering usability of WSN deployments.

### 1.2. Literature Review

To clarify the research context and provide a basis for the proposed work, the related literature is reviewed in two parts. First, focus is placed on the applications and enhancements of the SFOA, summarizing representative improvement strategies and the remaining limitations. In the second part, the state-of-the-art methods in WSN deployment optimization, including coverage-oriented modeling objectives, common practical constraints, and mainstream intelligent optimization approaches, are reviewed, with particular attention to the challenges in complex scenarios and cross-scenario applicability. Building upon these insights, algorithmic enhancement is integrated with WSN deployment-oriented validation to establish a coherent research pipeline.

#### 1.2.1. Applications and Enhancements Based on the SFOA

The SFOA, proposed by Zhong [1] et al. in 2025, is a bioinspired swarm metaheuristic algorithm derived from the predation, exploration, and regeneration behaviors of starfishes. The SFOA features a concise structure, involves relatively few parameters, and has a strong search capability. Consequently, it has been rapidly adopted in various engineering and intelligent optimization tasks since its introduction. However, as the algorithm was proposed only recently, the number of studies on its enhancements and applications remains limited.

Recent improvements in the SFOA have focused on enhancing its exploration and exploitation balance, robustness, and adaptation to complex optimization tasks. Liu [2] proposed an enhanced variant SFOAL that integrates sine chaotic mapping, t-distribution mutation, and logarithmic spiral reverse learning, and this approach was successfully applied for ultrawideband indoor positioning. Similarly, Cao [3] developed a modified SFOA (named the MSFOA) using pheromone attraction and greedy dimension-by-dimension updates, which achieved excellent results in constrained engineering optimization tasks. These studies collectively indicated that hybrid and joint strategies can effectively mitigate premature convergence and dimensionality degradation issues. Chen et al. [4] proposed MCPSFOA, which combines SFOA and CPO updates with Lévy flight, Gaussian mutation, and stagnation-triggered reinitialization to enhance search performance. The method was evaluated on benchmark functions and engineering design problems.

In terms of application, the SFOA has also been embedded in domain-specific frameworks to address real-world energy, electronics and control problems. For example, Fathy [5] incorporated the SFOA into a microgrid energy management system with renewable energy uncertainty, achieving lower operating costs and improved convergence stability compared with other metaheuristics. Rajasekaran [6] proposed a hybrid SFOA-GAF-Net model for energy scheduling in photovoltaic-integrated smart grids, where hybrid energy storage and distribution parameters within a graph-attention network were optimized with SFO. With respect to the industrial IoT, Li [7] integrated the SFOA within a dynamic diffusion spatial–temporal graph convolutional framework to enhance anomaly detection and self-healing capability, demonstrating the adaptability of the algorithm to data-driven network optimization. Hua [8] employed the SFOA to tune the hyperparameters of a CNN-BiLSTM predictor, which was integrated with the K-means scenario and feature processing for short-term PV power forecasting. Experiments demonstrated lower RMSEs across scenarios and superior prediction accuracy compared with baseline methods. Pan [9] used the SFOA as a global optimizer coupled with a DNN–CNN surrogate to automate multiparameter design optimization of superjunction lateral double-diffused MOSFET (LDMOS) devices. The results showed that the framework reduces exploration cost while achieving a better breakdown voltage (BV) trade-off and a higher Baliga’s figure of merit (FOM).

Overall, existing studies indicated that the SFOA serves as a flexible optimizer that can be effectively combined with learning-based models, where it is frequently used for hyperparameter tuning, surrogate-assisted searches, or parameter calibration to enhance optimization quality in data-driven pipelines. However, the original SFOA may exhibit insufficient late-stage exploitation and local refinement abilities in some challenging scenarios, leading to slow convergence or suboptimal precision. These limitations motivate further multistrategy enhancements that strengthen exploitation while preserving exploration robustness, especially for complex engineering problems requiring stable performance across different scenarios.

#### 1.2.2. WSN Deployment Optimization: Models and Solution Methods

In existing research on WSNs, deployment optimization has moved beyond static 2D and single-objective formulations toward more realistic problem settings that account for 3D and terrain-aware environments; engineering constraints such as connectivity requirements, restricted or inaccessible areas, and deployment costs; and multiobjective trade-offs among coverage performance, energy consumption, network lifetime, and the number of sensors.

In parallel, solution approaches have progressed from standalone metaheuristics to multistrategy fusion schemes, geometry-guided optimization, and learning-assisted or reinforcement-learning-driven adaptive deployment. Overall, the literature can be viewed as converging around four closely related directions, covering two-dimensional static coverage enhancement and coverage-hole mitigation, three-dimensional and complex-terrain deployment, constraint-aware multiobjective modeling that emphasizes energy efficiency and cost, and hybrid intelligent frameworks that integrate geometric modeling with data-driven decision-making.

A large body of work has focused on improving 2D deployment quality with typical sensing models and enhancing grid/sampling-based coverage evaluation. In this context, metaheuristics are often enhanced by multistrategy fusion to alleviate premature convergence and obtain more uniform node distributions. For example, Reddy [10] combined opposition-based learning, sequential dimensionwise updates, and PSO-assisted random walks, achieving high coverage and efficiency across different network sizes. Wu [11] enhanced the whale optimization algorithm by introducing inertia weighting and dynamic parameter adaptation, which stabilized convergence and improved deployment performance under varying node densities. Similarly, Liu [12] developed multistrategy fusion GWO designs by integrating diversified initialization, elitist memory, leader rotation, and hybrid mutation operators, reporting higher coverage and more consistent convergence behavior than achieved with other methods. Beyond pointwise deployment quality, Muhammad [13] focused on continuous coverage and coverage-hole governance by combining velocity-scaled adaptive PSO with quasiuniform Hammersley initialization and Delaunay-triangulation-based hole detection, which accelerated convergence and reduced the number of uncovered regions. Wang and Li [14] proposed a modified marine predator algorithm for coverage optimization in a forest fire monitoring setting by integrating tent-mapping initialization, a hybrid search strategy, golden-sine guidance, and a stage-adjustment mechanism, yielding higher average coverage and a more uniform node distribution. Overall, these works collectively suggest that multistrategy enhancement, integrating improved initialization, diversity preservation, and adaptive exploitation, has become a prevailing paradigm for 2D coverage optimization.

When extending from planar regions to 3D spaces or real terrains, the decision dimensionality and spatial complexity increase substantially, which often exacerbates convergence to local optima and performance degradation. To address these issues, some works incorporate dedicated mechanisms for 3D search stabilization and late-stage refinement. Meng [15] proposed an augmented secretary bird optimization algorithm for WSN deployment, aiming to improve convergence behavior and robustness. Similarly, Wang [16] developed a 3D coverage optimization strategy based on an adaptive-inertia-weighted Arctic Puffin optimization algorithm and reported improved coverage performance with stable convergence. In addition, Yao [17] presented a distributed optimization strategy that combines tangent-search exploration with quadratic-interpolation refinement, providing another viable option for scalable deployment optimization.

Building on these 3D and terrain-oriented studies, another line of research has shifted from coverage-only objectives toward constraint-aware and resource-aware deployment under practical operational requirements. From the energy perspective, Ren [18] formulated energy-efficient coverage enhancement and developed a competitive-learning optimizer together with a multiobjective extension to balance coverage quality and energy consumption in both 2D and 3D deployments. In directional sensor networks, Tran [19] addressed connectivity-constrained coverage maximization by refining both sensor locations and orientations within an improved artificial bee colony framework, enabling effective solutions for area, target, and barrier coverage while maintaining reliable connectivity. Cost and node minimization have also been emphasized; Tong [20] proposed an enhanced hybrid PSO with spatial position encoding to improve coverage while dynamically reducing the number of deployed sensors and explicitly accounting for restricted and noncritical regions. Beyond these constraint settings, deployment evaluation is increasingly linked to task-level monitoring effectiveness, and Doodman [21] introduced a context-aware Voronoi-based strategy for point-sensor placement in spatiotemporal field monitoring by incorporating spatial context and temporal variability. Geometric structuring can further be coupled with swarm intelligence algorithms, as Perumal [22] combined Delaunay triangulation with glowworm swarm optimization in a hybrid framework to jointly improve coverage and energy-related performance. For dynamic deployment, Zhou [23] developed a deep reinforcement learning approach that leverages energy-aware sensing models, hierarchical state representation, and cooperative multiobjective rewards to support adaptive coverage under changing conditions. Finally, system-level optimization beyond deployment has also been explored, and Ma [24] investigated IoT-WSN task allocation and proposed an opposition-based learning and elite-cloning enhanced grasshopper strategy to improve overall network gain. Collectively, these studies reinforce the shift from purely geometric coverage maximization toward integrated optimization that jointly considers connectivity, energy, cost, task effectiveness, and learning-driven adaptability.

Despite the above progress, several challenges remain in WSN deployment optimization. Many metaheuristic-based approaches still show limited late-stage exploitation and weak local refinement when the search space becomes complex or high-dimensional, which can slow convergence and reduce final placement precision. In addition, the performance advantage observed in simplified planar settings does not always transfer to 3D or terrain-aware environments, especially when connectivity, energy, cost, or restricted-region constraints are introduced, underscoring the need for stronger cross-scenario applicability and more stable behavior across heterogeneous conditions. Moreover, geometry-guided or learning-assisted hybrid frameworks can enhance practicality; however, they are often built upon problem-specific components and may not provide a lightweight, generally applicable enhancement mechanism that consistently improves robustness and exploitation. These observations motivate the development of multistrategy-enhanced optimizers that maintain stable performance across both 2D and 3D WSN deployment scenarios, which is the focus of this work.

### 1.3. The Main Contents of This Paper

The present study focuses on coordinating search regulation, population perturbation, and information exchange within the SFOA framework. To this end, MSESFOA integrates three complementary mechanisms. An adaptive exploration–exploitation switching mechanism regulates the selection of the basic search branches as the optimization proceeds. A stage-based combined mutation operator employs differential mutation, single-coordinate perturbation, and Gaussian mutation at different search stages, with activation probabilities determined by individual fitness. A multi-arm cooperative recombination operator combines neighbor cooperative perturbation with intradimensional recombination to facilitate information exchange between individuals and across coordinates.

This design differs from MCPSFOA, which combines SFOA and CPO position updates. MSESFOA retains the SFOA search framework and emphasizes the coordination of adaptive branch selection, stage-dependent mutation, and cooperative recombination. Although Gaussian mutation is used in both methods, MSESFOA places it in the late search stage as part of a combined mutation scheme whose activation depends on individual fitness.

The evaluation covers numerical optimization and WSN coverage deployment. The CEC2017 and CEC2022 benchmark suites are used to examine solution quality, variability across independent runs, and convergence behavior. A unified grid-based coverage formulation is also adopted for eight 2D and four 3D WSN deployment cases, with the uncovered ratio serving as the objective function. These experiments assess the proposed method across different function landscapes and deployment configurations.

### 1.4. Highlghts of This Paper

In this paper, 2D and 3D WSN coverage deployment optimization is targeted by the MSESFOA, and a unified evidence chain spanning benchmark suites and engineering deployment cases is established to substantiate the effectiveness, stability, and cross-scenario applicability of the proposed approach.

MSESFOA integrates adaptive exploration regulation, stage-based combined mutation, and multi-arm cooperative recombination to regulate search strategy selection, introduce stage-dependent perturbations, and enrich information exchange.

For CEC 2017 and CEC 2022, the MSESFOA is evaluated against 15 algorithms with 30 independent runs, achieving the best overall performance, with average ranks of 1.2759 and 1.50, respectively. Moreover, Wilcoxon signed-rank tests with Holm correction indicate statistically significant differences in favor of MSESFOA against all 15 competing algorithms on CEC2017 and 14 of the 15 competing algorithms on CEC2022 at a family-wise significance level of 0.05.

To evaluate MSESFOA on a coverage-oriented deployment task, a unified grid-based 2D/3D WSN deployment model is formulated by using the uncovered ratio as the fitness indicator, and maximizing coverage is equivalently expressed as minimizing the uncovered ratio; extensive comparisons are conducted on eight 2D deployment cases and four 3D deployment cases via uncovered-ratio statistics, convergence curves, and spatial coverage maps. The results show that the final rank is the highest in both the 2D and 3D cases, whereas the coverage maps indicate more uniform deployments with less redundancy and overlap and fewer uncovered cavities than those in other maps.

## 2. The Process of the Starfish Optimization Algorithm

The SFOA is inspired by the unique biological behaviors of starfish, namely, exploration, predation, and regeneration. Equipped with multiple arms, each with sensory abilities at the tip, starfish can actively investigate complex marine environments. In predation, the coordinated movement of the arms allows prey to be surrounded, after which the stomach is everted for external digestion. Under threat, starfish can utilize arm autotomy to escape and subsequently regenerate lost tissues to restore integrity. These behavioral traits naturally correspond to wide-range exploration, local exploitation, and the ability to escape local optima, providing a biologically motivated foundation for algorithm design. Accordingly, SFOA comprises two complementary phases: exploration for investigating the search space and exploitation for refining candidate solutions.

### 2.1. Initialization

In the population initialization phase of the SFOA, the positions of the starfish are randomly generated within the variable search space, which can be represented by the following position matrix:

(1) $$X={\left[\begin{array}{cccc} X_{11} & X_{12} & \ldots & X_{1D}\\ X_{21} & X_{22} & \ldots & X_{2D}\\ \vdots & \vdots & \ddots & \vdots \\ X_{N1} & X_{N2} & \ldots & X_{ND} \end{array}\right]}_{N\times D}.$$

Here, X denotes the positions of all the individuals in the starfish population. In this matrix, N represents the population size, and *D* denotes the dimensionality of the optimization problem.

Any element $X_{ij}$ in Formula (1), which represents the value of the *i*-th individual in the *j*-th dimension, is initialized as follows:

(2) $$\begin{array}{l} X_{ij}=lb_{j}+rand\cdot \left(ub_{j}-lb_{j}\right),\\ i=1,2,\ldots ,N,\;\;\;j=1,2,\ldots ,D, \end{array}$$

where $lb_{j}$ denotes the lower bound of the search space in the *j*-th dimension and $ub_{j}$ denotes the upper bound. The term rand represents a random number uniformly distributed in the range of [0, 1].

By substituting each solution vector in the matrix, that is, the *N* row vectors of dimension *D*, into the objective function, a vector of fitness values for all starfish individuals is obtained as follows:

(3) $$F={\left[f\left(X_{1}\right),f\left(X_{2}\right),\ldots ,f\left(X_{N}\right)\right]}_{N\times 1}^{T},$$

where F denotes the vector of the fitness values and $f\left(X_{i}\right)$ represents the fitness function value of the *i*-th starfish individual.

### 2.2. Exploration Phase

In the exploration phase, the SFOA simulates the behavior of starfish arms when they are searching the surrounding environment. In this phase, the algorithm adopts different search strategies depending on the dimensionality of the problem.

When the search dimension *D* > 5, a five-dimensional search mode is employed. Specifically, five dimensions $j\in \left\{1,2,\ldots ,D\right\}$ are randomly selected for exploration, and the selected coordinates of each individual are updated as follows:

(4) $$Y_{i,j}^{T}=\left\{\begin{array}{l} X_{i,j}^{T}+\alpha_{1}\cdot \left(X_{best,j}^{T}-X_{i,j}^{T}\right)\cdot \cos\theta ,\;\;\;\;rand\leq 0.5\\ X_{i,j}^{T}-\alpha_{1}\cdot \left(X_{best,j}^{T}-X_{i,j}^{T}\right)\cdot \sin\theta ,\;\;\;\;\;rand>0.5 \end{array}\right.,$$

where $Y_{i,j}^{T}$ represents the updated position of the *i*-th individual in the *j*-th dimension. $X_{i,j}^{T}$ denotes the current position of the *i*-th individual in the *j*-th dimension. $X_{best,j}^{T}$ is the position of the globally best individual in the *j*-th dimension at the current iteration.

The parameters $\alpha_{1}$ and θ are calculated as follows:

(5) $$\alpha_{1}=\left(2\cdot rand-1\right)\cdot \pi ,$$

(6) $$\theta =\frac{\pi }{2}\cdot \frac{T}{T_{\max}},$$

where T denotes the current iteration number and $T_{\max}$ is the maximum number of iterations.

When D≤5, a single-dimensional search mode is employed, and the independent search of a single arm in a specific dimension is simulated. The selected coordinate is updated as follows:

(7) $$Y_{i,j}^{T}=E_{t}\cdot X_{i,j}^{T}+A_{1}\cdot \left(X_{k1,j}^{T}-X_{i,j}^{T}\right)+A_{2}\cdot \left(X_{k2,j}^{T}-X_{i,j}^{T}\right),$$

where $X_{k1,j}^{T}$ and $X_{k2,j}^{T}$ denote the *j*-th dimensional positions of two individuals randomly selected from the population, while $A_{1}$ and $A_{2}$ are random numbers within the interval $\left(-1,1\right)$. The term $E_{t}$ represents the current energy level of the starfish, which is calculated with Formula (8).

(8) $$E_{t}=\frac{T_{\max}-T}{T_{\max}}\cdot \cos\theta .$$

This mechanism mimics the exploratory behavior of a single starfish arm when it conducts an independent search within a specific dimension, thereby enhancing the algorithm’s ability to maintain diversity in low-dimensional spaces.

### 2.3. Exploitation Phase

The exploitation phase of the SFOA combines the predation and regeneration behaviors of starfish. Specifically, the predation behavior is simulated to strengthen the algorithm’s local search capability, and the regeneration behavior is modeled to enhance its global escape ability. These two mechanisms provide position updates for local search and regeneration, respectively.

#### 2.3.1. Predation Behavior

Predation behavior is modeled using a bidirectional parallel search strategy. First, the distance between the current global best solution and five randomly selected individuals is calculated as follows:

(9) $$d_{m}=X_{best}^{T}-X_{mj}^{T}\;\;\;\;\;\;\;\;\;\;\;\;\;\;\;\;\;\;\;m=1,\ldots ,5,$$

where $d_{m}$ represents the distance between the global best individual at iteration T and the five randomly selected starfish and *mj* denotes the index of the selected individuals.

Then, two distance vectors $d_{m1}$ and $d_{m2}$ are chosen randomly, and the position of each starfish is updated to simulate the predation behavior, as follows:

(10) $$Y_{i}^{T}=X_{i}^{T}+rand_{1}\cdot d_{m1}+rand_{2}\cdot d_{m2},$$

where $rand_{1}$ and $rand_{2}$ are random numbers uniformly distributed within $\left[0,1\right]$.

#### 2.3.2. Regeneration Behavior

During the predation process, starfish move slowly, which makes them vulnerable to attacks from predators. When attacked, a starfish may autotomize one of its arms and later regenerate it to escape danger. Therefore, in the SFOA, the regeneration phase is applied only to the last individual of the population, namely, *i* = *N*, at each iteration. Since the regeneration process in nature requires several months of growth, the movement speed of starfish during this phase is modeled to be extremely slow. Accordingly, the regeneration behavior in the SFOA is simulated by updating the position via Formula (11).

(11) $$Y_{i}^{T}=\exp\left(-\frac{T\cdot N}{T_{\max}}\right)\cdot X_{i}^{T}.$$

## 3. Proposed Algorithm

The original SFOA provides a concise search framework, but several aspects of its mechanisms motivate further enhancement. Its fixed-probability strategy selection offers limited flexibility in regulating the search as optimization progresses. Moreover, its basic updates do not include a dedicated mutation scheme that changes the perturbation form across search stages. Although information from other individuals is used in the original updates, neighbor cooperative perturbation and intradimensional recombination are not explicitly combined. These aspects motivate the development of complementary mechanisms for search regulation, population perturbation, and information exchange.

Accordingly, MSESFOA introduces three improvement strategies with distinct roles. The adaptive exploration strategy regulatory mechanism adjusts the probability parameter to provide more flexible search regulation. The stage-based combined mutation operator introduces different perturbation forms in the early, middle, and late search stages, with fitness-dependent activation probabilities. The multi-arm cooperative recombination operator combines neighbor cooperative perturbation with intradimensional recombination to enrich information exchange. The proposed contribution is the coordinated integration of these mechanisms within the SFOA framework, with their effectiveness evaluated through benchmark tests and WSN coverage deployment experiments.

### 3.1. Adaptive Exploration Strategy Regulatory Mechanism

In the exploration phase of the SFOA, the update strategy is relatively simple, and its adaptability to the number of dimensions still requires further improvement. When the search dimension is high, the use of a fixed-probability search strategy cannot fully exploit the global search ability of the algorithm, making it prone to converging to local optima.

To address this issue, an adaptive strategy regulatory mechanism is proposed in this section. By adjusting the value of the probability parameter *GP*, the usage frequency of different exploration strategies in Formula (4) can be controlled, thereby enabling the algorithm to achieve better regulation of the search direction at different stages of iteration. Accordingly, the position update in Formula (4) is modified as follows, with the adaptive parameter GP defined below:

(12) $$Y_{i,j}^{T}=\left\{\begin{array}{l} X_{i,j}^{T}+\alpha_{1}\cdot \left(X_{best,j}^{T}-X_{i,j}^{T}\right)\cdot \cos\theta ,\;\;\;\;rand\leq GP\\ X_{i,j}^{T}-\alpha_{1}\cdot \left(X_{best,j}^{T}-X_{i,j}^{T}\right)\cdot \sin\theta ,\;\;\;\;\;rand>GP \end{array}\right.,$$

(13) $$GP=\left\{\begin{array}{l} 0.5\cdot \exp\left(-\frac{3\cdot T}{T_{\max}}\right)+0.2\cdot rand,\;\;\;\;\;\;\;\;\;\;\;\;\;\;\;\;\;rand<0.5\\ \frac{5+5\cdot \cos\left(\frac{T\cdot \pi }{T_{\max}}\right)+\left(rand-0.5\right)}{10},\;\;\;\;\;rand\geq 0.5 \end{array}\right..$$

### 3.2. Design of the Stage-Based Combined Mutation Operator

In the position updating process of the SFOA, the algorithm relies mainly on the calculation of differential directions within the population and lacks auxiliary mechanisms for the structural perturbation of individuals. As the iterations progress, the population tends to suffer from diversity degradation and premature convergence, which adversely affect the global search capability and convergence accuracy of the algorithm.

To improve the adaptability of the algorithm at different stages, increase population diversity, and increase individual jump ability, a stage-based mutation operator is introduced. The operator divides the optimization process into three stages according to the iteration progress and designs corresponding perturbation strategies for each stage, thereby achieving a smooth transition from wide-range exploration to fine-scale local searches.

First, the fitness values of all individuals are normalized, and on the basis of their relative performance within the current population, the mutation probability is dynamically calculated. The normalized objective-function value is calculated as follows:

(14) $$normFitness_{i}^{T}=\frac{f\left(X_{i}^{T}\right)-f_{\min}^{T}}{f_{\max}^{T}-f_{\min}^{T}+\varepsilon },$$

where $normFitness_{i}^{T}$ denotes the normalized objective-function value of the i-th starfish individual at iteration *T*; $f\left(X_{i}^{T}\right)$ is its numerical objective-function value; and $f_{\min}^{T}$ and $f_{\max}^{T}$ denote the minimum and maximum objective-function values in the current population, respectively. Since all the problems considered in this study are minimization problems, a smaller value of $f\left(X_{i}^{T}\right)$ represents a higher-quality solution. A small positive constant ε is included in the denominator and is set to the MATLAB machine epsilon for double-precision arithmetic, namely, $2.2204\times 10^{-16}$. When $f_{\max}^{T}-f_{\min}^{T}$ is substantially larger than ε, the influence of ε on the normalization result is negligible. Its primary purpose is to prevent division by zero when all individuals have identical objective-function values.

On the basis of Formula (14), the mutation probability of each individual can subsequently be calculated as shown in Formula (15).

(15) $$P_{i}^{T}=0.1+0.3\cdot \left(1-normFitness_{i}^{T}\right).$$

According to Formulas (14) and (15), an individual with a smaller objective-function value has a normalized value closer to 0 and is therefore assigned a higher mutation probability. Conversely, an individual with a larger objective-function value has a normalized value closer to 1 and is assigned a lower mutation probability. Accordingly, the mutation probability varies approximately within the interval [0.1, 0.4]. This design deliberately increases the perturbation frequency of promising individuals, thereby strengthening the search around high-quality regions and providing additional opportunities for these individuals to escape local optima. After the mutation operation, greedy selection is performed: a mutated candidate replaces the corresponding original individual only if it produces a smaller objective-function value. Therefore, deteriorated mutated candidates are rejected, and previously obtained high-quality solutions are preserved. If all individuals have identical objective-function values, their normalized values are all equal to 0 and their mutation probabilities are all equal to 0.4, which increases population perturbation when no objective-value diversity is present.

For example, suppose that $f_{\min}^{T}=10$, $f_{\max}^{T}=30$, and $\varepsilon =2.2204\times 10^{-16}$. For three individuals with objective-function values of 10, 20, and 30, the normalized values calculated using Formula (14) are 0, approximately 0.5, and approximately 1, respectively. The corresponding mutation probabilities calculated using Formula (15) are 0.4, approximately 0.25, and approximately 0.1, respectively. Hence, for the minimization problems considered in this study, relatively better individuals are assigned higher mutation probabilities, whereas the subsequent greedy selection mechanism prevents the loss of their original high-quality solutions.

After determining the mutation probability of each individual, to meet the requirements of different exploration phases, the operator is divided into three stages according to the number of iterations.

#### 3.2.1. Early Search Stage

When the current iteration number *T* is less than 30% of the maximum iteration number, to enhance the jumping ability of the search, three individuals $X_{r1}^{T}$, $X_{r2}^{T}$, and $X_{r3}^{T}$ are randomly selected from the population to construct the mutation form, as shown in Formula (16):

(16) $$Y_{i}^{T}=X_{r1}^{T}+F\cdot \left(X_{r2}^{T}-X_{r3}^{T}\right),$$

where F is a dynamic scaling factor whose value is calculated with Formula (17).

(17) F=0.5+0.3·rand.

This expression defines the default scaling-factor setting used in MSESFOA. Its influence is examined in the parameter sensitivity analysis in Section 4.3.2.

#### 3.2.2. Middle Search Stage

When the current iteration number is at least 30% but less than 70% of the maximum number of iterations, to increase the local structural diversity of individuals, a single-dimensional perturbation mechanism is introduced. The coordinate perturbation is defined as follows:

(18) $$Y_{i,j}^{T}=X_{i,j}^{T}+\gamma \cdot \frac{ub_{j}-lb_{j}}{10},$$

where γ is a random number within the interval $\left[-1,1\right]$.

#### 3.2.3. Late Search Stage

When the number of iterations reaches 70% of the maximum number of iterations, Gaussian mutation is applied to individuals to increase the local search capability. The Gaussian mutation update is defined as follows:

(19) $$Y_{i,j}^{T}=X_{i,j}^{T}+\frac{ub_{j}-lb_{j}}{20}\cdot Gauss\left(0,1\right),$$

where $Gauss\left(0,1\right)$ denotes a random variable following a Gaussian distribution with mean 0 and variance 1.

### 3.3. Multi-Arm Cooperative Recombination Operator

Since the SFOA lacks an effective information fusion mechanism among individuals, the cooperative search ability of the population is relatively weak, and the propagation of high-quality solutions is relatively slow. In complex and high-dimensional search spaces, individuals are prone to falling to local optima, making it difficult to achieve efficient exploitation.

To address this problem, inspired by the biological characteristics of “multi-arm coordinated movement” in starfish, a multi-arm cooperative recombination operator is proposed. The operator incorporates two levels, including cooperative perturbation between pairs of individuals and self-reconstruction of individuals. This operator simulates the cooperative feeding and fusion behavior of multiple arms in space, thereby enhancing the convergence speed of the population as well as its local exploitation capability.

#### 3.3.1. Neighbor Cooperative Perturbation

Each individual selects a neighboring individual according to its relative position in the population, and a perturbation-based search and fusion operation is performed. For the *i*-th starfish individual at iteration *T*, the neighboring individual $X_{k}^{T}$ is defined as shown in Formula (20).

(20) $$X_{k}^{T}=\left\{\begin{array}{l} X_{i+1}^{T}\;\;,\;\;\;\;\;i\;\;\;\;\;\;\;\mathrm{is}\;\;\;\;\mathrm{an}\;\;\;\;\mathrm{odd}\;\;\;\mathrm{number}\\ X_{i-1}^{T}\;\;,\;\;\;\;\;i\;\;\;\;\;\;\;\;\mathrm{is}\;\;\;\;\mathrm{an}\;\;\;\;\mathrm{even}\;\;\;\mathrm{number} \end{array}\right..$$

For the *j*-th dimension of the *i*-th starfish individual, the perturbation process is defined as shown in Formula (21).

(21) $$Y_{i,j}^{T}=\left\{\begin{array}{l} rand\cdot X_{i,j}^{T}+\left(1-rand\right)\cdot X_{k,j}^{T}+\gamma \cdot \left(X_{i,j}^{T}-X_{k,j}^{T}\right)\\ rand\cdot X_{k,j}^{T}+\left(1-rand\right)\cdot X_{i,j}^{T}+\gamma \cdot \left(X_{k,j}^{T}-X_{i,j}^{T}\right) \end{array}\right..$$

According to Formula (21), when *i* is an odd number, the first branch is executed, meaning that the individual performs cooperative perturbation with its subsequent neighbor. When *i* is an even number, the second branch is executed, meaning that the individual performs cooperative perturbation with its preceding neighbor.

#### 3.3.2. Intradimensional Recombination

To enhance the local search capability of the algorithm, each individual performs a fusion and recombination operation within its own dimensions. Specifically, for the *j*-th dimension of the *i*-th starfish individual, $X_{i,j}^{T}$ is generated by fusing two other randomly selected dimensions *j*1 and *j*2 from the same individual. The updating process is expressed in Formula (22).

(22) $$Y_{i,j}^{T}=rand\cdot X_{i}^{j1}+\left(1-rand\right)\cdot X_{i}^{j2}.$$

By simulating the cooperative predation behavior of multiple starfish arms in space, this operator integrates structural perturbation between individuals and dimensional recombination within individuals, thereby effectively improving both the information exchange efficiency and the local search capability of the population. The neighbor-based perturbation mechanism among individuals enhances the diversity of search space exploration, whereas the intradimensional recombination mechanism strengthens the local exploitation ability. The synergistic effect of these two structures helps avoid premature convergence and accelerates the aggregation of high-quality solutions, thus significantly improving the global optimization performance and convergence stability of the algorithm.

### 3.4. Boundary-Handling Strategies

During the exploration phase of the basic SFOA update, if an updated coordinate $Y_{i,j}^{T}$ falls outside the interval $\left[lb_{j},ub_{j}\right]$, it is restored to its value $X_{ij}^{T}$ before the current update. Componentwise clipping is then performed as a final boundary check. During the exploitation phase, the generated position is directly clipped using Formula (23).

(23) $$Y_{i,j}^{T}\leftarrow \min\left\{ub_{j},\max\left\{lb_{j},Y_{i,j}^{T}\right\}\right\}.$$

For the stage-based combined mutation operator, a one-step reflection strategy is used to bring out-of-bound coordinates back into the feasible interval, as shown in Formula (24).

(24) $$Y_{i,j}^{T}=\left\{\begin{array}{l} 2\cdot ub_{j}-Y_{i,j}^{T},\;\;\;\;\;\;\;\;\;\;\;\;\;\;\;\;\;\;\;\;Y_{i,j}^{T}>ub_{j}\;\\ 2\cdot lb_{j}-Y_{i,j}^{T},\;\;\;\;\;\;\;\;\;\;\;\;\;\;\;\;\;\;\;\;\;\;Y_{i,j}^{T}<lb_{j}\\ Y_{i,j}^{T},\;\;\;\;\;\;\;\;\;\;\;\;\;\;\;\;\;\;\;\;\;\;\;\;\;\;\;\;\;\;\;\;\;\;\;\;\;\;\;\;\;\;\;\;\;\;\;\;\;\;\;\;lb_{j}\leq Y_{i,j}^{T}\leq ub_{j}\;\; \end{array}\right..$$

The reflection operation maps the excess displacement across the violated boundary, retaining information on the attempted mutation magnitude rather than setting the coordinate directly to the boundary. Both the neighbor cooperative perturbation and the intradimensional recombination operations apply the componentwise clipping rule given above after generating each candidate position.

### 3.5. Pseudocode of the MSESFOA

Within each iteration, *GP* is first calculated using Formula (13), and either exploration or exploitation is selected by comparing a random number with *GP*. The selected operation is followed by boundary handling, objective-function evaluation, and greedy selection. The stage-based combined mutation operator is then applied, followed by reflection-based boundary handling, evaluation of all returned candidates, and greedy selection. Next, neighbor cooperative perturbation and intradimensional recombination are performed sequentially, each followed by clipping, evaluation, and greedy selection. Finally, the main routine reevaluates the population returned by the recombination operator and updates the global best solution. Thus, objective-function evaluations are conducted at intermediate stages rather than only after the complete update cycle. The corresponding procedure is summarized in Algorithm 1 (in page 13).

### 3.6. Complexity Analysis of the MSESFOA

Let *N*, *D*, and $T_{\max}$ denote the population size, problem dimensionality, and maximum number of iterations, respectively. Table 1 summarizes the module-level computational complexity of SFOA and MSESFOA.

Here, $M_{T}$ is the number of individuals undergoing mutation at iteration T, and $\bar{\rho }$ is the average mutation activation proportion over the complete run.

(25) $$\bar{\rho }=\frac{\sum_{T=1}^{T_{\max}}M_{T}}{N\cdot T_{\max}}.$$

In summary, while keeping the computational complexity within approximately 2–3 times that of the SFOA, the MSESFOA achieves stronger global search capability and enhanced local exploitation performance, demonstrating favorable cost-effectiveness.
**Algorithm 1:** Pseudocode of the MSESFOA**Input:** Starfish population size *N*, maximum number of iterations *T*_max_, dimensionality *D*, boundary range [*lb*, *ub*], and objective function *fobj* **Output:** The best fitness value *fval_best* and the corresponding position *xpos_best***1**Initialize the starfish population according to the procedure in Section 2.1.**2**Record the position *xpos_best* of the global best individual and its fitness value *fval_best*.**3****For** *T* = 1 to *T*_max_ **do****4**Calculate GP according to Formula (13).**5****If** rand < GP **then****6** **If** D≤5 then**7** Generate exploration candidates according to Formula (7).**8** **Else****9** Generate exploration candidates according to Formula (12).**10** **End if****11**Apply restoration followed by clipping (Section 3.4).**12****Else****13** Generate exploitation candidates according to Section 2.3.**14** Apply clipping (Section 3.4).**15****End if****16**Evaluate candidates, apply greedy selection, and update positions and fitness values.**17**Generate mutation candidates according to Section 3.2.**18**Apply one-step reflection (Section 3.4), and evaluate the returned mutation candidates and apply greedy selection.**19**Execute the multi-arm cooperative recombination operator (Section 3.3), and update positions and fitness values.**20**Reevaluate the returned population, apply greedy selection, and update the global best.**21**Record the global best objective-function value.**22****End for**

## 4. Numerical Experiments

To evaluate the optimization performance of MSESFOA across different problem landscapes, numerical experiments are conducted using the CEC2017 and CEC2022 benchmark suites. The evaluation considers solution quality, variability across independent runs, and convergence behavior. In addition to comparisons with other algorithms, ablation and parameter sensitivity experiments are conducted on both suites to examine the contributions of the three enhancement mechanisms and the effects of selected parameter settings, as presented in Section 4.3.

MSESFOA is compared with the original SFOA and 14 other algorithms: seahorse optimizer (SHO) [25], flood algorithm (FLA) [26], triangulation topology aggregation optimizer (TTAO) [27], crayfish optimization algorithm (COA) [28], black-winged kite algorithm (BKA) [29], ivy algorithm (IVY) [30], Newton–Raphson-based optimizer (NRBO) [31], pied kingfisher optimizer (PKO) [32], enzyme action optimizer (EAO) [33], artificial lemming algorithm (ALA) [34], rime-ice optimizer (RIME) [35], improved rime-ice optimizer (IRIME) [36], snow ablation optimizer (SAO) [37], and improved snow ablation optimizer (ISAO) [38]. Algorithm-specific parameters follow the settings reported in their respective publications, while the population size and maximum number of iterations are kept identical within each comparison. Each benchmark function is evaluated over 30 independent runs, and the results are summarized using the mean, standard deviation, best, and worst objective values.

The experiments are implemented in MATLAB R2024a, with each original comparison conducted on the same computer under the same software environment. The supplementary experiments use a computer equipped with an Intel Core i9-14900HX processor (2.20 GHz) and 32 GB RAM, running 64-bit Windows 11. No random seed was explicitly fixed in the original experiments, and identical seeds or initial populations were not enforced across algorithms. MSESFOA initializes its population uniformly at random within the prescribed search bounds using MATLAB’s rand function, while the competing algorithms retain their respective initialization procedures. The WSN experiments in Section 5 also use 30 independent runs for each deployment case.

### 4.1. Description of Benchmark Functions

#### 4.1.1. Description of IEEE CEC 2017 Benchmark Functions

CEC 2017 is a widely adopted benchmark suite with relatively high complexity and is designed to evaluate the performance of swarm intelligence algorithms under challenging environments. It consists of 29 single-objective functions (F1–F30, with F2 excluded), covering a variety of structural characteristics and difficulty levels. Specifically, F1 and F3 are unimodal functions used to assess the exploitation ability of algorithms; F4–F10 are simple multimodal functions that avoid local optima and enhance exploration; F11–F20 are hybrid functions that combine features of multiple basic functions to test both exploration and exploitation simultaneously; and F21–F30 are composition functions that integrate multiple functions to construct highly complex and challenging landscapes.

For fairness and rigor, all the experiments on CEC 2017 are conducted with a search dimension of 30, a population size of 100, and a maximum number of function evaluations of 300,000. Each function has a known theoretical optimum defined as 100×i for the *i*-th function. This benchmark suite provides a comprehensive evaluation of the proposed MSESFOA in terms of its search ability, convergence speed, and robustness when complex optimization problems are solved. The detailed information for each function in the CEC 2017 benchmark suite is summarized in Table 2.

#### 4.1.2. Description of IEEE CEC 2022 Benchmark Functions

The CEC 2022 benchmark function set was designed for the IEEE Congress on Evolutionary Computation to comprehensively evaluate the performance of evolutionary algorithms in diverse and complex environments. This benchmark includes unimodal, basic, hybrid, and composition functions, each presenting unique optimization challenges to test the robustness and adaptability of algorithms. In addition, the test suite incorporates scalability tests, the ability to escape from local optima, and the handling of high-dimensional search spaces, thereby supporting a comprehensive evaluation of the practical capability of optimization algorithms.

In this study, the CEC 2022 functions are tested with a unified setting of 20 dimensions, a population size of 100, and a maximum of 1000 iterations. Notably, this configuration is smaller than the official CEC guideline of one million function evaluations for 20-dimensional problems, but it is more consistent with realistic resource constraints in practical applications. The specific details of each function in the CEC 2022 benchmark set are shown in Table 3.

### 4.2. Results, Analysis and Discussion

To systematically evaluate the global optimization capability and environmental adaptability of the proposed MSESFOA, the CEC 2017 and CEC 2022 benchmark functions are employed for numerical experiments, and the corresponding results are presented and analyzed in this section.

#### 4.2.1. Results and Analysis for IEEE CEC 2017

On the basis of the CEC 2017 benchmark functions, the proposed MSESFOA is compared with the original SFOA and 14 recently developed representative swarm intelligence optimization algorithms. A total of 29 single-objective benchmark functions, covering four categories, including unimodal, simple multimodal, hybrid, and composition functions, are employed, which enables a comprehensive evaluation of the global search capability and robustness of the algorithms.

According to the statistical results reported in Table A1, Table A2, Table A3, Table A4, Table A5, Table A6, Table A7 and Table A8 in Appendix A, the MSESFOA clearly outperforms the other 15 algorithms on the 29 CEC 2017 benchmark functions. Specifically, the MSESFOA achieves an average rank of 1.2759 and thus firmly occupies first place in the overall ranking. In contrast, EAO, ALA, ISAO, and SAO rank second to fifth, with average ranks in the range of approximately 3.17 to 5.69, whereas PKO, TTAO, SFOA, and RIME form a middle tier, with average ranks in the range of 6.79 to 7.66. The average ranks of COA, IRIME, BKA, SHO, NRBO, and IVY generally exceed 10, indicating a considerable gap in overall optimization performance compared with the MSESFOA.

**Figure 1:**
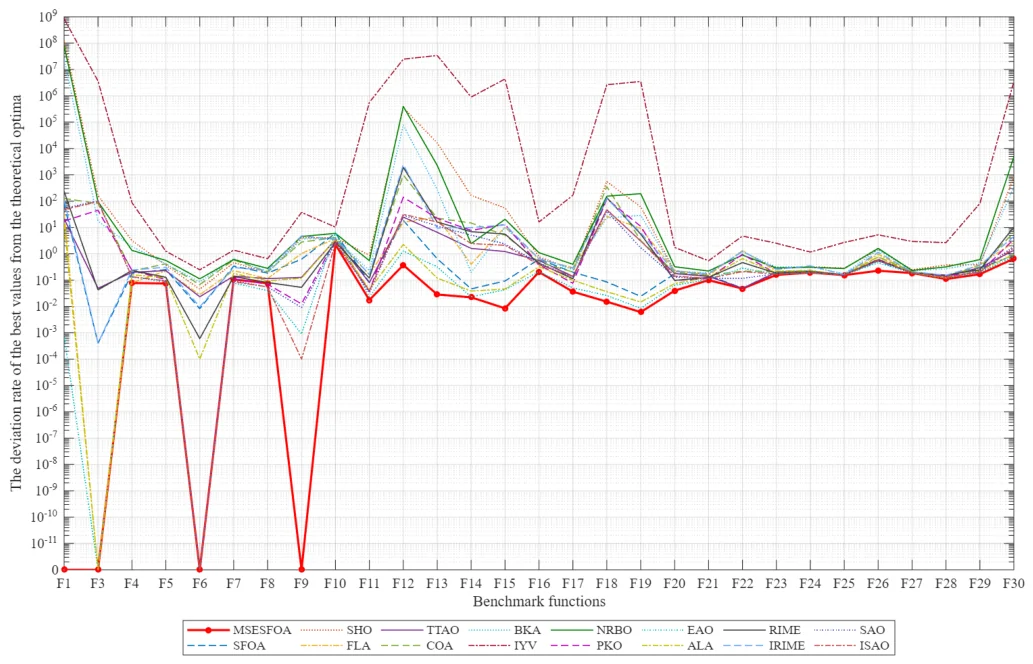
The deviation rate of the mean values from the theoretical optima based on the CEC 2017 benchmark functions.

**Figure 2:**
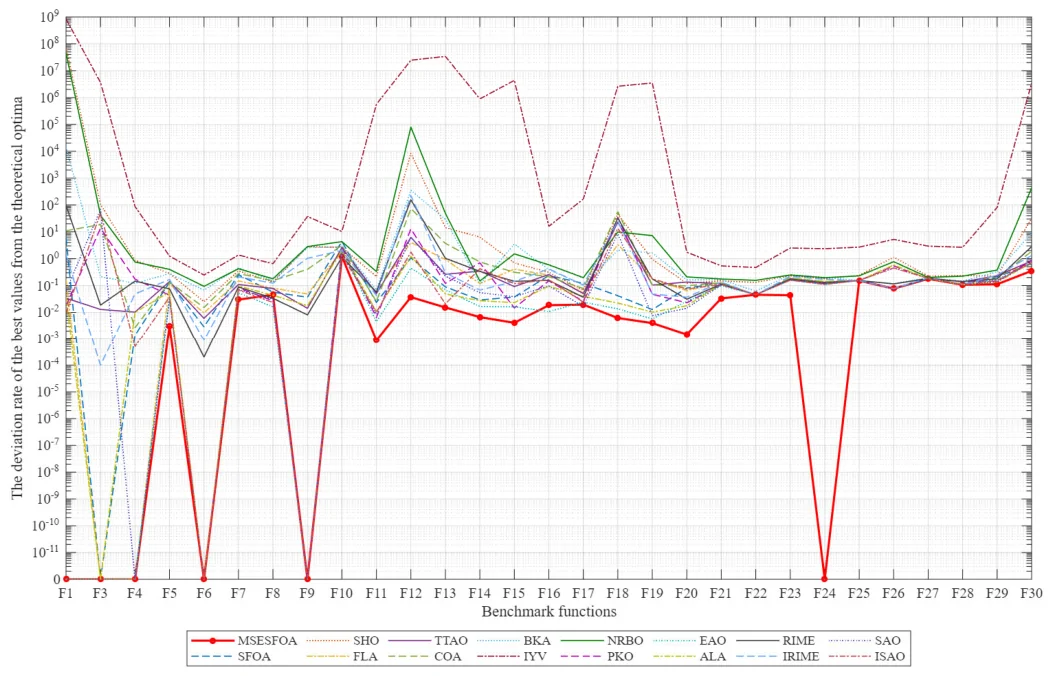
The deviation rate of the best values from the theoretical optima based on the CEC 2017 benchmark functions.

The deviation rate curves shown in Figure 1 and Figure 2 provide a further perspective for assessing the performance of all the algorithms based on the CEC 2017 benchmark functions. For each benchmark function, the deviation rates of the mean and best fitness values are defined as follows:

(26) $$Dev_{mean}\left(i,j\right)=\frac{mean\left(i,j\right)-opt\left(i\right)}{opt\left(i\right)},$$

(27) $$Dev_{best}\left(i,j\right)=\frac{best\left(i,j\right)-opt\left(i\right)}{opt\left(i\right)},$$

where $Dev_{mean}\left(i,j\right)$ and $Dev_{best}\left(i,j\right)$ denote the deviation rates of the mean and the best fitness value, respectively, obtained by the *j*-th algorithm for the *i*-th benchmark function over 30 independent runs with respect to the theoretical optimum. $mean\left(i,j\right)$ and $best\left(i,j\right)$ are the mean and best fitness value, respectively, and $opt\left(i\right)$ is the theoretical optimum of the *i*-th benchmark function.

On the basis of the definitions in Formulas (26) and (27), Figure 1 illustrates the deviation rates of the mean values for all 29 CEC 2017 benchmark functions, and Figure 2 reports the deviation rates of the corresponding best fitness values. For most functions, the deviation-rate curves of the MSESFOA, in terms of both the mean and best values, remain at the lowest level among all the compared algorithms. For relatively regular functions such as F1 to F10, the deviation rates are almost zero, and only for a few hybrid and composition functions does a noticeable yet small gap remain between the obtained results and the theoretical optima. These findings indicate that the MSESFOA maintains a strong global search capability while achieving high convergence accuracy and stability for the CEC 2017 benchmark suite.

From the perspective of function categories, first, for the unimodal functions F1 and F3, the mean, best, and worst values of the MSESFOA are almost completely identical to the theoretical optimal values. For example, for F1, the average value over 30 runs is 100.00, with a standard deviation of only 2.87 × 10^−7^, and both the best and worst values are 100, ranking first among all the algorithms. For F3, the MSESFOA likewise achieves a stable approximation to the optimal value, fully demonstrating its excellent local exploitation ability and convergence reliability in unimodal environments.

Second, for the simple multimodal functions F4 to F10, the MSESFOA ranks first for most functions, such as F4, F5, F6, F9, and F10, and its rankings for F7 and F8 also remain within the top five. With F5 and F6 as examples, the average values of the MSESFOA are 5.37 × 10^2^ and 6.00 × 10^2^, respectively, which are significantly better than those of the SFOA and the other compared algorithms. Moreover, the relatively small standard deviations indicate that the algorithm can effectively avoid being trapped at local minima in multimodal search spaces while maintaining good solution stability and robustness.

Furthermore, for the hybrid functions F11 to F20, the MSESFOA achieves the best performance for most functions, with both the average and best deviations from the theoretical optimum remaining at markedly low levels. In contrast, the performance of the SFOA and some recently proposed algorithms fluctuates considerably for these functions, and in some cases, their mean and best values are several orders of magnitude from the theoretical optimum. This finding indicates that the MSESFOA possesses both a strong global jumping capability and robust local convergence performance in highly complex energy landscapes.

Finally, for the composite functions F21 to F30, the MSESFOA still maintains strong competitiveness. For most composition functions, MSESFOA achieves the best mean performance with relatively small standard deviations, indicating favorable solution quality and consistency across independent runs on these complex problems. For most composite functions, the other algorithms yield larger mean and best values and overall ranks lower that of the MSESFOA, which is consistent with the elevated deviation curves of these algorithms observed in Figure 1 and Figure 2.

To assess overall performance differences across CEC2017, two-sided Wilcoxon signed-rank tests are conducted between MSESFOA and each competing algorithm. Each algorithm is represented by 29 mean final fitness values, one for each tested function, calculated from 30 independent runs. Observations are paired by function, with F2 excluded; the 30 run-level results are not paired across algorithms. For each comparison, the difference is defined as the competing algorithm’s mean fitness minus that of MSESFOA, so a positive difference favors MSESFOA. Exact zero differences are excluded, and tied absolute differences receive average ranks. Two-sided *p*-values are obtained from the exact sign-permutation distribution of the nonzero ranked differences. Holm correction is applied to the 15 comparisons within the suite, with a family-wise significance level of 0.05. Table 4 reports the positive and negative rank sums, unadjusted *p*-values, and Holm-adjusted *p*-values.

As shown in Table 4, all 15 Holm-adjusted *p*-values are below 0.05, and the positive rank sums exceed the negative rank sums in every comparison. These results provide statistical evidence of an overall performance advantage for MSESFOA across the tested CEC2017 functions. They do not imply that MSESFOA outperforms every competing algorithm on every individual function.

#### 4.2.2. Results and Analysis for IEEE CEC 2022

For the CEC 2022 benchmark functions, the MSESFOA is again compared with the original SFOA and 14 other algorithms, which are the same as those compared on the basis of CEC 2017. This test suite consists of 12 single-objective functions covering four categories, namely, unimodal, basic multimodal, hybrid, and composite functions. The results are shown in Table A9, Table A10, Table A11 and Table A12 in Appendix B.

As reported from Table A9, Table A10, Table A11 and Table A12, the MSESFOA achieves the best overall performance, with a mean rank of 1.50 and first place in the final ranking. EAO, ISAO, and SAO rank second to fourth, whereas ALA and RIME form a strong second tier. In contrast, the SFOA, TTAO, and COA generally yield medium-level performance, and algorithms such as SHO, BKA, NRBO, and IVY significantly lag behind the MSESFOA. Overall, the MSESFOA maintains a leading advantage for the CEC 2022 suite, similar to that observed for CEC 2017.

From the perspective of function categories, the MSESFOA performs particularly well for the unimodal and basic functions (F1 to F5). For these problems, the mean, best, and worst fitness values are very close to the theoretical optima, and the standard deviations are consistently small. For instance, for the unimodal function F1, the mean fitness of MSESFOA over 30 runs is approximately 300, with a standard deviation of 4.65 × 10^−4^; both the best and worst fitness values round to 300.00. These results indicate a close approximation to the theoretical optimum. For the basic functions F2 to F5, the MSESFOA also exhibits small deviations and high ranks, indicating that it preserves strong local exploitation ability and convergence accuracy in the CEC 2022 scenarios.

Second, for the hybrid functions F6 to F8, the MSESFOA achieves the best performance for most functions, and its mean and best fitness values are closer to the theoretical optima and its standard deviations remain relatively small compared with those of the original SFOA and other algorithms. These findings suggest that the MSESFOA has a strong global jumping ability. Finally, for the composition functions F9 to F12, the performance gap among the algorithms becomes more pronounced: the MSESFOA still ranks within the top positions for most functions and ranks first for some of them, whereas several competitors yield mean and best values that are one or more orders of magnitude away from the theoretical optima, with large variances. Overall, the mean fitness values and standard deviations indicate competitive solution quality and consistency across independent runs on the hybrid and composition functions of CEC2022.

**Figure 3:**
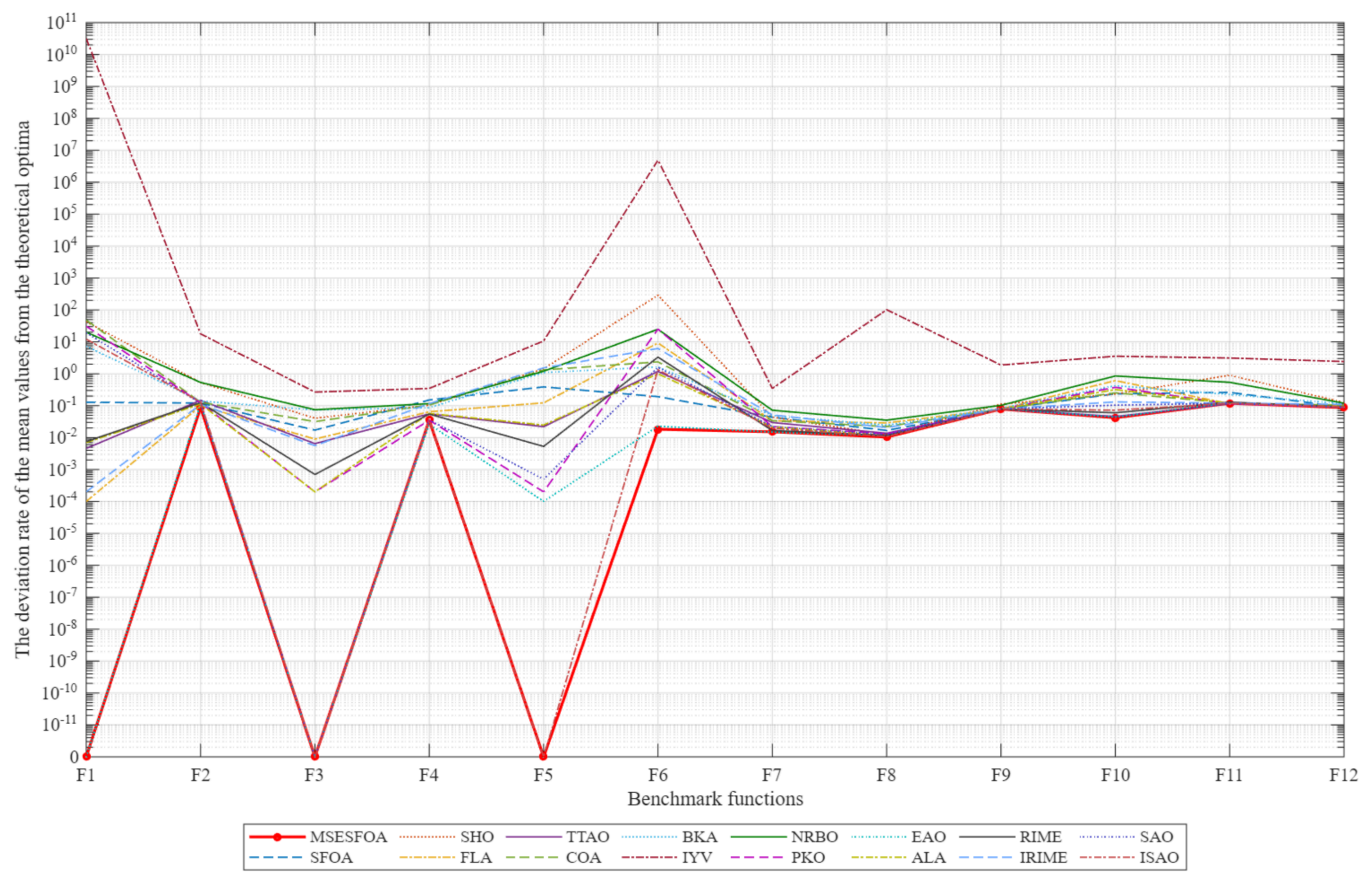
The deviation rate of the mean values from the theoretical optima based on the CEC 2022 benchmark functions.

**Figure 4:**
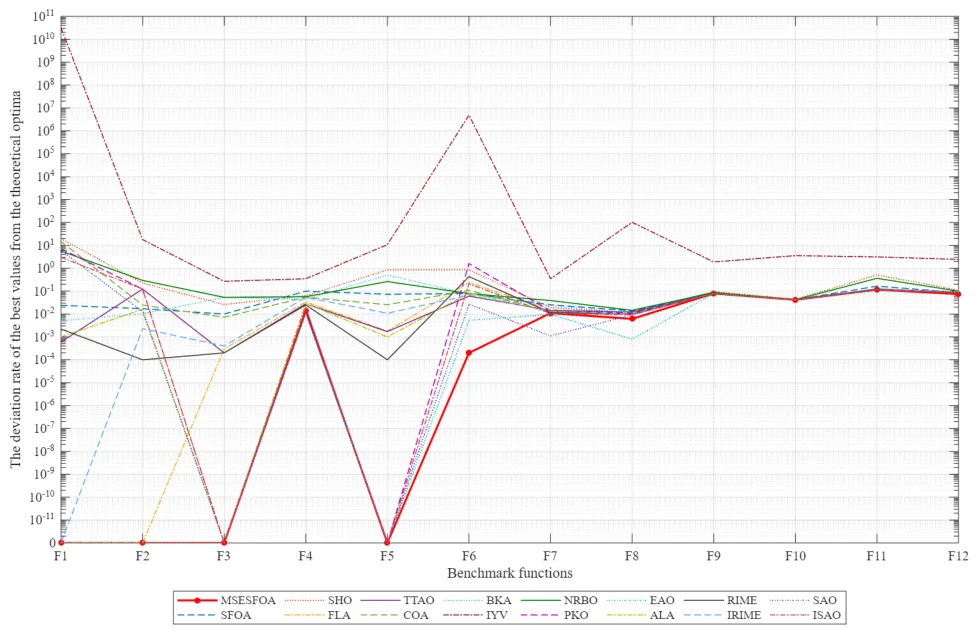
The deviation rate of the best values from the theoretical optima based on the CEC 2022 benchmark functions.

Figure 3 and Figure 4 further illustrate the performance of all the algorithms for the CEC 2022 benchmark functions in terms of deviation-rate curves. In accordance with the definitions in Formulas (26) and (27), Figure 3 reports the deviation rates of the mean fitness values over 30 independent runs for the 12 benchmark functions, whereas Figure 4 presents the corresponding deviation rates of the best fitness values. For most benchmark functions, both the mean and best deviation–rate curves of the MSESFOA remain at the lowest level and are at the lowest among all the competing algorithms. For only a few hybrid or composite functions do the curves of the MSESFOA increase slightly, leading to a modest gap with those of some competitors, but the overall magnitude of the deviation is still small. These trends are consistent with the statistical comparisons in Table A9, Table A10, Table A11 and Table A12, further confirming that the MSESFOA achieves high convergence accuracy and stability for the CEC 2022 benchmark suite.

To assess the statistical significance of the performance differences on CEC2022, a two-sided Wilcoxon signed-rank test is conducted using the mean final fitness values over 30 independent runs for each of the 12 benchmark functions. Each benchmark function constitutes one paired observation. The differences are defined as the competing algorithm’s mean fitness minus that of MSESFOA, so positive differences favor MSESFOA. Exact zero differences are excluded, and tied absolute differences receive average ranks. Two-sided *p*-values are obtained from the exact sign-permutation distribution of the nonzero ranked differences. Holm correction is applied to the 15 comparisons within CEC2022 at a family-wise significance level of 0.05. Table 5 reports the positive and negative rank sums, unadjusted *p*-values, and Holm-adjusted *p*-values.

After Holm correction, statistically significant differences are observed between MSESFOA and 14 of the 15 competing algorithms, with the rank sums favoring MSESFOA. The comparison with EAO is not significant at the 0.05 level (adjusted *p* = 0.0830), whereas the comparison with PKO remains significant (adjusted *p* = 0.0273). These results support an overall performance advantage of MSESFOA across the tested CEC2022 functions against most competitors, while providing insufficient evidence of a statistically significant difference from EAO.

### 4.3. Ablation Study and Parameter Sensitivity Analysis

To examine the contributions of the three enhancement mechanisms and the effects of selected parameter settings, ablation and parameter sensitivity experiments are conducted on both CEC2017 and CEC2022. The experiments use a population size of 100, with 30 dimensions and 3000 iterations for CEC2017 and 20 dimensions and 1000 iterations for CEC2022. Each configuration is evaluated over 30 independent runs. The following subsections describe the experimental configurations and discuss the main findings, while the complete statistical results and boxplots are provided in the appendices.

#### 4.3.1. Ablation Study

**Figure A1:**
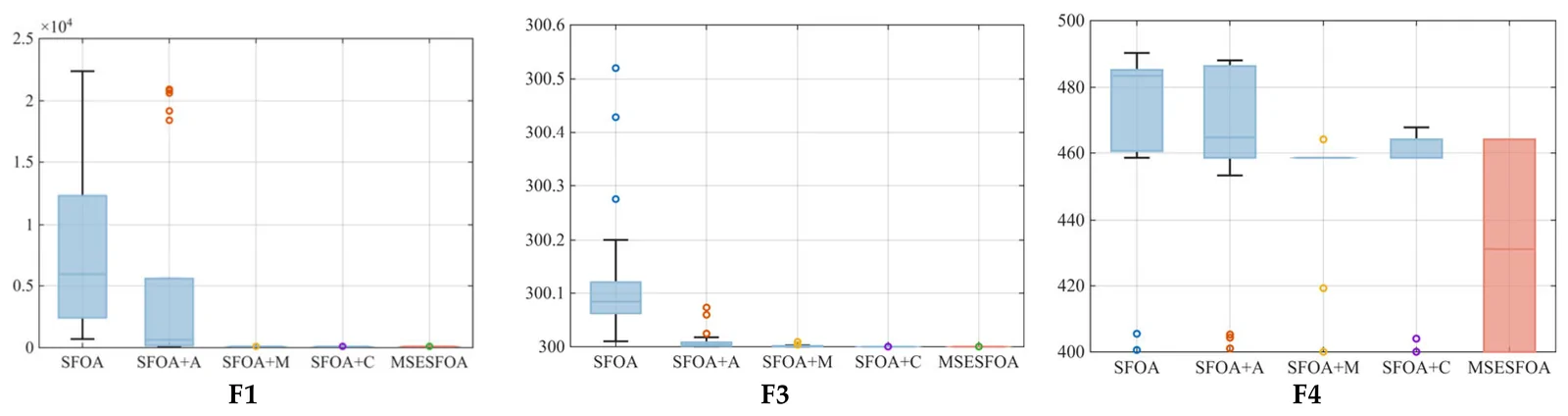

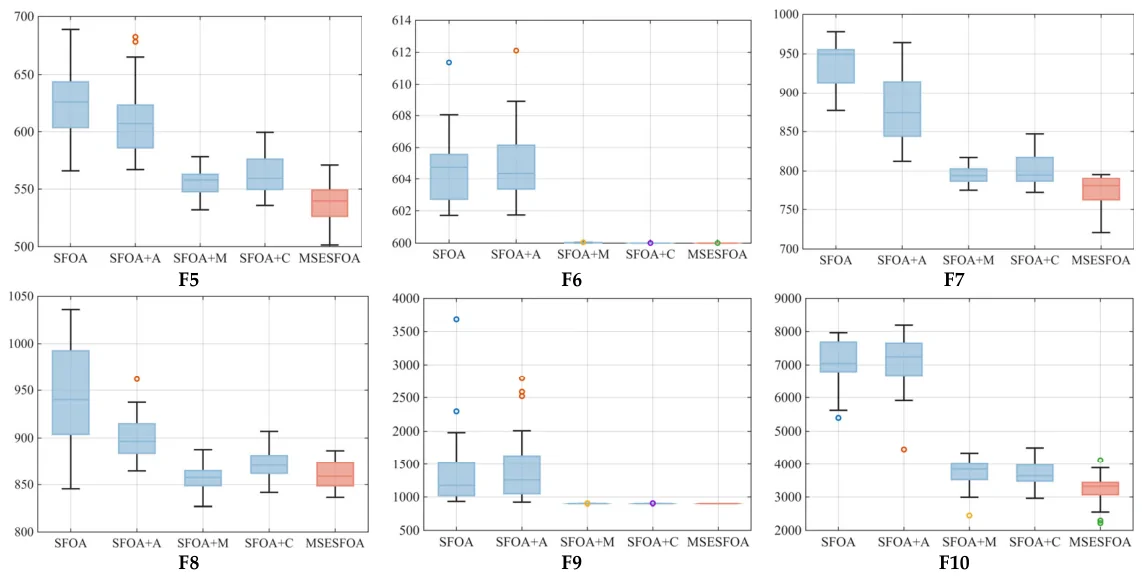
Boxplots of the final fitness values over 30 independent runs for the ablation variants on CEC2017 (F1 and F3–F10).

**Figure A2:**
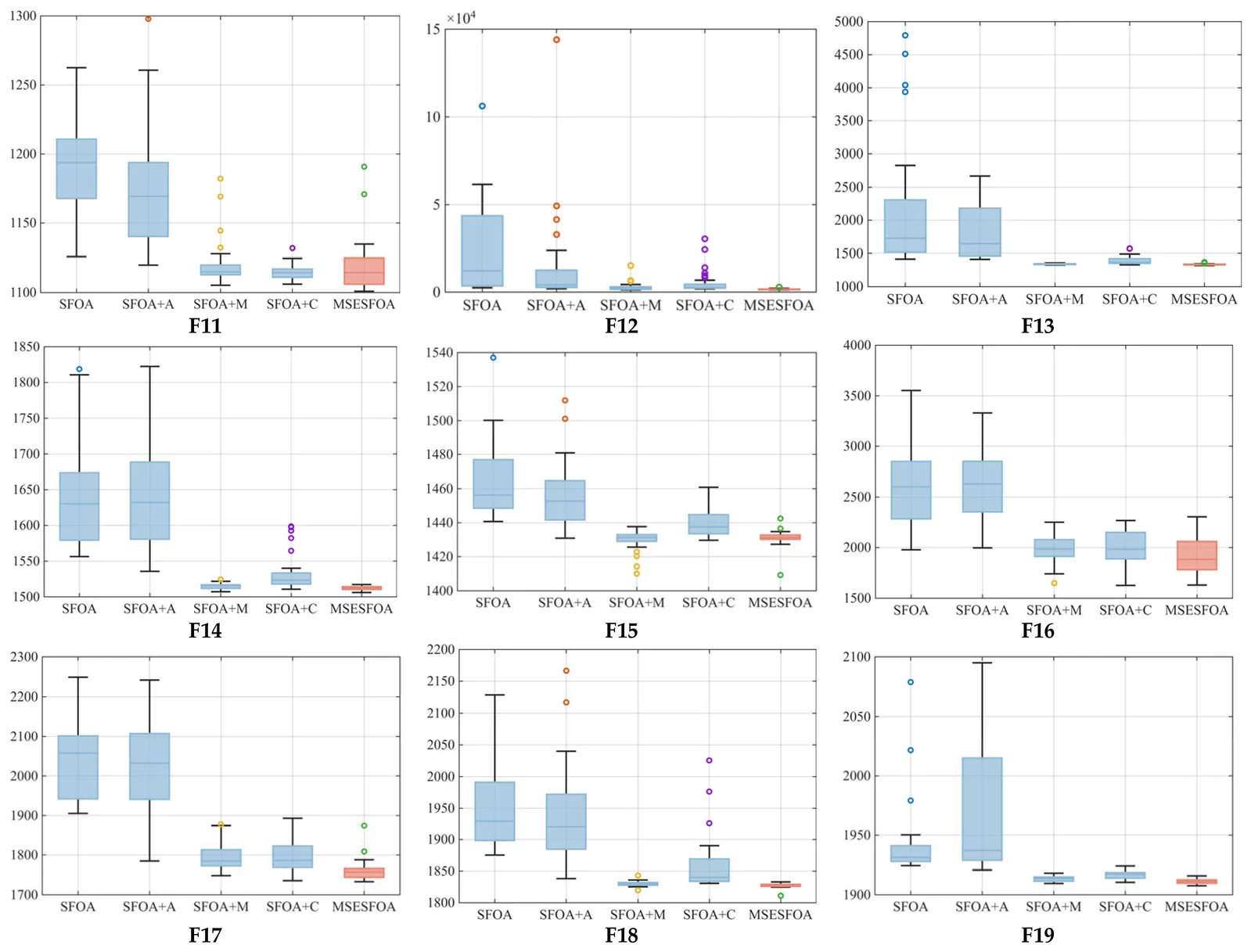

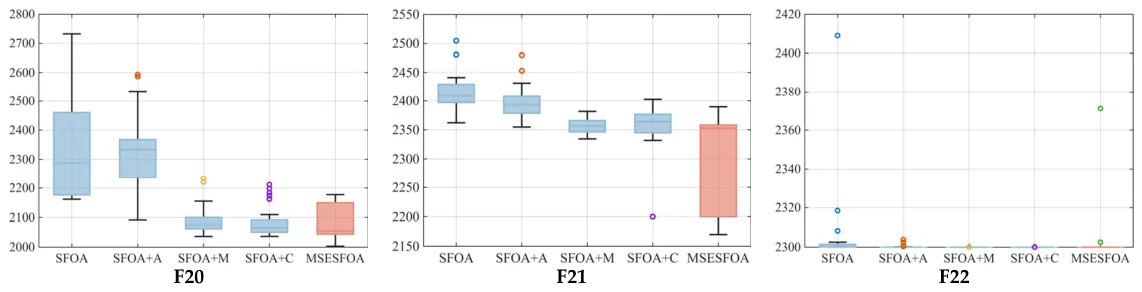
Boxplots of the final fitness values over 30 independent runs for the ablation variants on CEC2017 (F11–F22).

**Figure A3:**
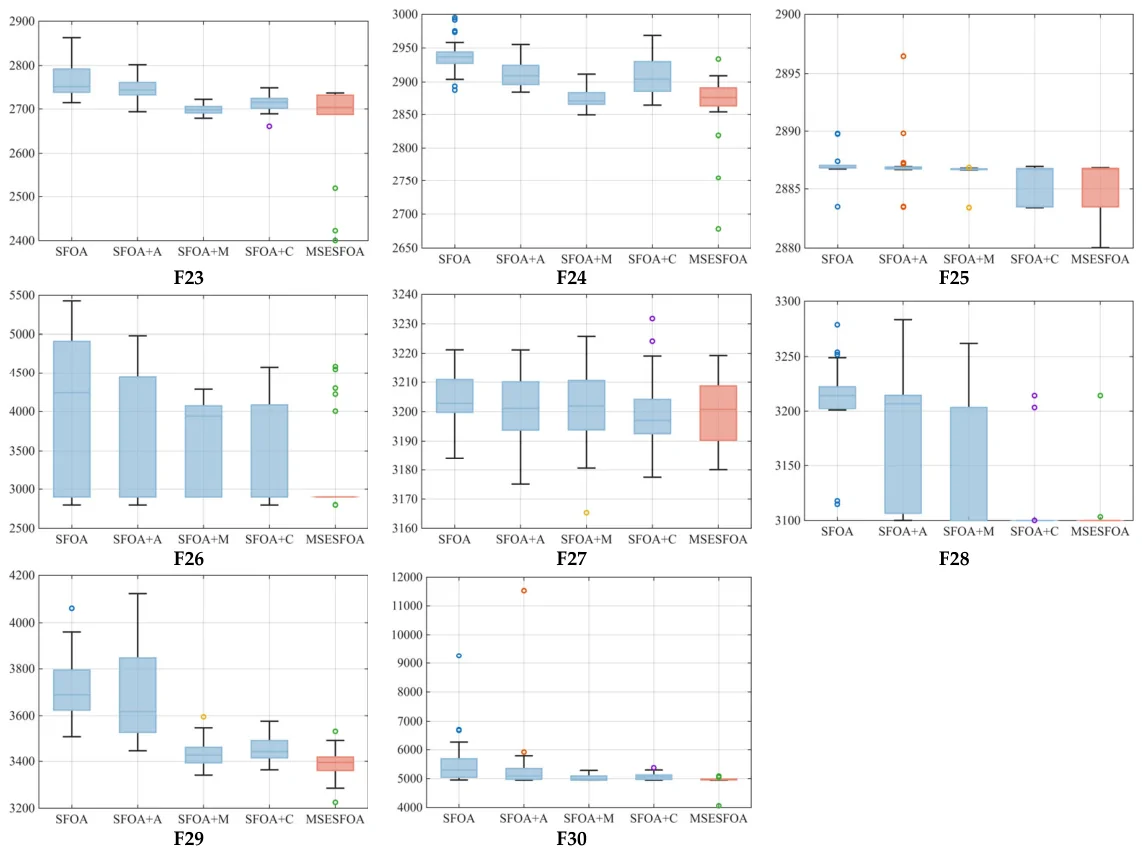
Boxplots of the final fitness values over 30 independent runs for the ablation variants on CEC2017 (F23–F30).

**Figure A4:**
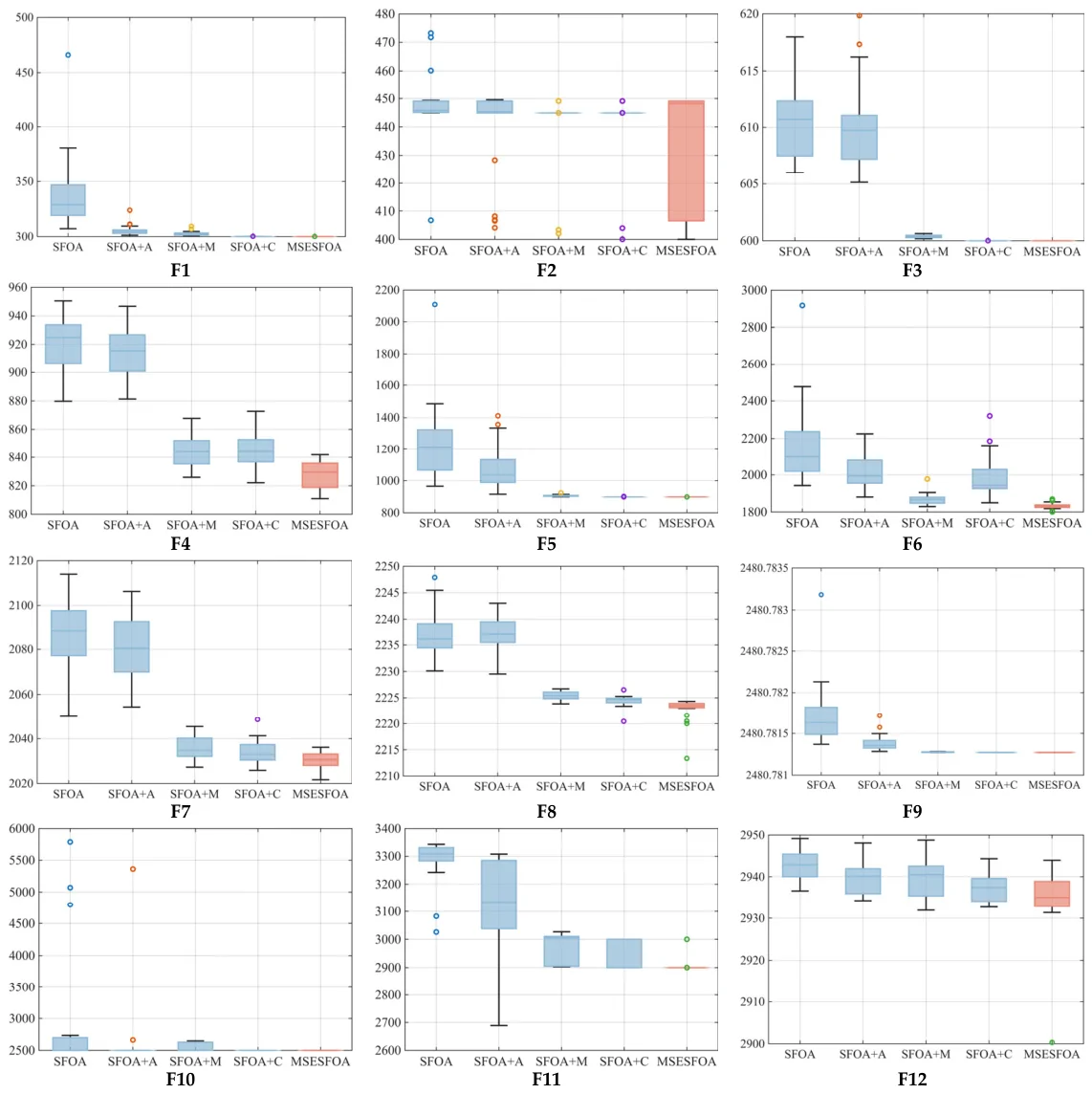
Boxplots of the final fitness values over 30 independent runs for the ablation variants on CEC2022.

To assess the contributions of the three enhancement mechanisms, five configurations are compared: the basic SFOA, SFOA with adaptive exploration regulation (SFOA+A), SFOA with stage-based combined mutation (SFOA+M), SFOA with multi-arm cooperative recombination (SFOA+C), and the complete MSESFOA. Each single-component variant incorporates only the corresponding enhancement into the basic SFOA. The complete results are provided in Appendix C. For CEC2017, Table A13, Table A14 and Table A15 report the mean, standard deviation, best, and worst final fitness values, together with the rankings, while Figure A1, Figure A2 and Figure A3 present the boxplots of the final fitness values over 30 independent runs. For CEC2022, the corresponding statistical results and rankings are reported in Table A16 and Table A17, and the boxplots are presented in Figure A4. Each configuration is ranked by its mean fitness on each function, with a smaller value indicating better performance. The mean rank across functions summarizes its overall performance within each benchmark suite.

On CEC2017, MSESFOA obtains the best mean fitness on 24 of the 29 tested functions and achieves the best overall mean rank of 1.2414. The mean ranks of SFOA+C, SFOA+M, SFOA+A, and SFOA are 2.4138, 2.4483, 4.0345, and 4.8621, respectively. Thus, all three single-component variants improve the aggregate ranking relative to the basic SFOA, while mutation and cooperative recombination provide larger improvements than adaptive exploration regulation alone. The complete configuration further improves the overall ranking beyond any single-component variant.

The improvements are particularly evident on some difficult functions. For example, on CEC2017 F12, the mean fitness decreases from 2.3425 × 10^4^ for SFOA to 1.4706 × 10^4^, 2.9464 × 10^3^, and 5.4725 × 10^3^ for SFOA+A, SFOA+M, and SFOA+C, respectively, and further decreases to 1.6474 × 10^3^ for MSESFOA. Its standard deviation also decreases to 3.3413 × 10^2^, compared with 2.4467 × 10^4^ for SFOA. However, the complete configuration does not dominate every function: SFOA+M obtains better mean results on F8 and F14, whereas SFOA+C performs better on F11, F22, and F27. These exceptions indicate that the benefits of combining the mechanisms depend on the problem and are not uniformly additive.

On CEC2022, MSESFOA ranks first by mean fitness on all 12 functions, resulting in a mean rank of 1.0000. SFOA+C, SFOA+M, SFOA+A, and SFOA obtain mean ranks of 2.2500, 2.9167, 3.9167, and 4.9167, respectively. Among the single-component variants, cooperative recombination performs best on nine functions, mutation on F4 and F6, and adaptive exploration regulation on F2. This variation shows that the relative contribution of each mechanism differs across functions. Nevertheless, near-identical results should be interpreted cautiously: on F9, the mean fitness values coincide at the displayed precision, so their numerical ranking does not represent a substantial practical difference.

The boxplots provide additional information on run-to-run variability. Lower mean fitness does not always coincide with a smaller standard deviation. For example, on CEC2022 F12, MSESFOA obtains a lower mean and a better best value than SFOA+C, but its standard deviation is larger. Therefore, solution quality and variability are considered separately. Overall, the results support the aggregate performance advantage of the complete MSESFOA under the tested iteration settings, while also revealing function-specific advantages of individual mechanisms and remaining variability across independent runs.

#### 4.3.2. Parameter Sensitivity Analysis

**Figure A5:**
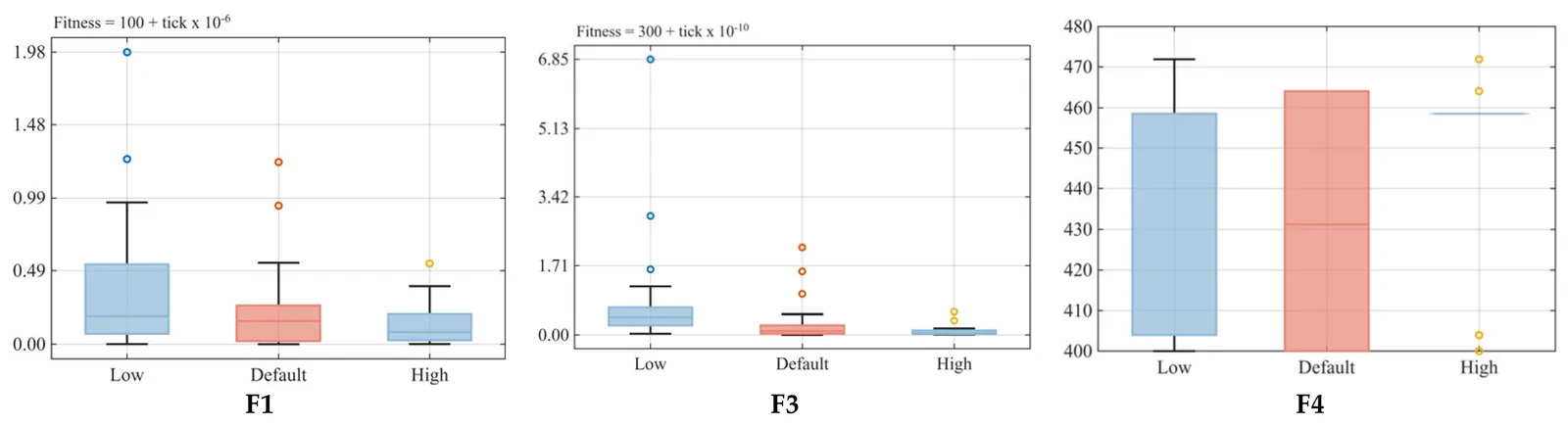

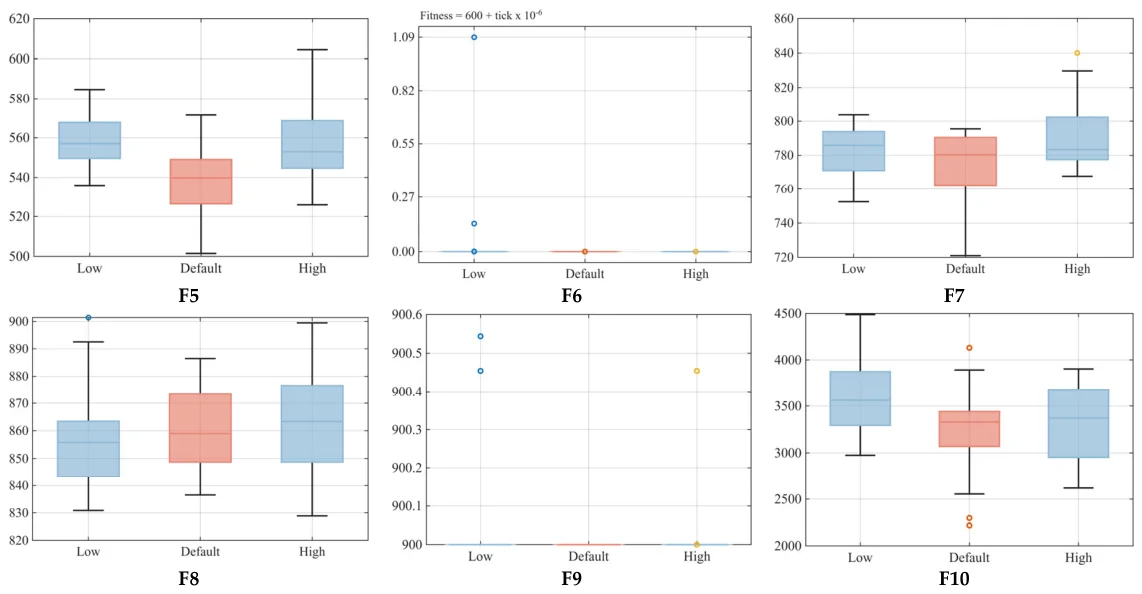
Boxplots of the final fitness values over 30 independent runs under three mutation probability settings on CEC2017 (F1 and F3–F10).

**Figure A6:**
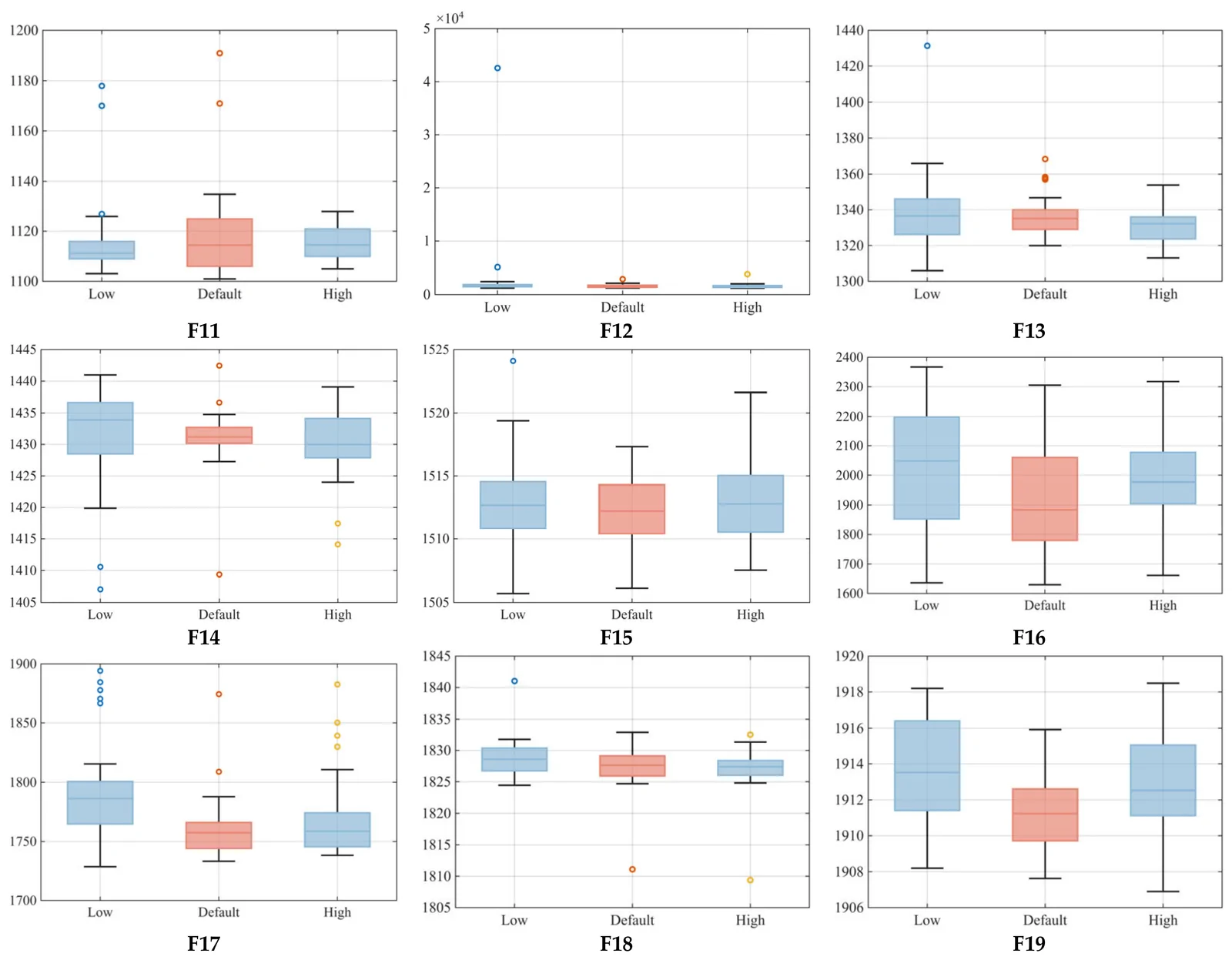

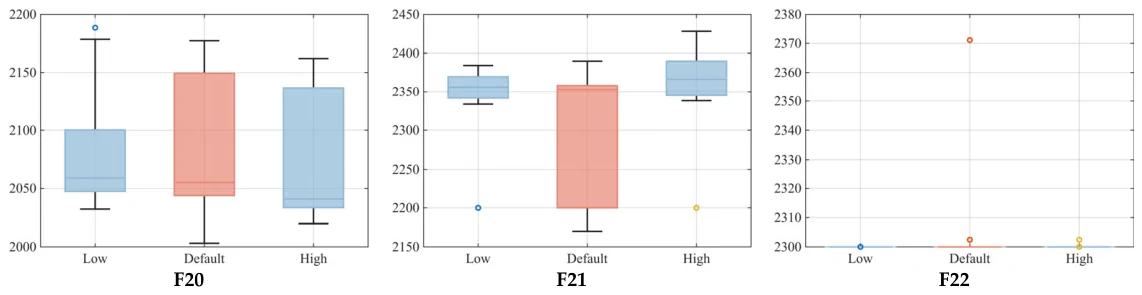
Boxplots of the final fitness values over 30 independent runs under three mutation probability settings on CEC2017 (F11–F22).

**Figure A7:**
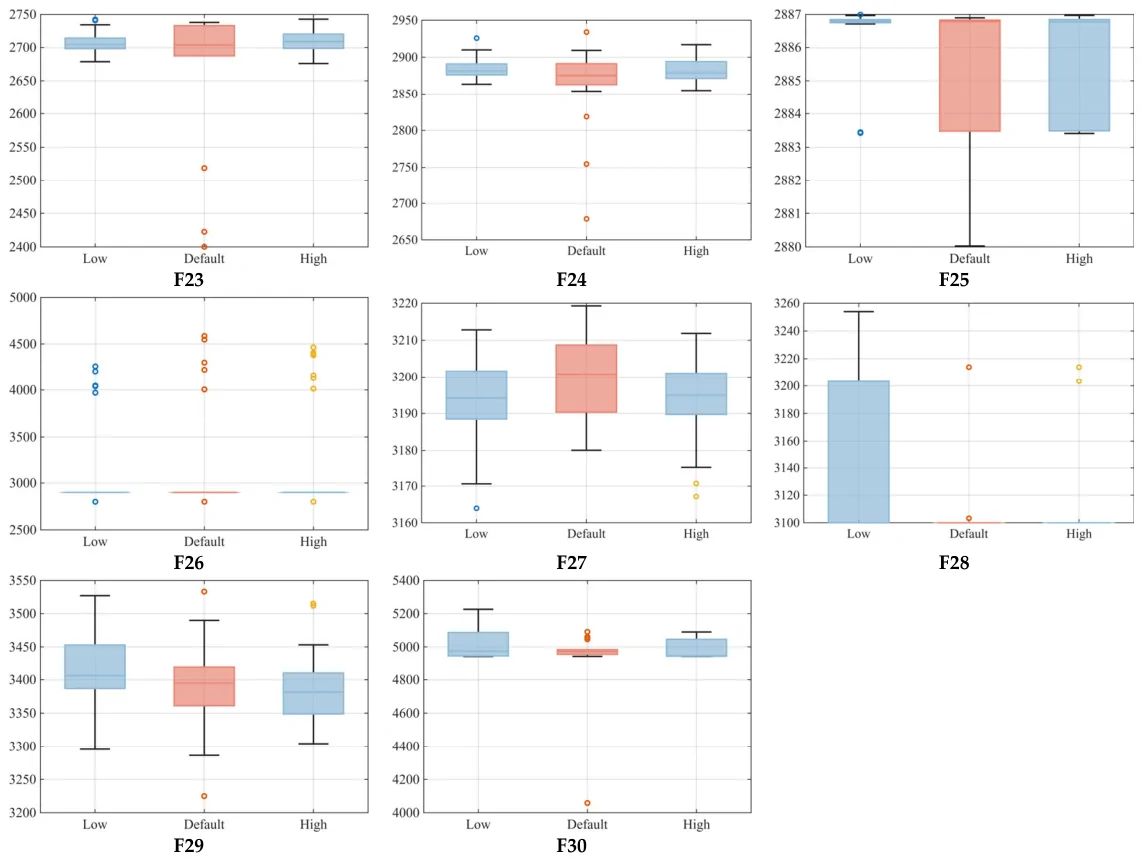
Boxplots of the final fitness values over 30 independent runs under three mutation probability settings on CEC2017 (F23–F30).

**Figure A8:**
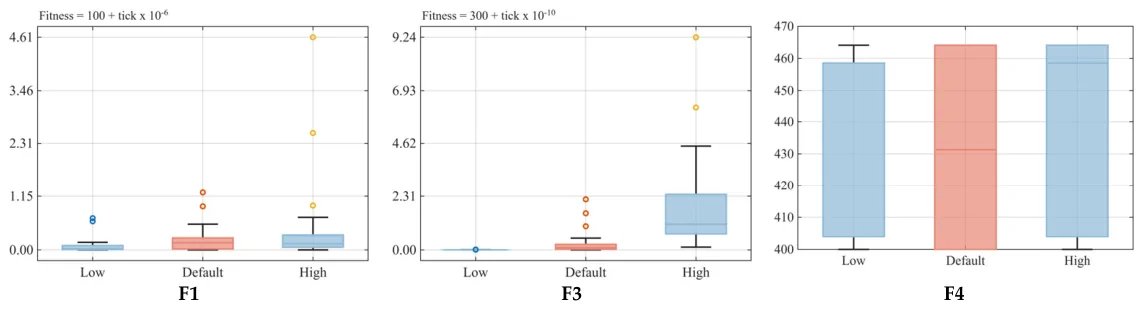
Boxplots of the final fitness values over 30 independent runs under three mutation scale F settings on CEC2017 (F1, F3 and F4).

**Figure A9:**
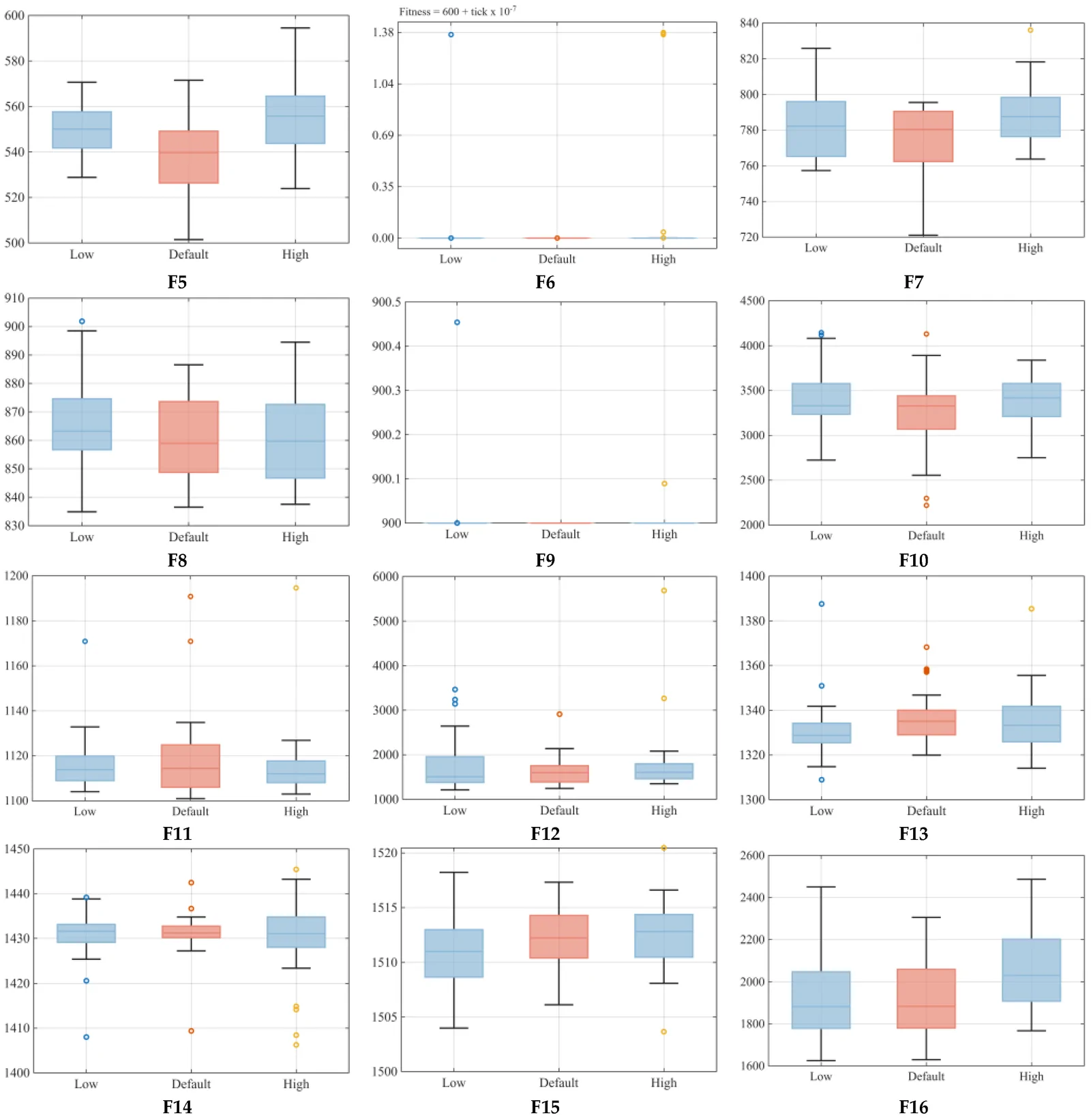
Boxplots of the final fitness values over 30 independent runs under three mutation scale F settings on CEC2017 (F5–F16).

**Figure A10:**
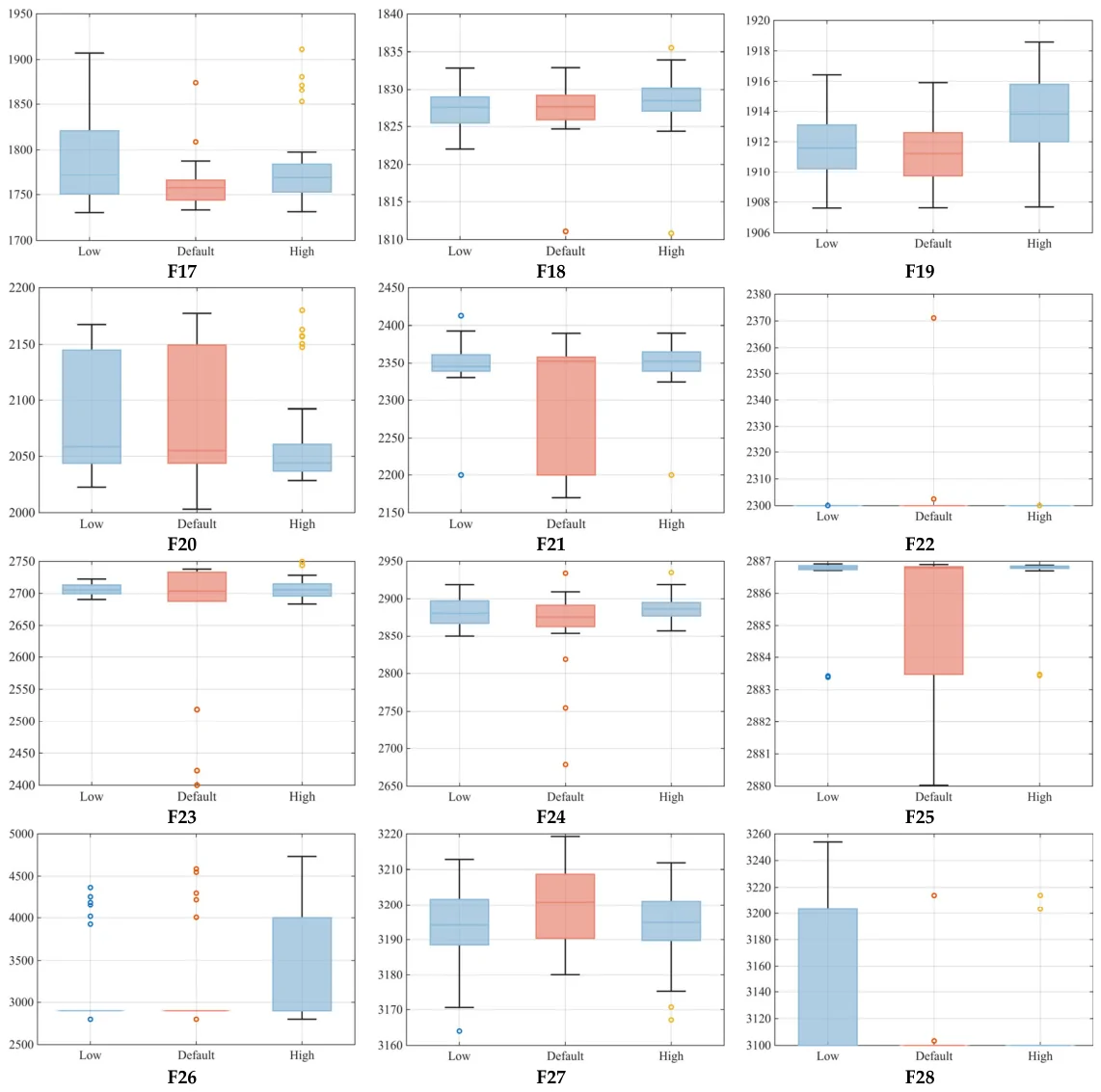
Boxplots of the final fitness values over 30 independent runs under three mutation scale F settings on CEC2017 (F17–F28).

**Figure A11:**
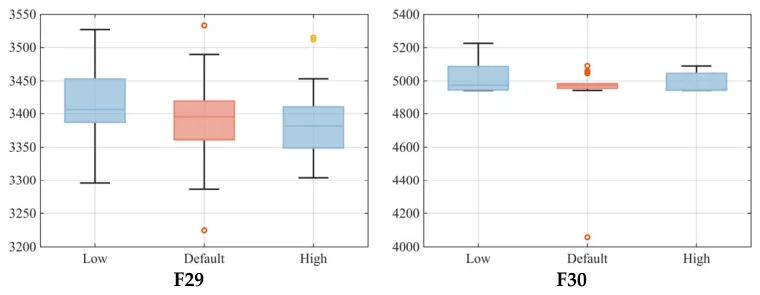
Boxplots of the final fitness values over 30 independent runs under three mutation scale F settings on CEC2017 (F29, F30).

**Figure A12:**
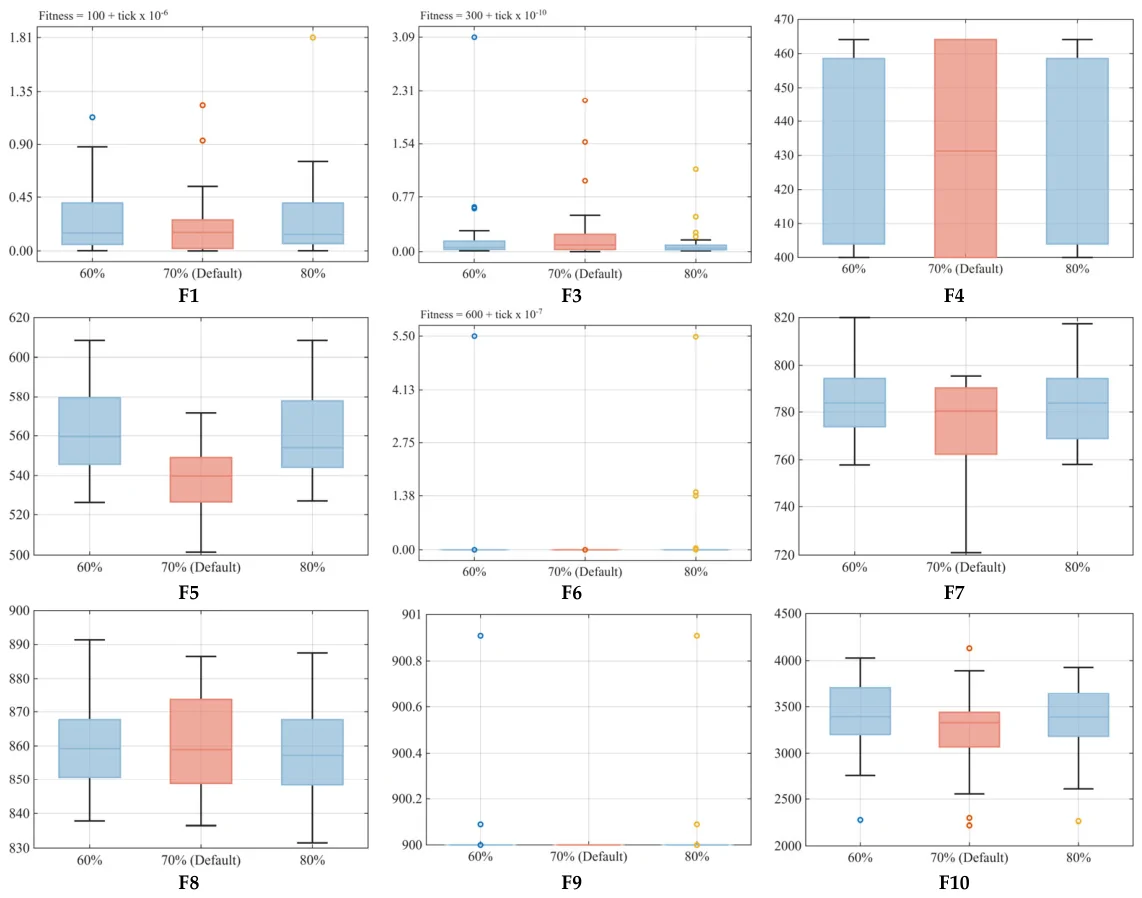
Boxplots of the final fitness values over 30 independent runs under three late-stage mutation onset settings on CEC2017 (F1 and F3–F10).

**Figure A13:**
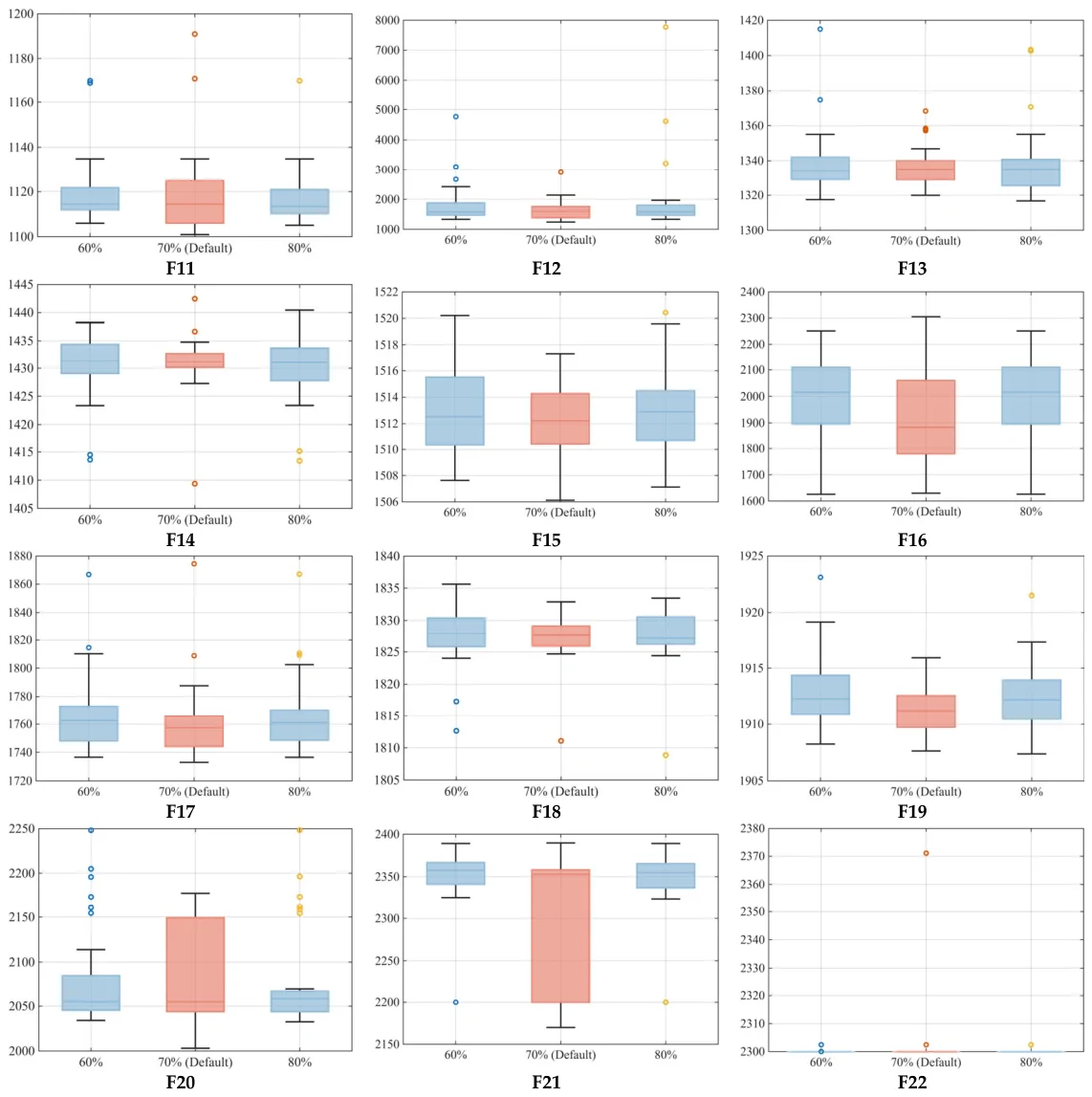
Boxplots of the final fitness values over 30 independent runs under three late-stage mutation onset settings on CEC2017 (F11–F22).

**Figure A14:**
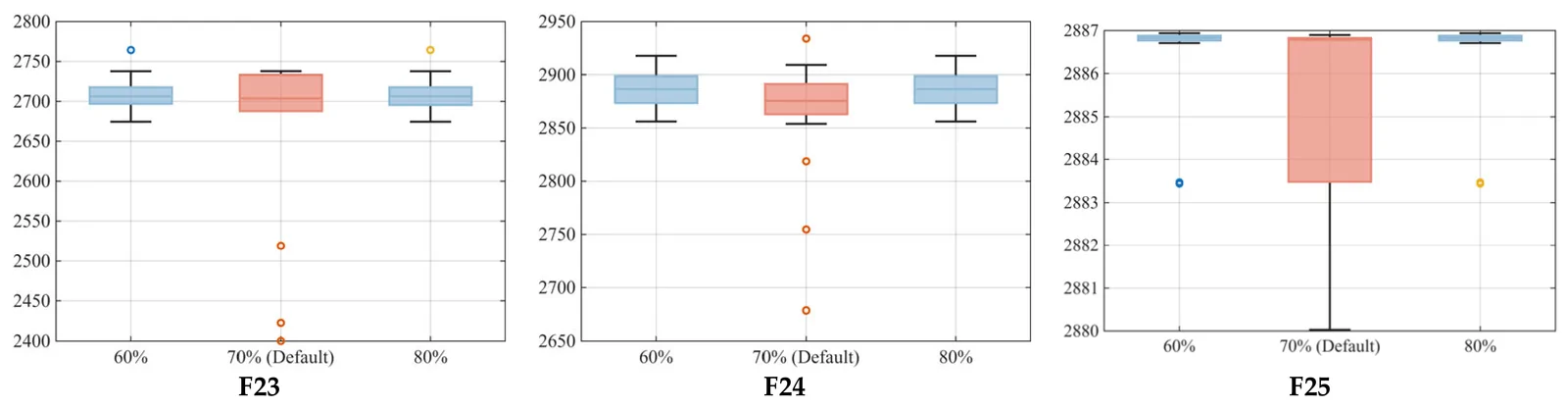

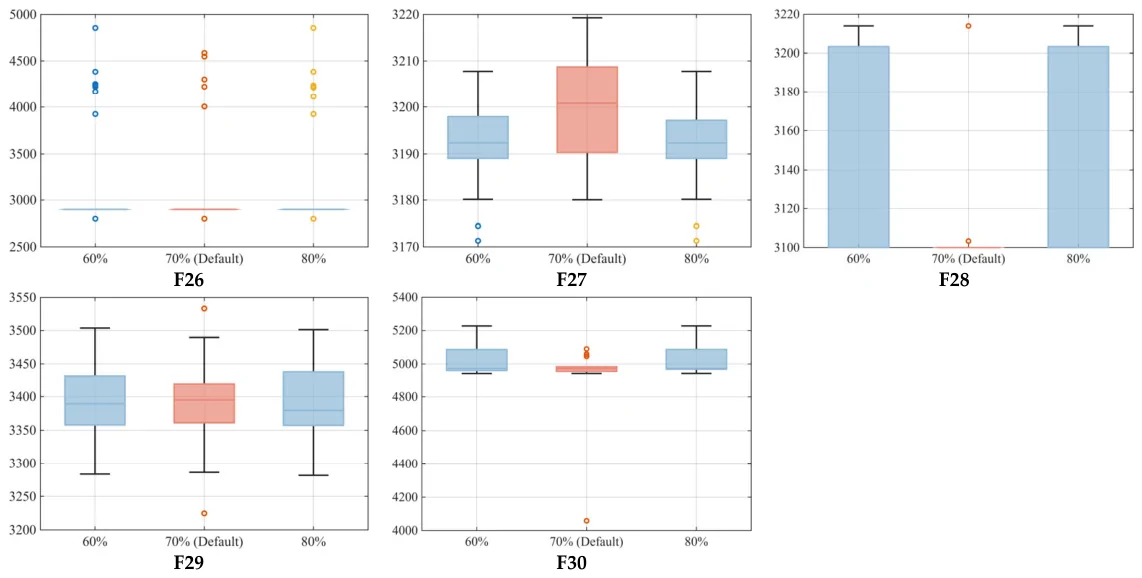
Boxplots of the final fitness values over 30 independent runs under three late-stage mutation onset settings on CEC2017 (F23–F30).

**Figure A15:**
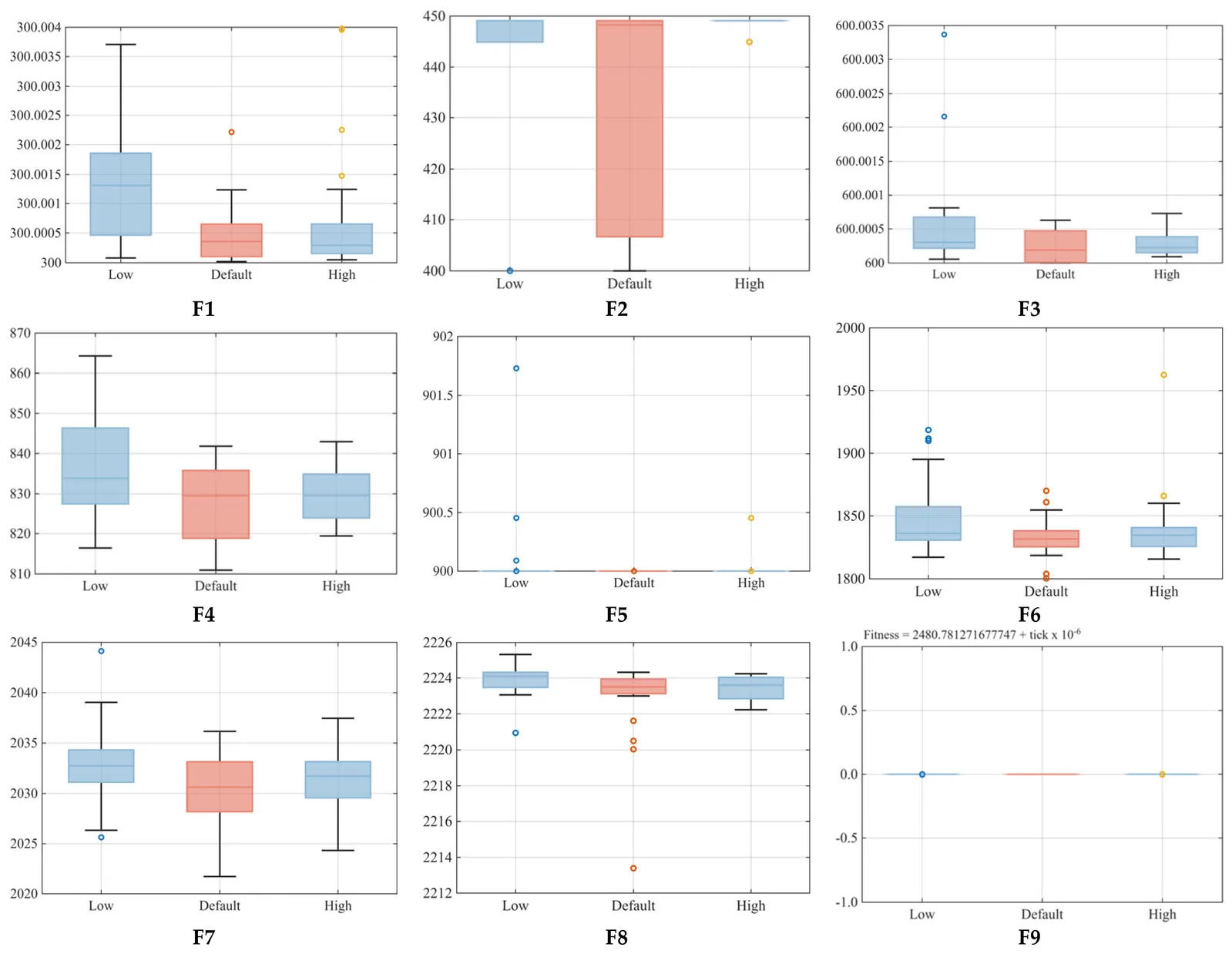

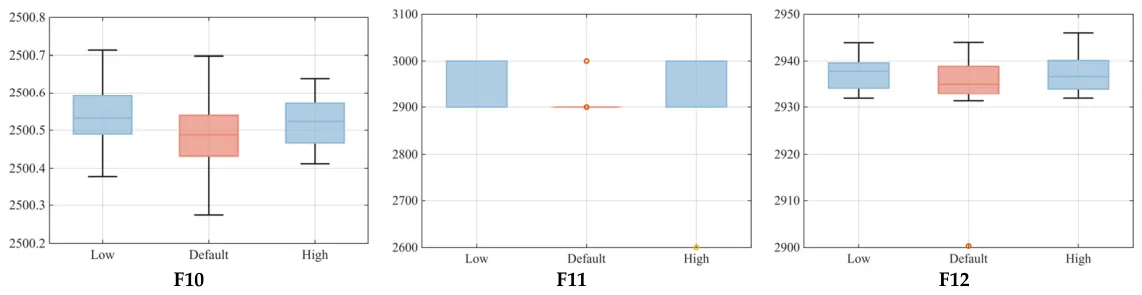
Boxplots of the final fitness values over 30 independent runs under three mutation probability settings on CEC2022.

**Figure A16:**
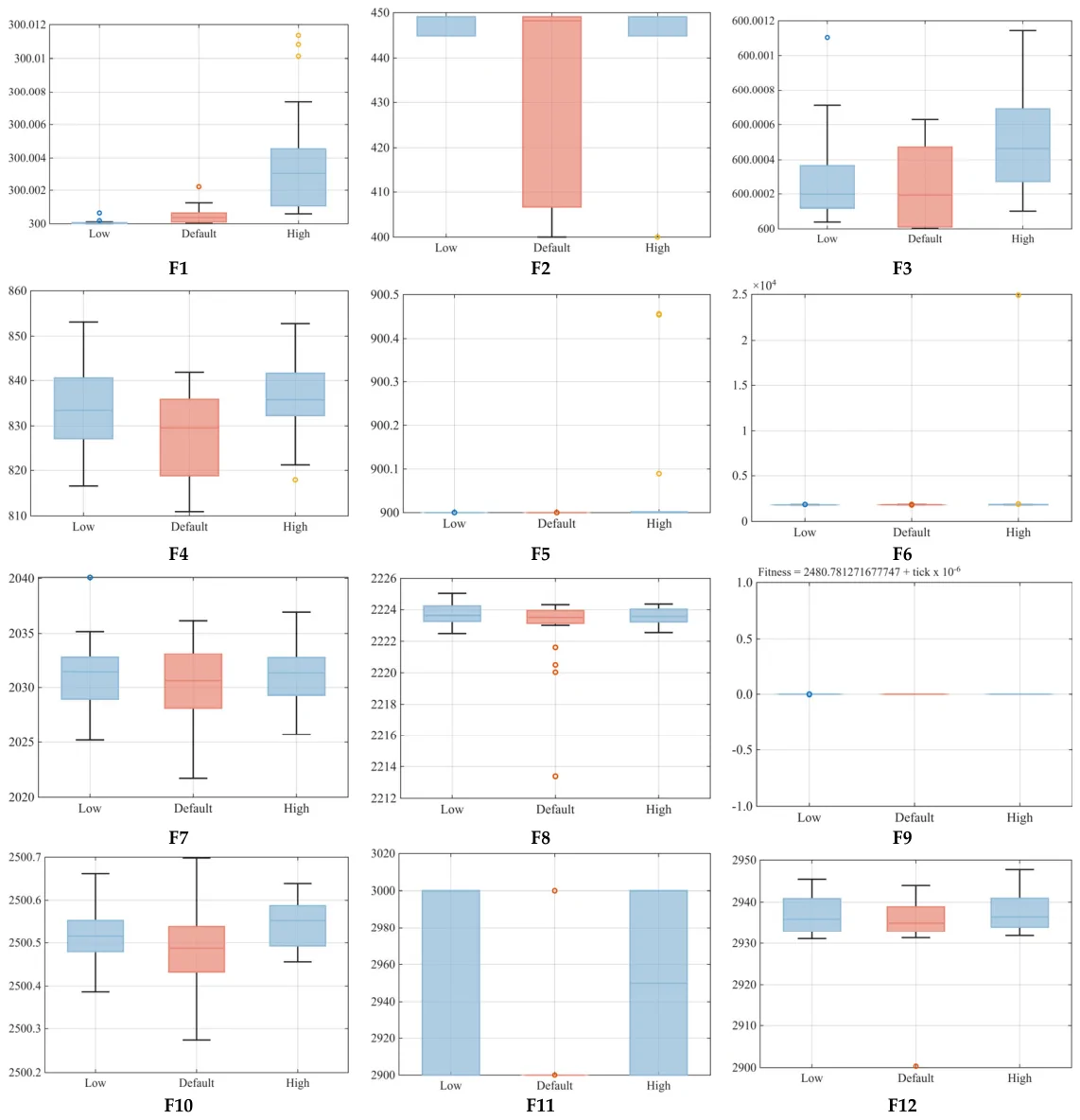
Boxplots of the final fitness values over 30 independent runs under three mutation scale F settings on CEC2022.

**Figure A17:**
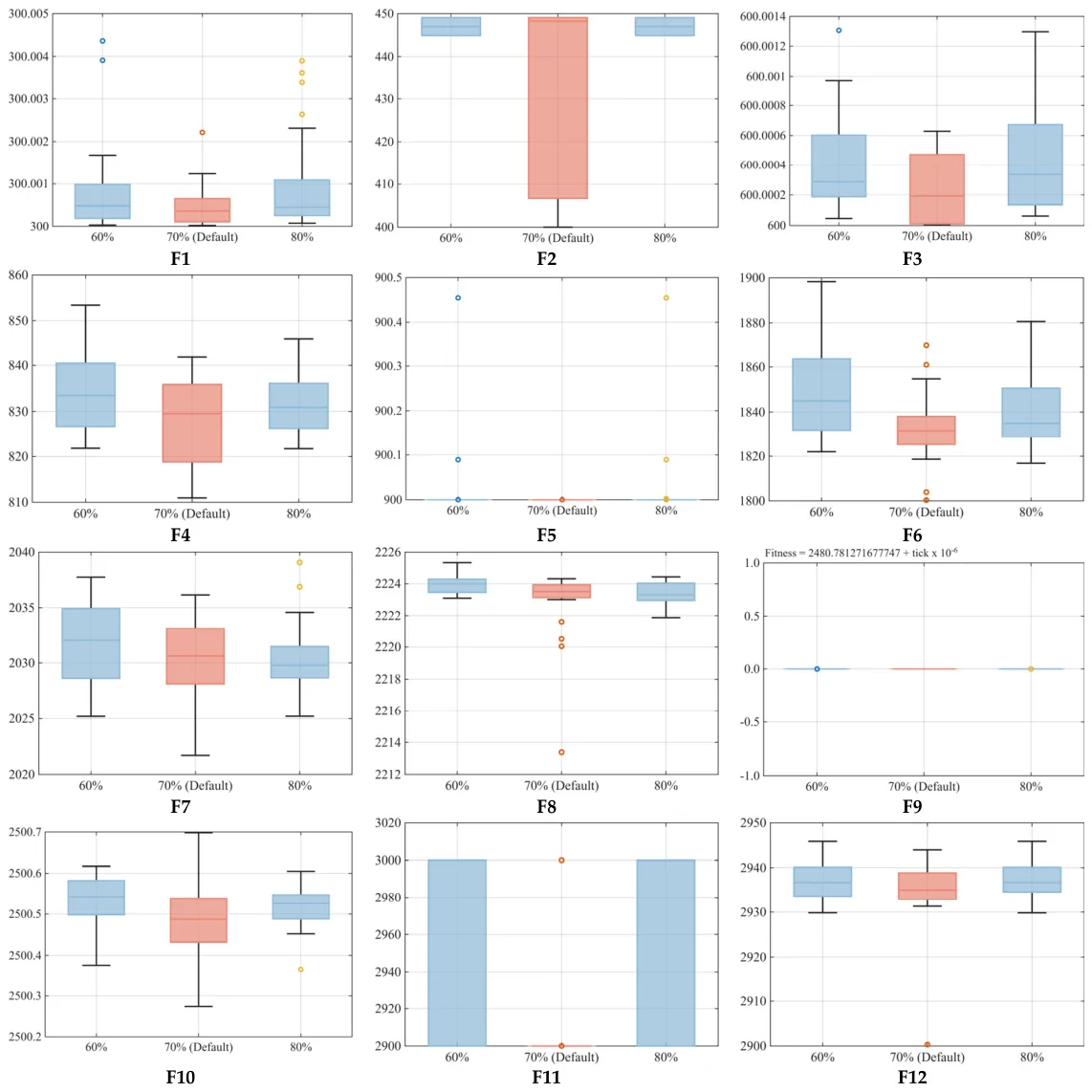
Boxplots of the final fitness values over 30 independent runs under three late-stage mutation onset settings on CEC2022.

To examine the influence of key parameter settings, three groups of sensitivity experiments are conducted on CEC2017 and CEC2022, varying the mutation probability range, mutation scale F, and onset of the late mutation stage separately. The mutation probability ranges are set to [0.05, 0.25], [0.10, 0.40] (Default), and [0.15, 0.55]. The mutation scale settings are F = 0.4 + 0.3r, F = 0.5 + 0.3r (Default), and F = 0.6 + 0.3r, where r is uniformly distributed in [0, 1]. The late mutation stage begins at 60%, 70% (Default), or 80% of the maximum number of iterations. Only the indicated parameter setting is changed in each group; the remaining settings follow the corresponding benchmark experiments. Each configuration is evaluated over 30 independent runs. Table A18, Table A19, Table A20 and Table A22 in Appendix D report the complete statistics for CEC2017 and CEC2022, respectively, while Table A21 and Table A23 provide rankings calculated separately within each parameter group using unrounded mean fitness values. The corresponding boxplots are presented in Figure A5, Figure A6, Figure A7, Figure A8, Figure A9, Figure A10, Figure A11, Figure A12, Figure A13 and Figure A14 for CEC2017 and Figure A15, Figure A16 and Figure A17 for CEC2022.

For the mutation probability range, the default setting achieves the lowest mean rank on both benchmark suites. On CEC2017, the mean ranks of P-low, Default, and P-high are 2.5172, 1.5517, and 1.9310, respectively. Although P-high performs better on several functions, including F11 and F14, increasing the mutation probability does not consistently improve performance across the suite. On CEC2022, the corresponding mean ranks are 2.7917, 1.0000, and 2.2083. These results support the default probability range as a favorable overall choice among the tested settings. However, rank differences associated with very small numerical differences, such as those on CEC2022 F9, should not be interpreted as substantial performance improvements.

For the mutation scale F, the default setting also obtains the best overall ranking, with mean ranks of 1.6207 on CEC2017 and 1.2500 on CEC2022. The corresponding mean ranks of F-low and F-high are 1.9828 and 2.3966 on CEC2017, and 2.0417 and 2.7083 on CEC2022. The influence of F is nevertheless function-dependent. For example, on CEC2022 F6, F-low achieves a mean fitness of 1.8247 × 10^3^ with an SD of 1.2803 × 10^1^, compared with 1.8329 × 10^3^ and 1.4444 × 10^1^ under the default setting. In contrast, F-high produces a mean fitness of 2.6152 × 10^3^ and an SD of 4.2121 × 10^3^ on the same function, indicating substantial variability across runs. Thus, a larger mutation scale does not necessarily improve solution quality and can adversely affect consistency on particular problems.

For the onset of the late mutation stage, the default 70% setting yields the lowest mean rank on both suites. The mean ranks for the 60%, 70%, and 80% settings are 2.5690, 1.4138, and 2.0172 on CEC2017, and 2.6667, 1.0833, and 2.2500 on CEC2022, respectively. Delaying the onset to 80% improves the mean performance on some functions, including CEC2017 F8 and CEC2022 F7, but does not provide a consistent advantage across either suite. The results therefore support retaining the 70% onset as the default setting, while showing that the preferred transition point can vary with the optimization problem.

Overall, the original MSESFOA settings achieve the best mean rank within all three parameter groups on both benchmark suites. This provides empirical support for retaining the default configuration in the reported experiments. Nevertheless, individual functions benefit from alternative settings, and the default configuration does not always produce the smallest SD. The findings therefore indicate a favorable aggregate choice within the tested parameter ranges, rather than a universally optimal configuration. Since the experiments vary one parameter group at a time, interactions among these parameters remain outside the scope of this analysis.

## 5. Engineering Application to Wireless Sensor Network Deployment

To evaluate the applicability of MSESFOA to WSN deployment, this section considers coverage maximization with a fixed number of homogeneous sensors and a fixed sensing radius. A grid-based model is used to evaluate the uncovered ratio in two-dimensional and three-dimensional monitoring regions. The deployment coordinates are optimized within the prescribed region boundaries. MSESFOA is compared with SFOA and the other algorithms in terms of final uncovered ratios, convergence behavior, and spatial deployment patterns. Connectivity, energy consumption, and deployment costs are outside the scope of the present model.

### 5.1. Grid-Based Deployment Model

To describe both planar and spatial deployment scenarios within a single framework, a unified grid-based WSN deployment model with a dimension parameter $\dim\in \left\{2,3\right\}$ is adopted, where dim denotes the dimension of the problem space. Let $A_{k}$ be the number of grid cells along the *k*-th coordinate axis, k=1,2,…,dim. The monitored region is then discretized as shown in Formula (28).

(28) $$g_{\dim}=\prod_{k=1}^{\dim}\left\{1,2,\ldots ,A_{k}\right\},$$

which contains a total of $\prod_{k=1}^{\dim}A_{k}$ grid cells. When dim = 2, the model corresponds to a two-dimensional planar deployment; when dim = 3, it corresponds to a three-dimensional spatial deployment.

The model uses a uniform grid with equal spacing along all coordinate axes. Coordinates and sensing radii are expressed in grid units, with one grid unit representing the distance between adjacent grid locations. Thus, R = 10, R = 12, and R = 25 correspond to sensing radii of 10, 12, and 25 grid spacings, respectively. No specific physical length is assigned to a grid unit in the present experiments. For a physical interpretation, if one grid unit represents a length Δ, the sensing radius becomes RΔ, and the monitored-region dimensions are scaled by the same factor. This uniform scaling preserves the distance-based coverage condition and the calculated coverage ratio.

A total of *S* homogeneous sensor nodes are deployed in the monitored region, each with a sensing radius *R*. The decision vector is defined as $Z={\left[z_{1}^{T},z_{2}^{T},\ldots ,z_{S}^{T}\right]}^{T}$, where $z_{i}=\left(z_{i,1},z_{i,2},\ldots ,z_{i,\dim}\right)$ denotes the coordinates of the *i*-th sensor node. In the discrete grid model, each coordinate is restricted to the corresponding index set.

For any grid cell $v=\left(v_{1},v_{2},\ldots ,v_{\dim}\right)\in g_{\dim}$, it is regarded as covered if there exists at least one sensor node whose Euclidean distance to this cell does not exceed the sensing radius *R*, namely,

(29) $$\min_{i=1,2,\ldots ,S}\left\|z_{i}-v\right\|=\min_{i=1,2,\ldots ,S}\sqrt{\sum_{k=1}^{\dim}{\left(z_{i,k}-v_{k}\right)}^{2}}\leq R.$$

Accordingly, the coverage indicator of cell v under deployment Z is defined as shown in Formula (30).

(30) $$C_{v}\left(Z\right)=\left\{\begin{array}{l} 1,\;\;\;\;\mathrm{if}\min_{i=1,2,\ldots ,S}\left\|z_{i}-v\right\|\leq R\\ 0,\;\;\;\mathrm{otherwise} \end{array}\right..$$

On the basis of the above definition, the overall coverage ratio of the monitored region for deployment Z can be expressed as shown in Formula (31).

(31) $$Cov_{\dim}\left(Z\right)=\frac{\sum_{v\in g_{\dim}}C_{v}\left(Z\right)}{\prod_{k=1}^{\dim}A_{k}}.$$

With a fixed number of sensor nodes, the optimization objective is to maximize the coverage ratio. By defining the uncovered rate as the basis of the fitness function, the problem is equivalently transformed into a minimization form, as shown in Formula (32).

(32) $$f_{\dim}\left(Z\right)=1-Cov_{\dim}\left(Z\right).$$

Consequently, the unified WSN deployment optimization model for both 2D and 3D cases is formulated as follows:

(33) $$\min f_{\dim}\left(Z\right)=1-\frac{\sum_{v\in g_{\dim}}C_{v}\left(Z\right)}{\prod_{k=1}^{\dim}A_{k}},$$

*s.t.*

(34) $$Z=\left\{{\left(z_{i,k}\right)}_{S\times \dim}|1\leq z_{i,k}\leq A_{k},\;\;i=1,2,\ldots ,S,\;\;\;\;k=1,2,\ldots ,\dim\right\}.$$

As an objective function, Formula (33) specifies the optimization objective: with a fixed number of sensors S and sensing radius R, minimizing the coverage deficiency (uncovered ratio) is equivalent to maximizing the overall coverage ratio. Constraint (34) defines the feasible domain of the decision variables: $z_{i,k}$ denotes the discrete grid-index coordinate of the *i*-th sensor in the *k*-th dimension and must lie within the discretized monitored region, ensuring that all the sensors are deployed inside the region.

The present model evaluates coverage under an idealized distance-based sensing assumption, using homogeneous sensors with a fixed sensing radius and a prescribed sensor count. Connectivity, energy consumption, and deployment cost are not included as objectives or constraints. Consequently, a deployment with a high coverage ratio is not necessarily connected or energy-efficient. The experiments assess the ability of MSESFOA to optimize sensor locations for coverage within this model. Applying the method to operational WSN deployment would require additional objectives and constraints reflecting the relevant network requirements.

### 5.2. 2D WSN Deployment

In 2D WSN coverage optimization problems, the monitored 2D region has a size of $L_{x}\times L_{y}$. The experimental settings are characterized by three parameters: monitored region size, number of sensors *S*, and sensing coverage radius *R*, which are summarized in Table 6. In Table 6, there are eight 2D WSN cases, named 2D-P1 to 2D-P8. To ensure a fair comparison, all 16 algorithms are executed under identical simulation settings with a population size of 30 and a maximum of 500 iterations, and each experiment is independently repeated 30 times for each case.

**Figure 5:**
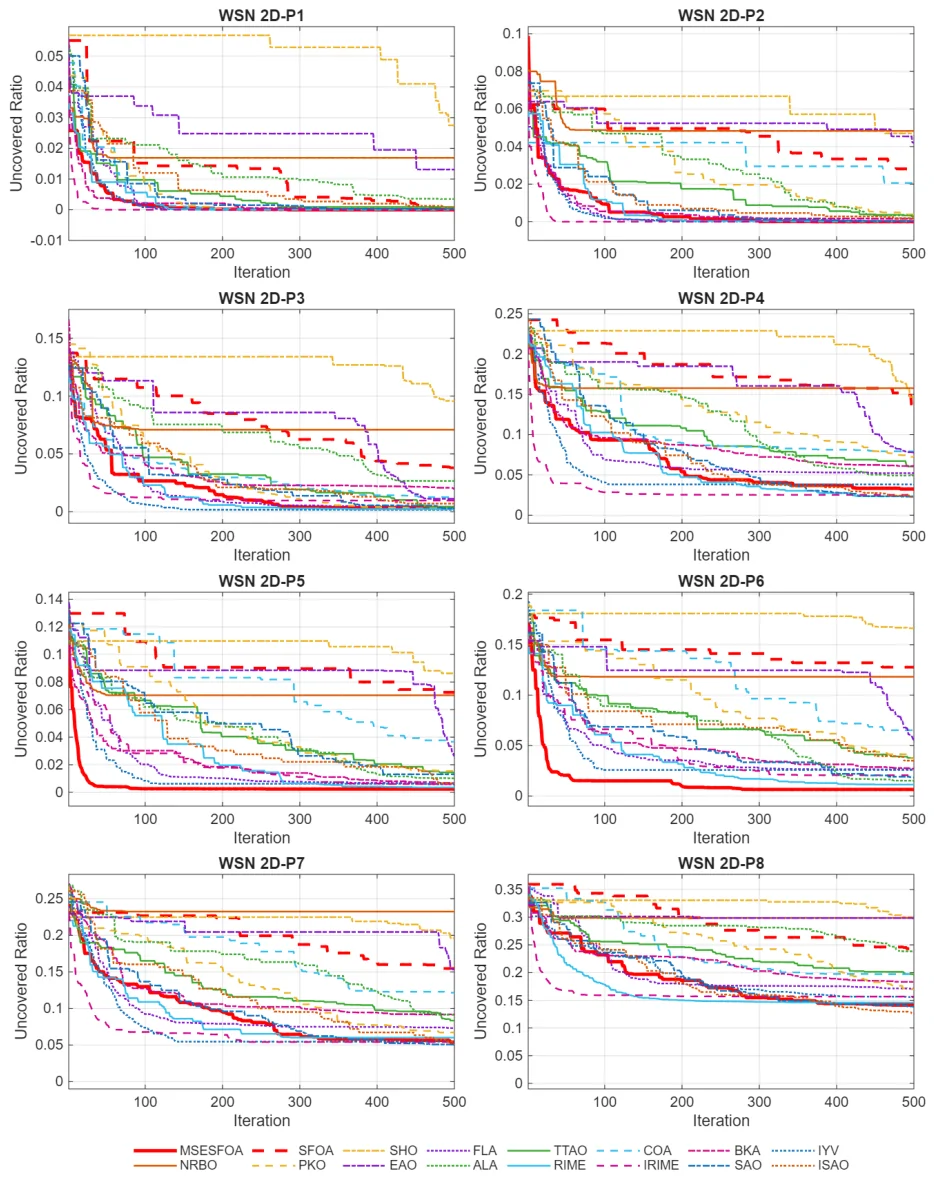
Comparison of the convergence curves of the 16 algorithms for the 2D WSN coverage problem.

**Figure 6:**
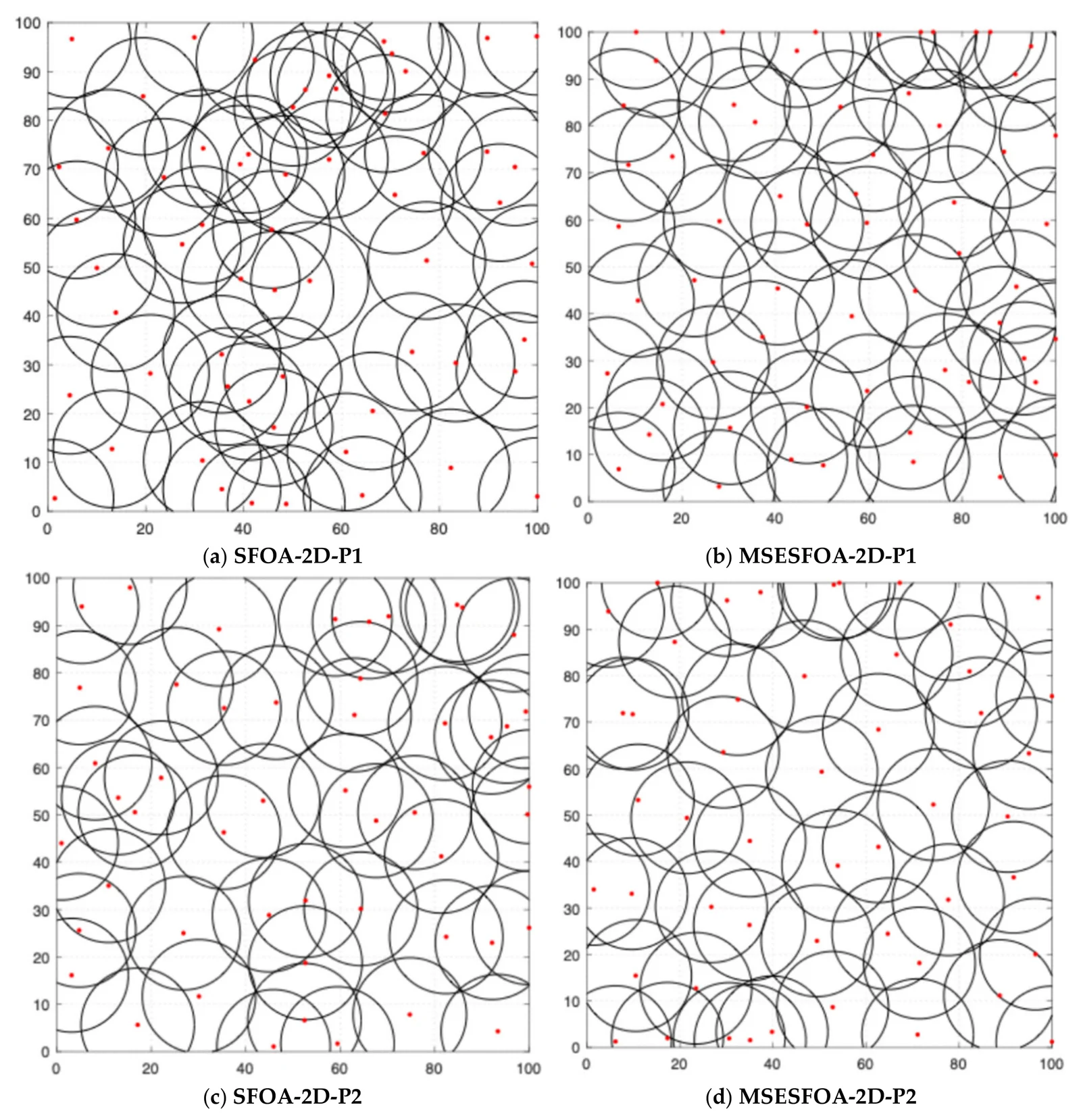

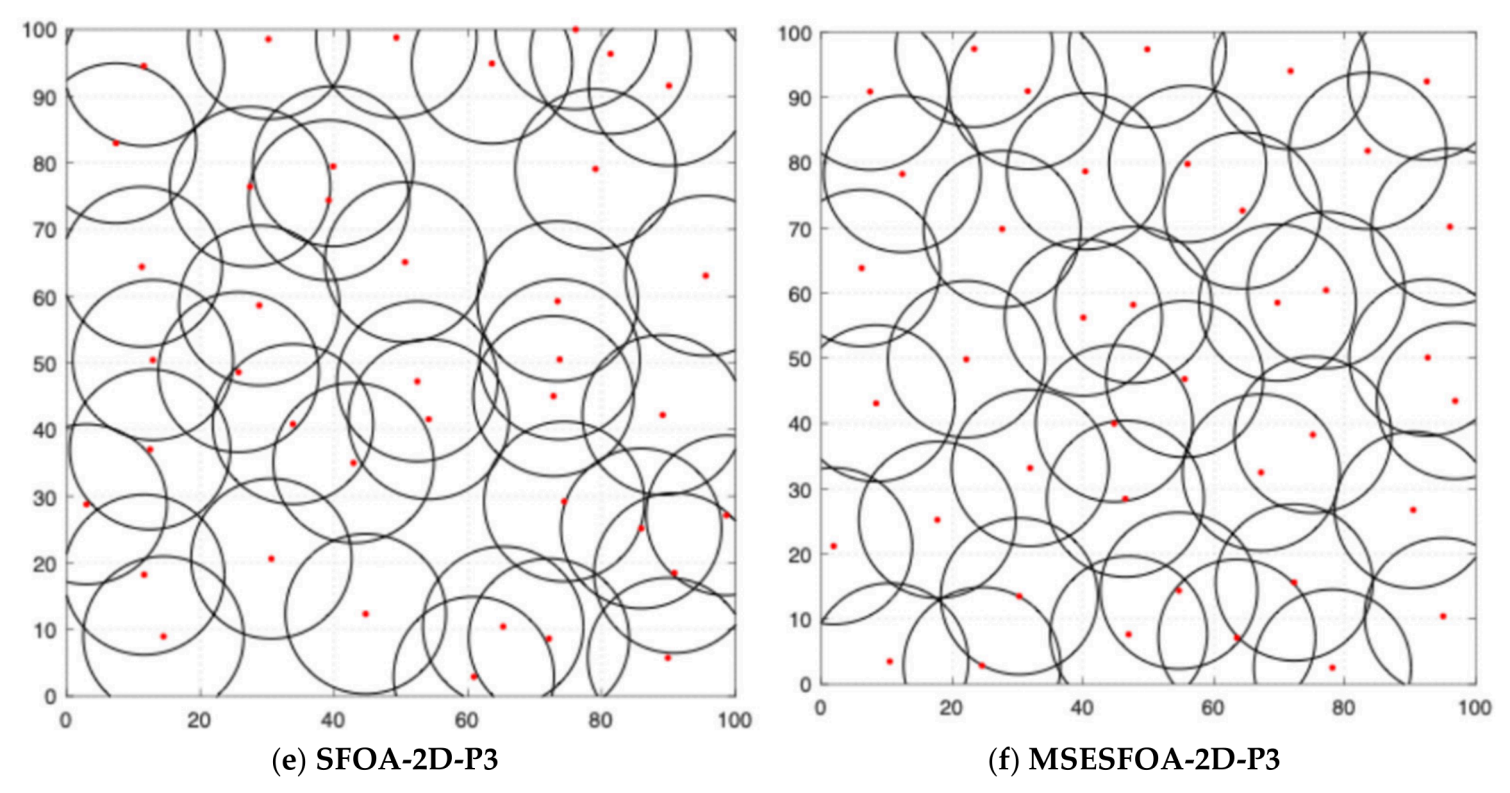
Comparison of the SFOA and MSESFOA for 2D sensor coverage for 2D-P1 to 2D-P3.

**Figure 7:**
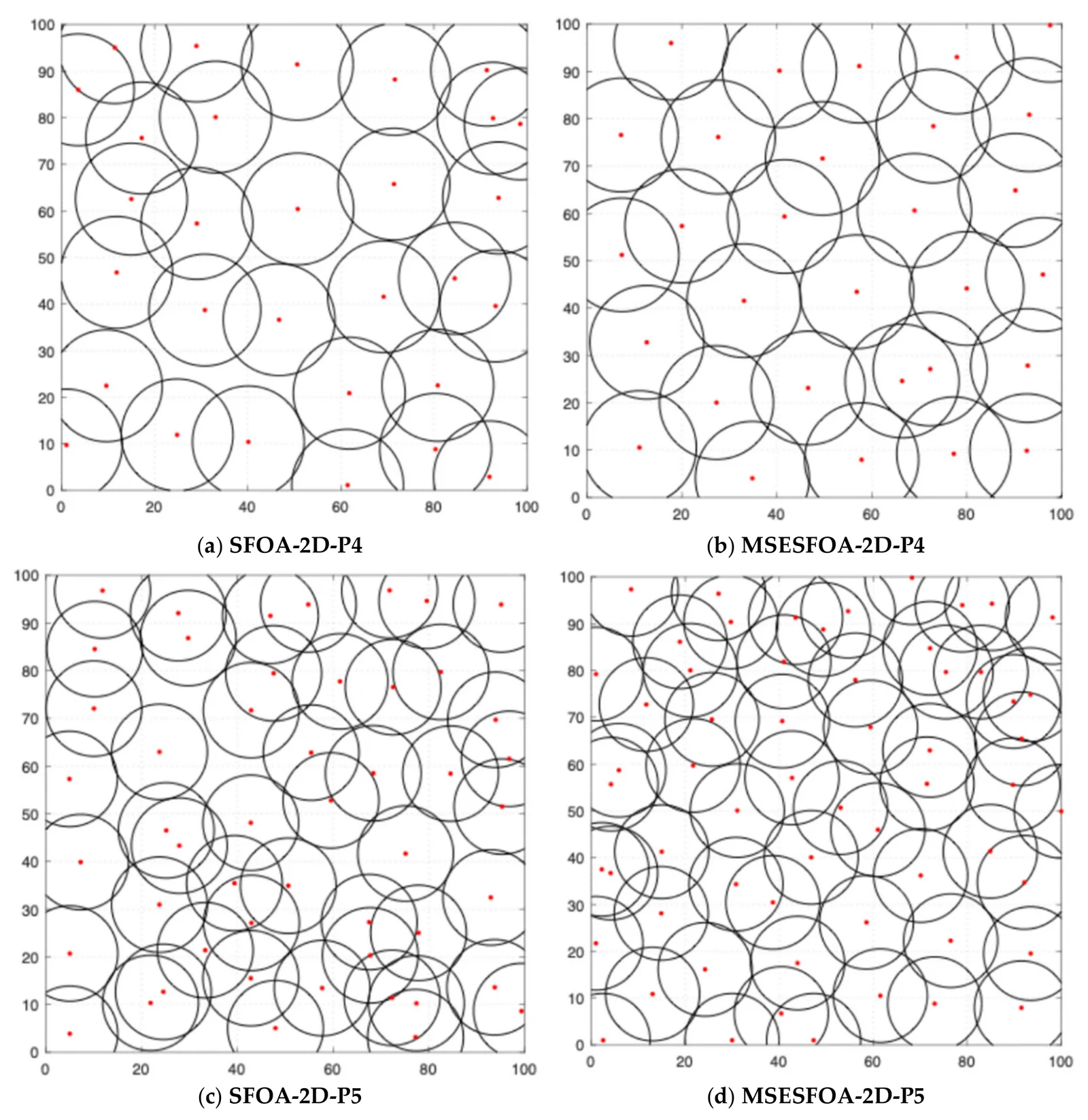

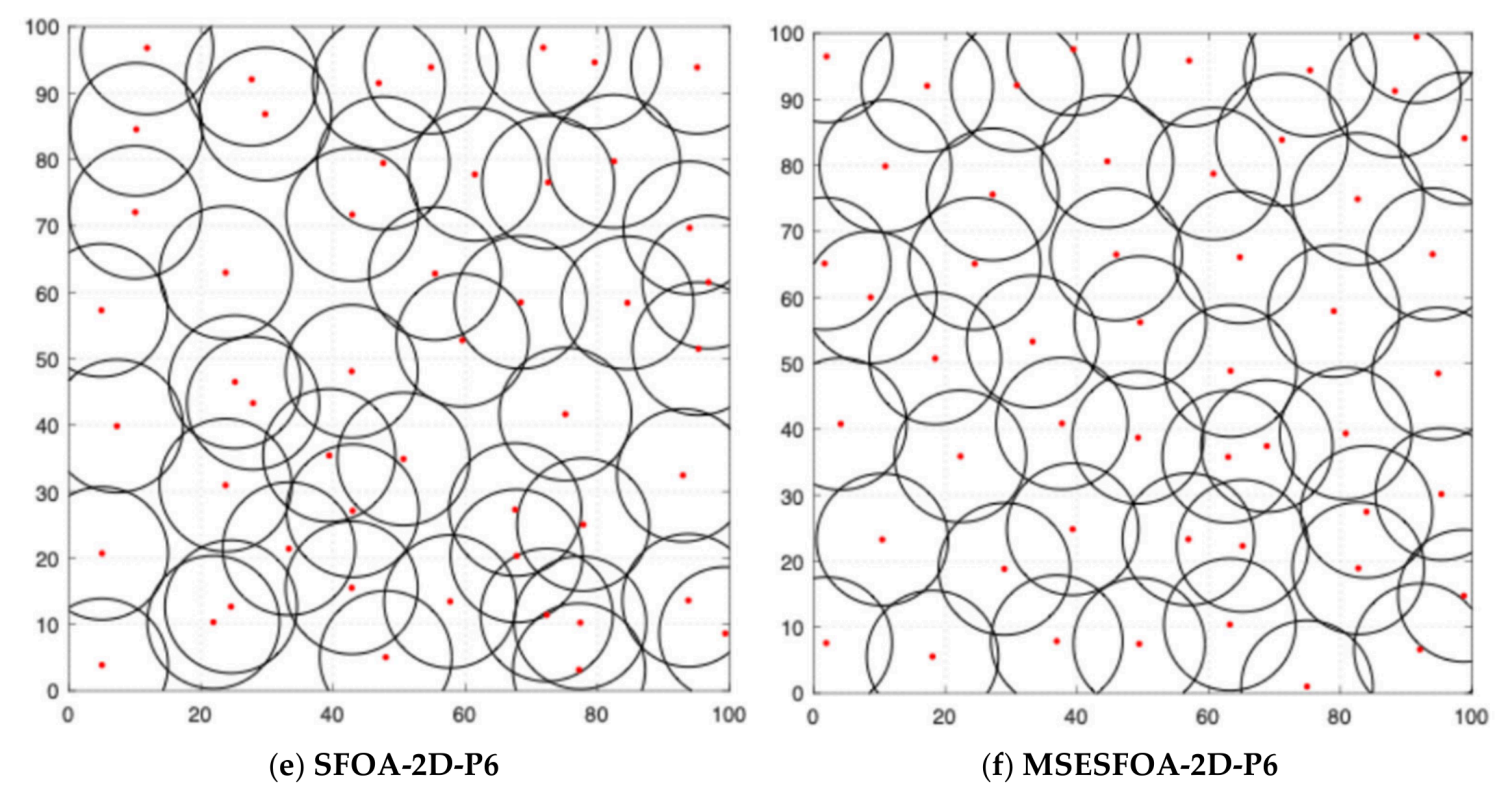
Comparison of the SFOA and MSESFOA for 2D sensor coverage for 2D-P4 to 2D-P6.

**Figure 8:**
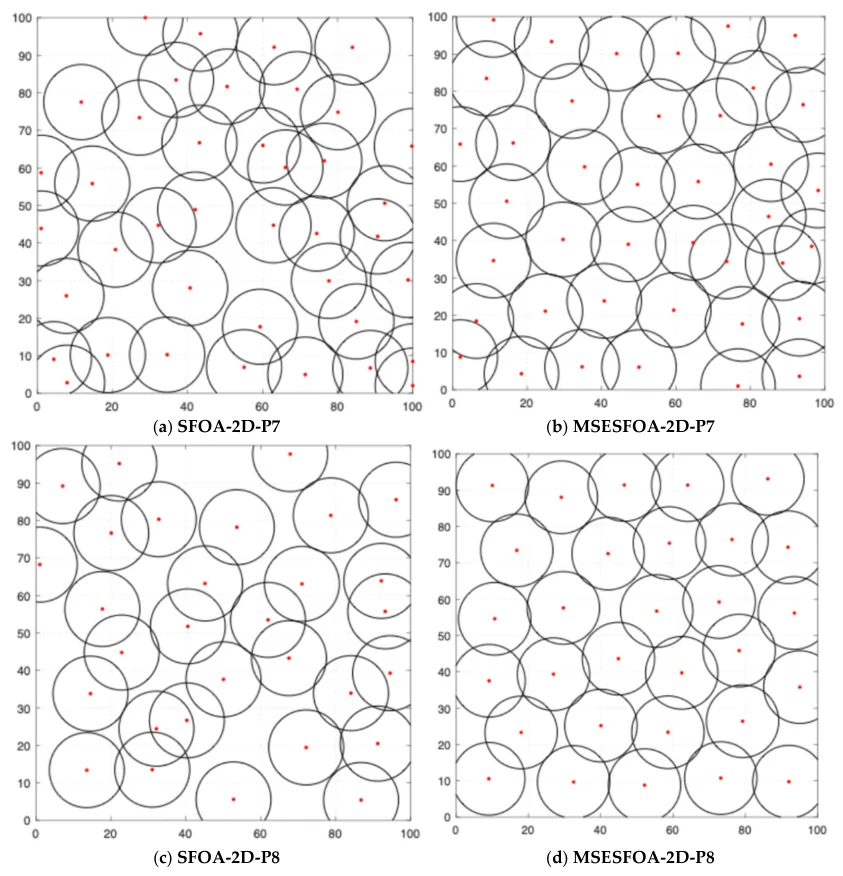
Comparison of the SFOA and MSESFOA for 2D sensor coverage for 2D-P7 to 2D-P8.

The results of the uncovered region ratio are shown from Table A24 and Table A25 in Appendix E, and the convergence curves of the 16 algorithms for the eight 2D WSN cases are shown in Figure 5. Figure 6, Figure 7 and Figure 8 further illustrate the deployment comparison between the original SFOA and the proposed MSESFOA in different 2D cases. The results presented in these tables and figures are explained and analyzed as follows.

From a “grouped + gradient” perspective, the 8 problems can be divided into two radius-based groups, including R = 12 for 2D-P1 to 2D-P4 and R = 10 for 2D-P5 to 2D-P8. The effects of decreasing the number of sensors on problem difficulty and algorithmic performance are then investigated within each group.

For the R = 12 group, in the sensor-sufficient case 2D-P1 (S = 60), the uncovered region ratio of the MSESFOA is equal to 0, with the rank being the first among all 16 algorithms, indicating consistently complete coverage. In contrast, the mean value of the SFOA is $1.2513\times 10^{-2}$, for which the rank is 14, and this value exceeds 1%. The result implies that noticeable uncovered regions exist even when full coverage should be achievable. RIME and BKA also achieve low mean uncovered ratios of 1.0000 × 10^−5^ and 3.6667 × 10^−5^, respectively.

For 2D-P2, the number of sensors decreases to 50. MSESFOA achieves the lowest mean uncovered ratio of 1.8000 × 10^−4^, followed by RIME and SAO, with mean values of 2.3667 × 10^−4^ and 4.0000 × 10^−4^, respectively. In comparison, SFOA produces a mean uncovered ratio of 2.2080 × 10^−2^. These results show that MSESFOA retains the best mean performance in this case and substantially improves upon the original SFOA.

For 2D-P3 and 2D-P4, the number of wireless sensors *S* was further decreased to 40 and 30, respectively. As the number of sensors decreases, the percentage of uncovered regions gradually increases, and the ranking of the proposed MSESFOA decreases to some extent. For these two cases, the MSESFOA ranks fourth and third, respectively. Specifically, for 2D-P3, the algorithms ranked ahead of the MSESFOA are SAO, IVY, and RIME; for 2D-P4, the algorithms ranked ahead of the MSESFOA are IVY and ISAO.

For the R = 10 group, namely, 2D-P5 to 2D-P8, similar to the result for the first four cases, the proposed algorithm displays advantages over the competing methods for 2D-P5 and 2D-P6, for which the number of sensors is relatively large. In these two cases, the MSESFOA remains ranked first. In contrast, for 2D-P7 and 2D-P8, for which fewer sensors are deployed, the algorithm ranks third.

Overall, MSESFOA achieves the best average rank of 2.125 across the eight 2D cases. However, this aggregate result does not imply that it performs best in every individual scenario. It ranks first in 2D-P1, 2D-P2, 2D-P5, and 2D-P6, fourth in 2D-P3, and third in 2D-P4, 2D-P7, and 2D-P8. Thus, its advantage over the competing algorithms is more pronounced in the tested cases with 50 or 60 sensors and decreases in those with 30 or 40 sensors, although it maintains a lower mean uncovered ratio than the original SFOA in all eight cases.

A possible explanation is that reducing the number of sensors changes both the available coverage capacity and the dimensionality of the deployment problem. With more sensors, optimizing their relative positions offers greater opportunities to reduce redundant overlap and close residual coverage gaps. The additional mutation and cooperative recombination mechanisms may help exploit these opportunities. With fewer sensors, the available coverage capacity is more constrained, and the optimization focuses more on distributing limited sensing resources. Meanwhile, the number of decision variables decreases, which may reduce the relative benefit of the additional search mechanisms. This interpretation is consistent with the observed results, but the experiments do not separately establish the effects of coverage capacity and problem dimensionality.

Figure 5 compares the convergence behavior across the eight 2D cases. MSESFOA is not consistently the fastest method during the early iterations, but its curves show continued improvement during later iterations in several cases. Together with the statistics in Table A24 and Table A25, these curves indicate competitive final performance under the tested iteration settings, although MSESFOA does not achieve the lowest mean uncovered ratio in every case.

Figure 6, Figure 7 and Figure 8 compare the deployment layouts obtained by SFOA and MSESFOA across the eight 2D cases. The displayed MSESFOA solutions generally contain fewer or smaller uncovered gaps. MSESFOA achieves complete coverage in all 30 runs of 2D-P1 and a lower mean uncovered ratio than SFOA in all eight cases. Within each sensing-radius group, fewer sensors increase the mean uncovered ratio for both algorithms. However, MSESFOA does not outperform every competitor in every case, as shown in Table A24 and Table A25. The maps illustrate individual solutions, whereas conclusions across independent runs rely on the statistical results.

### 5.3. 3D WSN Deployment

In 3D WSN coverage optimization problems, the monitored 3D region has a size of $L_{x}\times L_{y}\times L_{z}$. The experimental settings are characterized by three parameters: monitored region size, number of sensors *S*, and sensing coverage radius *R*, which are summarized in Table 7. In Table 7, there are four 3D WSN cases, named 3D-P1 to 3D-P4. To ensure a fair comparison, all 16 algorithms are executed under identical simulation settings with a population size of 30 and a maximum of 500 iterations, and each experiment is independently repeated 30 times for each case.

**Figure 9:**
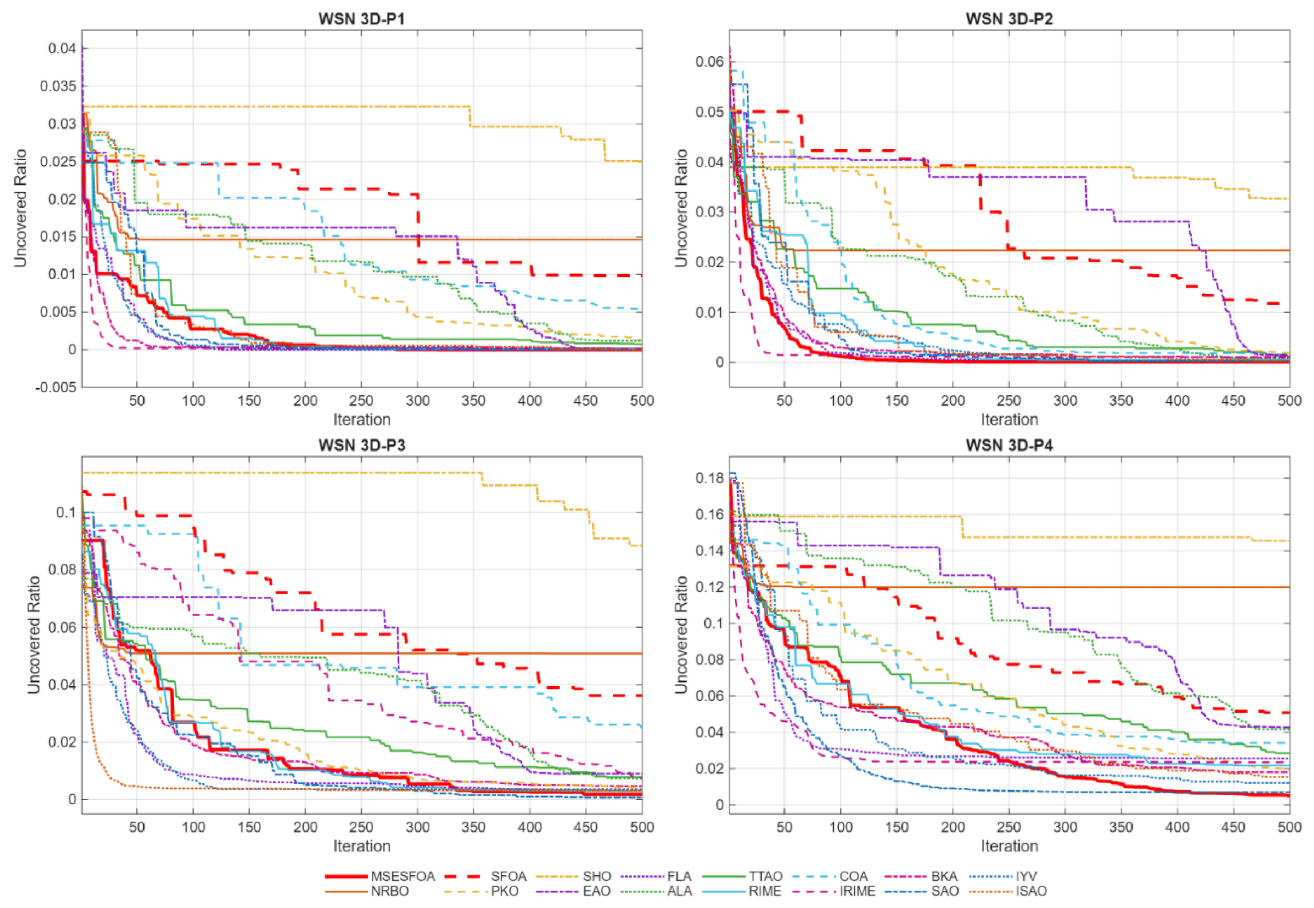
Convergence curve comparison of the 16 algorithms for the 3D WSN coverage problem.

The results of the uncovered region ratio are shown in Table A26 and Table A27 in Appendix F, and the convergence curves of the 16 algorithms for the four 3D WSN cases are shown in Figure 9.

**Figure 10:**
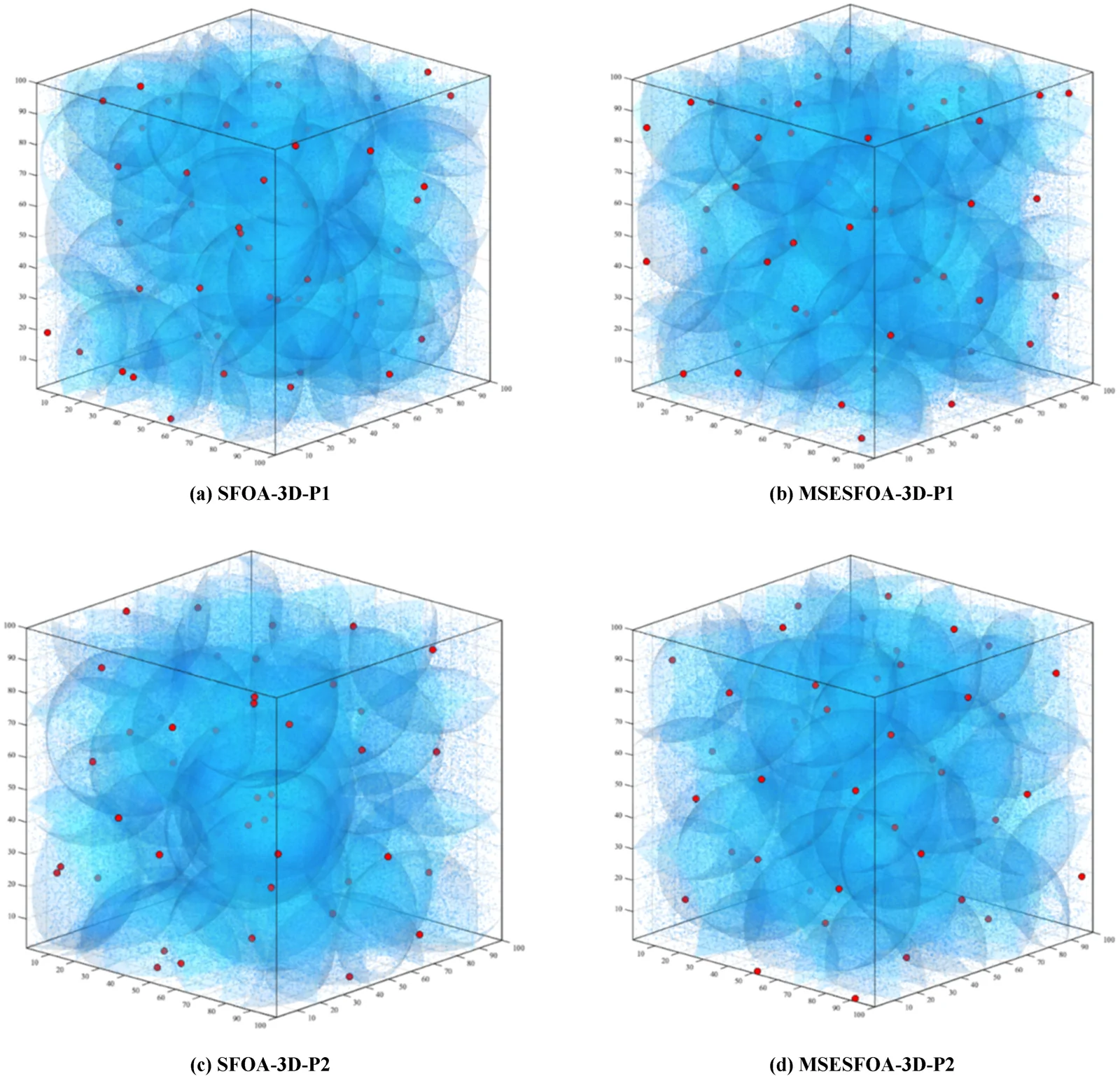
Comparison of the SFOA and MSESFOA for 3D sensor coverage in 3D-P1 and 3D-P2.

**Figure 11:**
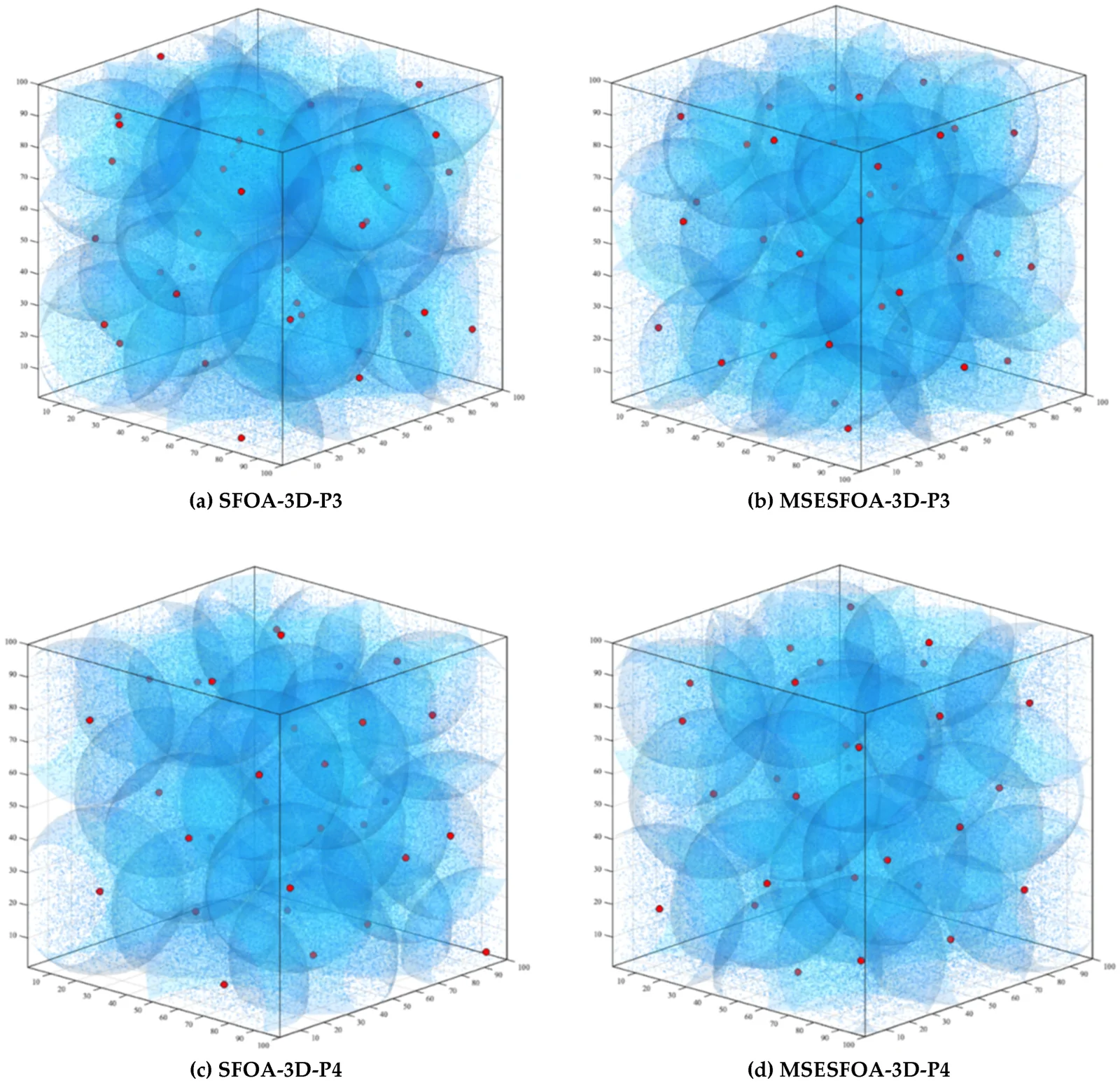
Comparison of the SFOA and MSESFOA for 3D sensor coverage in 3D-P3 and 3D-P4.

Figure 10 and Figure 11 further compare the deployment of the original SFOA and the proposed MSESFOA under different 3D cases. The results presented in these tables and figures are explained and analyzed as follows.

As shown in Table A26 and Table A27, the MSESFOA ranks first for both 3D-P1 and 3D-P2, and it remains consistently competitive under sensor-scarce settings, ranking second for 3D-P3 and 3D-P4. Consequently, it achieves a mean rank of 1.5 and an overall final rank of 1st. In contrast, the original SFOA is ranked 14th or 15th across the four 3D cases, with a mean rank of 14.25, indicating a substantial performance gap.

Moreover, the data in Table A27 suggest that several competitors, such as SAO and ISAO, perform well in these 3D cases. Nevertheless, their results for 2D WSN problems are generally inferior to those of the MSESFOA. This cross-dimensional comparison implies that the MSESFOA provides a more consistently strong performance profile, highlighting its overall competitiveness across heterogeneous WSN coverage scenarios.

The iteration curves in Figure 9 show a trend consistent with that in Figure 5. Although the MSESFOA is not the fastest in the early iteration steps, it typically achieves steadier improvements in the mid-to-late stages, resulting in superior final solution quality. This finding indicates that the proposed method maintains effective search progress throughout the optimization process.

As can be observed from the comparison of the spatial coverage maps of the SFOA and MSESFOA results shown in Figure 10 and Figure 11, the MSESFOA generally yields a more desirable 3D deployment pattern with a more uniform spatial distribution, less local clustering and redundant overlap, and, thus, a larger effective covered volume with fewer uncovered “cavities”. In comparison, the SFOA is more prone to generating locally concentrated layouts, which results in overly dense coverage in some subregions while other subregions are insufficiently covered.

## 6. Conclusions

In this paper, the MSESFOA is proposed to address the limitations of the original SFOA, particularly in terms of exploration strategy flexibility, population diversity preservation, and information fusion. Adaptive exploration regulation, stage-based combined mutation, and multi-arm cooperative recombination jointly regulate strategy selection, introduce stage-dependent perturbations, and facilitate cooperative information exchange.

Extensive experiments based on the CEC 2017 and CEC 2022 benchmark functions, where the MSESFOA is compared with the SFOA and 14 competitive algorithms, demonstrate that the MSESFOA achieves the best overall mean rank on both benchmark suites; for instance, it achieves the best overall results for CEC 2022, with a mean rank of 1.50.

Moreover, a unified grid-based WSN coverage optimization model is established and extended to 3D scenarios, with the deployment task formulated as minimizing the uncovered ratio. MSESFOA achieves the best average ranks of 2.125 and 1.5 across the tested 2D and 3D cases, respectively. Nevertheless, its performance varies across individual scenarios: it ranks fourth in 2D-P3, third in 2D-P4, 2D-P7, and 2D-P8, and second in 3D-P3 and 3D-P4. These results support its overall competitiveness for coverage optimization under the tested homogeneous-sensor settings, although its advantage varies across individual scenarios.

Future work may proceed in two directions. First, the early-stage convergence efficiency can be further improved while preserving the strong mid-to-late exploitation capability and overall solution stability. Second, the model can be extended toward more realistic WSN deployment settings by incorporating practical constraints and objectives, such as connectivity, energy balancing, obstacles/no-go zones, and heterogeneous sensors.

## Figures and Tables

**Table 1:** Module-level computational complexity of SFOA and MSESFOA.

Algorithms	SFOA	MSESFOA
Initialization	$O\left(N\cdot D\right)$	$O\left(N\cdot D\right)$
Exploration or Exploitation	$O\left(N\cdot D\right)$	$O\left(N\cdot D\right)$
Stage-based combined mutation	—	$O\left(M_{T}\cdot D\right)$
Multi-arm Cooperative Recombination	—	$O\left(N\cdot D\right)$
Total	$O\left[\left(T_{\max}+1\right)\cdot N\cdot D\right]$	$O\left[\left(\left(2+\bar{\rho }\right)\cdot T_{\max}+1\right)\cdot N\cdot D\right]$

**Table 2:** Details of CEC 2017.

Type	No.	Functions	Best Values
Unimodal functions	F1	Shifted and rotated bent cigar function	100
	F3	Shifted and rotated Zakharov function	300
Simple multimodal functions	F4	Shifted and rotated Rosenbrock’s function	400
	F5	Shifted and rotated Rastrigin’s function	500
	F6	Shifted and rotated expanded Scaffer’s F6 function	600
	F7	Shifted and rotated Lunacek Bi-Rastrigin’s function	700
	F8	Shifted and rotated noncontinuous Rastrigin’s function	800
	F9	Shifted and rotated Levy function	900
	F10	Shifted and rotated Schwefel’s function	1000
Hybrid functions	F11	Hybrid function 1 (N = 3)	1100
	F12	Hybrid function 2 (N = 3)	1200
	F13	Hybrid function 3 (N = 3)	1300
	F14	Hybrid function 4 (N = 4)	1400
	F15	Hybrid function 5 (N = 4)	1500
	F16	Hybrid function 6 (N = 4)	1600
	F17	Hybrid function 7 (N = 5)	1700
	F18	Hybrid function 8 (N = 5)	1800
	F19	Hybrid function 9 (N = 5)	1900
	F20	Hybrid function 10 (N = 6)	2000
Composition functions	F21	Composition function 1 (N = 3)	2100
	F22	Composition function 2 (N = 3)	2200
	F23	Composition function 3 (N = 3)	2300
	F24	Composition function 4 (N = 3)	2400
	F25	Composition function 5 (N = 3)	2500
	F26	Composition function 6 (N = 3)	2600
	F27	Composition function 7 (N = 3)	2700
	F28	Composition function 8 (N = 3)	2800
	F29	Composition function 9 (N = 3)	2900
	F30	Composition function 10 (N = 3)	3000

**Table 3:** Details of CEC 2022.

Type	No.	Functions	Best Values
Unimodal functions	F1	Shifted and full rotated Zakharov function	300
Basic functions	F2	Shifted and full rotated Rosenbrock’s function	400
	F3	Shifted and full rotated expanded Scaffer’s F6 function	600
	F4	Shifted and full rotated noncontinuous Rastrigin’s function	800
	F5	Shifted and full rotated Levy function	900
Hybrid functions	F6	Hybrid function 1 (N = 3)	1800
	F7	Hybrid function 2 (N = 6)	2000
	F8	Hybrid function 3 (N = 5)	2200
Composition functions	F9	Composition function 1 (N = 5)	2300
	F10	Composition function 2 (N = 4)	2400
	F11	Composition function 3 (N = 6)	2600
	F12	Composition function 4 (N = 6)	2700

**Table 4:** Wilcoxon signed-rank test results with Holm correction on CEC2017.

MSESFOA vs.	R+	R−	Raw *p*	Holm-Adjusted *p*	Significant (0.05)
SFOA	435	0	3.7253 × 10^−9^	5.5879 × 10^−8^	YES
SHO	435	0	3.7253 × 10^−9^	5.5879 × 10^−8^	YES
FLA	435	0	3.7253 × 10^−9^	5.5879 × 10^−8^	YES
TTAO	435	0	3.7253 × 10^−9^	5.5879 × 10^−8^	YES
COA	435	0	3.7253 × 10^−9^	5.5879 × 10^−8^	YES
BKA	435	0	3.7253 × 10^−9^	5.5879 × 10^−8^	YES
IVY	435	0	3.7253 × 10^−9^	5.5879 × 10^−8^	YES
NRBO	435	0	3.7253 × 10^−9^	5.5879 × 10^−8^	YES
PKO	431	4	2.6077 × 10^−8^	1.0431 × 10^−7^	YES
EAO	388	47	7.7311 × 10^−5^	7.7311 × 10^−5^	YES
ALA	435	0	3.7253 × 10^−9^	5.5879 × 10^−8^	YES
RIME	435	0	3.7253 × 10^−9^	5.5879 × 10^−8^	YES
IRIME	435	0	3.7253 × 10^−9^	5.5879 × 10^−8^	YES
SAO	423	12	2.6077 × 10^−7^	7.8231 × 10^−7^	YES
ISAO	422	13	3.2783 × 10^−7^	7.8231 × 10^−7^	YES

**Note:** Each mean is calculated from 30 independent runs. Differences are defined as the competing algorithm’s mean fitness minus that of MSESFOA; positive differences favor MSESFOA. Exact zero differences are excluded, and tied absolute differences receive average ranks. Holm correction is applied to the 15 comparisons within each benchmark suite. Significance is determined using the adjusted *p*-values at 0.05.

**Table 5:** Wilcoxon signed-rank test results with Holm correction on CEC2022.

MSESFOA vs.	R+	R−	Raw *p*	Holm-Adjusted *p*	Significant (0.05)
SFOA	78	0	4.8828 × 10^−4^	7.3242 × 10^−3^	Yes
SHO	78	0	4.8828 × 10^−4^	7.3242 × 10^−3^	Yes
FLA	62	4	6.8359 × 10^−3^	2.7344 × 10^−2^	Yes
TTAO	72	6	6.8359 × 10^−3^	2.7344 × 10^−2^	Yes
COA	78	0	4.8828 × 10^−4^	7.3242 × 10^−3^	Yes
BKA	78	0	4.8828 × 10^−4^	7.3242 × 10^−3^	Yes
IVY	78	0	4.8828 × 10^−4^	7.3242 × 10^−3^	Yes
NRBO	78	0	4.8828 × 10^−4^	7.3242 × 10^−3^	Yes
PKO	71	7	9.2773 × 10^−3^	2.7344 × 10^−2^	Yes
EAO	53	13	8.3008 × 10^−2^	8.3008 × 10^−2^	No
ALA	78	0	4.8828 × 10^−4^	7.3242 × 10^−3^	Yes
RIME	78	0	4.8828 × 10^−4^	7.3242 × 10^−3^	Yes
IRIME	78	0	4.8828 × 10^−4^	7.3242 × 10^−3^	Yes
SAO	65	1	1.9531 × 10^−3^	1.1719 × 10^−2^	Yes
ISAO	63	3	4.8828 × 10^−3^	2.4414 × 10^−2^	Yes

**Note:** Each mean is calculated from 30 independent runs. Differences are defined as the competing algorithm’s mean fitness minus that of MSESFOA; positive differences favor MSESFOA. Exact zero differences are excluded, and tied absolute differences receive average ranks. Holm correction is applied to the 15 comparisons within each benchmark suite. Significance is determined using the adjusted *p*-values at 0.05.

**Table 6:** Parameter settings for 2D WSN deployment problems (Note: Monitored-region dimensions and sensing radii are expressed in grid units, as defined in Section 5.1).

Problems	Size of Region	S	R
2D-P1	100 × 100	60	12
2D-P2	100 × 100	50	12
2D-P3	100 × 100	40	12
2D-P4	100 × 100	30	12
2D-P5	100 × 100	60	10
2D-P6	100 × 100	50	10
2D-P7	100 × 100	40	10
2D-P8	100 × 100	30	10

**Table 7:** Parameter settings for 3D WSN deployment cases (Note: Monitored-region dimensions and sensing radii are expressed in grid units, as defined in Section 5.1).

Problems	Size of Region	S	R
3D-P1	100 × 100 × 100	60	25
3D-P2	100 × 100 × 100	50	25
3D-P3	100 × 100 × 100	40	25
3D-P4	100 × 100 × 100	30	25

## Data Availability

The data supporting the findings of this study are available within the article.

## Appendix A. Statistical Results of the 16 Algorithms Based on the CEC 2017 Benchmark Functions

**Table A1:** Statistical results of the 16 algorithms based on the CEC 2017 benchmark functions (F1 to F11 for the first eight algorithms).

Functions	Index	MSESFOA	SFOA	SHO	FLA	TTAO	COA	BKA	IVY
F1	Mean	1.0000 × 10^2^	7.9185 × 10^3^	1.2527 × 10^10^	3.5899 × 10^3^	2.2504 × 10^3^	1.2762 × 10^4^	2.6049 × 10^9^	8.4787 × 10^10^
	SD	2.8700 × 10^−7^	6.9407 × 10^3^	4.2962 × 10^9^	3.2564 × 10^3^	2.0154 × 10^3^	1.0980 × 10^4^	6.6163 × 10^9^	6.1008 × 10^3^
	Best	1.0000 × 10^2^	7.0592 × 10^2^	7.4431 × 10^9^	1.1677 × 10^2^	1.0340 × 10^2^	1.2021 × 10^3^	1.4570 × 10^6^	8.4787 × 10^10^
	Worst	1.0000 × 10^2^	2.2361 × 10^4^	2.3256 × 10^10^	1.0416 × 10^4^	7.3911 × 10^3^	4.5253 × 10^4^	2.4579 × 10^10^	8.4787 × 10^10^
	Rank	1	9	15	6	5	11	13	16
F3	Mean	3.0000 × 10^2^	3.0012 × 10^2^	4.9342 × 10^4^	3.0000 × 10^2^	3.1466 × 10^2^	2.4694 × 10^4^	5.4994 × 10^3^	1.0884 × 10^9^
	SD	4.8754 × 10^−11^	1.1044 × 10^−1^	8.5160 × 10^3^	4.9668 × 10^−6^	1.0294 × 10^1^	8.2274 × 10^3^	1.2918 × 10^4^	9.6998 × 10^−7^
	Best	3.0000 × 10^2^	3.0001 × 10^2^	3.2002 × 10^4^	3.0000 × 10^2^	3.0388 × 10^2^	5.9145 × 10^3^	3.6485 × 10^2^	1.0884 × 10^9^
	Worst	3.0000 × 10^2^	3.0052 × 10^2^	6.5078 × 10^4^	3.0000 × 10^2^	3.4064 × 10^2^	4.0889 × 10^4^	4.6772 × 10^4^	1.0884 × 10^9^
	Rank	1	6	15	2	8	11	9	16
F4	Mean	4.3116 × 10^2^	4.7163 × 10^2^	1.6657 × 10^3^	4.3153 × 10^2^	4.7894 × 10^2^	4.8908 × 10^2^	1.1620 × 10^3^	3.5319 × 10^4^
	SD	3.0968 × 10^1^	2.1732 × 10^1^	9.0678 × 10^2^	2.9413 × 10^1^	2.5684 × 10^1^	2.7313 × 10^1^	1.5962 × 10^3^	0.0000 × 10^0^
	Best	4.0000 × 10^2^	4.0057 × 10^2^	7.4884 × 10^2^	4.0000 × 10^2^	4.0412 × 10^2^	4.0106 × 10^2^	4.4847 × 10^2^	3.5319 × 10^4^
	Worst	4.6412 × 10^2^	4.9023 × 10^2^	4.8423 × 10^3^	4.5856 × 10^2^	5.1289 × 10^2^	5.3604 × 10^2^	7.4148 × 10^3^	3.5319 × 10^4^
	Rank	1	7	15	2	8	9	14	16
F5	Mean	5.3727 × 10^2^	6.2533 × 10^2^	6.8744 × 10^2^	6.1110 × 10^2^	6.1518 × 10^2^	7.0727 × 10^2^	7.0577 × 10^2^	1.1260 × 10^3^
	SD	2.0379 × 10^1^	3.2945 × 10^1^	3.2719 × 10^1^	3.8289 × 10^1^	1.9864 × 10^1^	6.5893 × 10^1^	3.8707 × 10^1^	9.2504 × 10^−13^
	Best	5.0149 × 10^2^	5.6651 × 10^2^	6.4249 × 10^2^	5.5831 × 10^2^	5.7074 × 10^2^	5.6698 × 10^2^	6.3987 × 10^2^	1.1260 × 10^3^
	Worst	5.7153 × 10^2^	6.8876 × 10^2^	7.8020 × 10^2^	7.1619 × 10^2^	6.6091 × 10^2^	8.0246 × 10^2^	8.2070 × 10^2^	1.1260 × 10^3^
	Rank	1	10	12	8	9	14	13	16
F6	Mean	6.0000 × 10^2^	6.0491 × 10^2^	6.2691 × 10^2^	6.1683 × 10^2^	6.1399 × 10^2^	6.4043 × 10^2^	6.5094 × 10^2^	7.4788 × 10^2^
	SD	5.1386 × 10^−12^	2.4464 × 10^0^	6.8118 × 10^0^	8.1309 × 10^0^	9.4192 × 10^0^	1.9962 × 10^1^	6.2601 × 10^0^	0.0000 × 10^0^
	Best	6.0000 × 10^2^	6.0171 × 10^2^	6.1502 × 10^2^	6.0578 × 10^2^	6.0359 × 10^2^	6.0913 × 10^2^	6.4037 × 10^2^	7.4788 × 10^2^
	Worst	6.0000 × 10^2^	6.1137 × 10^2^	6.3854 × 10^2^	6.2844 × 10^2^	6.3623 × 10^2^	6.6352 × 10^2^	6.6123 × 10^2^	7.4788 × 10^2^
	Rank	1	8	12	11	10	13	14	16
F7	Mean	7.7437 × 10^2^	9.3848 × 10^2^	1.0218 × 10^3^	8.5957 × 10^2^	7.9965 × 10^2^	1.1112 × 10^3^	1.1547 × 10^3^	1.6605 × 10^3^
	SD	2.2429 × 10^1^	2.8192 × 10^1^	6.2909 × 10^1^	4.5366 × 10^1^	1.5551 × 10^1^	1.2373 × 10^2^	7.8692 × 10^1^	9.2504 × 10^−13^
	Best	7.2102 × 10^2^	8.7694 × 10^2^	9.4753 × 10^2^	8.0503 × 10^2^	7.7685 × 10^2^	8.9551 × 10^2^	1.0200 × 10^3^	1.6605 × 10^3^
	Worst	7.9550 × 10^2^	9.7780 × 10^2^	1.1633 × 10^3^	9.9088 × 10^2^	8.2557 × 10^2^	1.3236 × 10^3^	1.3320 × 10^3^	1.6605 × 10^3^
	Rank	4	11	12	9	7	13	15	16
F8	Mean	8.6079 × 10^2^	9.4793 × 10^2^	9.5274 × 10^2^	8.9207 × 10^2^	8.9123 × 10^2^	9.6692 × 10^2^	9.5269 × 10^2^	1.3210 × 10^3^
	SD	1.4003 × 10^1^	5.2149 × 10^1^	2.3172 × 10^1^	2.1234 × 10^1^	1.2269 × 10^1^	2.6882 × 10^1^	2.6921 × 10^1^	1.1563 × 10^−12^
	Best	8.3651 × 10^2^	8.4562 × 10^2^	9.0722 × 10^2^	8.6368 × 10^2^	8.6204 × 10^2^	9.2138 × 10^2^	9.1952 × 10^2^	1.3210 × 10^3^
	Worst	8.8656 × 10^2^	1.0360 × 10^3^	9.8900 × 10^2^	9.5836 × 10^2^	9.1431 × 10^2^	1.0149 × 10^3^	1.0127 × 10^3^	1.3210 × 10^3^
	Rank	5	10	12	9	8	13	11	16
F9	Mean	9.0000 × 10^2^	1.4601 × 10^3^	4.5338 × 10^3^	1.8271 × 10^3^	1.0145 × 10^3^	3.4047 × 10^3^	4.1988 × 10^3^	3.4486 × 10^4^
	SD	0.0000 × 10^0^	7.0916 × 10^2^	5.1083 × 10^2^	8.3432 × 10^2^	1.0186 × 10^2^	1.6351 × 10^3^	6.2701 × 10^2^	2.2201 × 10^−11^
	Best	9.0000 × 10^2^	9.3309 × 10^2^	3.4274 × 10^3^	9.4380 × 10^2^	9.1280 × 10^2^	1.2627 × 10^3^	3.2053 × 10^3^	3.4486 × 10^4^
	Worst	9.0000 × 10^2^	3.6862 × 10^3^	5.5495 × 10^3^	3.9034 × 10^3^	1.3036 × 10^3^	5.9288 × 10^3^	5.8501 × 10^3^	3.4486 × 10^4^
	Rank	1	9	13	10	8	11	12	16
F10	Mean	3.2489 × 10^3^	7.0490 × 10^3^	5.0927 × 10^3^	4.7279 × 10^3^	4.4762 × 10^3^	5.1213 × 10^3^	4.7734 × 10^3^	1.1296 × 10^4^
	SD	4.2993 × 10^2^	6.9067 × 10^2^	7.4721 × 10^2^	8.8974 × 10^2^	3.5508 × 10^2^	7.6179 × 10^2^	5.7650 × 10^2^	0.0000 × 10^0^
	Best	2.2195 × 10^3^	5.3917 × 10^3^	3.6294 × 10^3^	2.9289 × 10^3^	3.6234 × 10^3^	4.1651 × 10^3^	3.7811 × 10^3^	1.1296 × 10^4^
	Worst	4.1316 × 10^3^	7.9652 × 10^3^	6.3139 × 10^3^	6.9404 × 10^3^	5.4794 × 10^3^	7.5906 × 10^3^	6.6719 × 10^3^	1.1296 × 10^4^
	Rank	1	14	11	9	6	12	10	16
F11	Mean	1.1193 × 10^3^	1.1898 × 10^3^	2.0634 × 10^3^	1.2794 × 10^3^	1.1881 × 10^3^	1.2770 × 10^3^	1.3524 × 10^3^	6.1858 × 10^8^
	SD	1.9067 × 10^1^	2.9827 × 10^1^	5.9750 × 10^2^	6.4207 × 10^1^	2.2687 × 10^1^	6.9936 × 10^1^	2.1814 × 10^2^	0.0000 × 10^0^
	Best	1.1010 × 10^3^	1.1258 × 10^3^	1.3393 × 10^3^	1.1607 × 10^3^	1.1557 × 10^3^	1.1281 × 10^3^	1.1755 × 10^3^	6.1858 × 10^8^
	Worst	1.1908 × 10^3^	1.2625 × 10^3^	4.2195 × 10^3^	1.4206 × 10^3^	1.2306 × 10^3^	1.4073 × 10^3^	2.0463 × 10^3^	6.1858 × 10^8^
	Rank	1	8	15	11	7	10	13	16

**Table A2:** Statistical results of the 16 algorithms based on the CEC 2017 benchmark functions (F11 to F20 for the first eight algorithms).

Functions	Index	MSESFOA	SFOA	SHO	FLA	TTAO	COA	BKA	IVY
F11	Mean	1.1193 × 10^3^	1.1898 × 10^3^	2.0634 × 10^3^	1.2794 × 10^3^	1.1881 × 10^3^	1.2770 × 10^3^	1.3524 × 10^3^	6.1858 × 10^8^
	SD	1.9067 × 10^1^	2.9827 × 10^1^	5.9750 × 10^2^	6.4207 × 10^1^	2.2687 × 10^1^	6.9936 × 10^1^	2.1814 × 10^2^	0.0000 × 10^0^
	Best	1.1010 × 10^3^	1.1258 × 10^3^	1.3393 × 10^3^	1.1607 × 10^3^	1.1557 × 10^3^	1.1281 × 10^3^	1.1755 × 10^3^	6.1858 × 10^8^
	Worst	1.1908 × 10^3^	1.2625 × 10^3^	4.2195 × 10^3^	1.4206 × 10^3^	1.2306 × 10^3^	1.4073 × 10^3^	2.0463 × 10^3^	6.1858 × 10^8^
	Rank	1	8	15	11	7	10	13	16
F12	Mean	1.6474 × 10^3^	2.3425 × 10^4^	4.2256 × 10^8^	2.0242 × 10^4^	3.0939 × 10^4^	1.1753 × 10^6^	9.2476 × 10^7^	2.9488 × 10^10^
	SD	3.3413 × 10^2^	2.4467 × 10^4^	5.8788 × 10^8^	1.6870 × 10^4^	1.4676 × 10^4^	1.0697 × 10^6^	4.6676 × 10^8^	3.4165 × 10^4^
	Best	1.2445 × 10^3^	2.4868 × 10^3^	1.0168 × 10^7^	6.0202 × 10^3^	8.6614 × 10^3^	8.9639 × 10^4^	4.2676 × 10^5^	2.9488 × 10^10^
	Worst	2.9117 × 10^3^	1.0619 × 10^5^	2.7398 × 10^9^	5.7105 × 10^4^	6.1753 × 10^4^	3.3531 × 10^6^	2.5604 × 10^9^	2.9488 × 10^10^
	Rank	1	5	14	4	6	10	13	16
F13	Mean	1.3369 × 10^3^	2.1416 × 10^3^	2.1098 × 10^7^	3.3285 × 10^4^	9.8106 × 10^3^	3.0719 × 10^4^	3.6188 × 10^5^	4.4188 × 10^10^
	SD	1.2308 × 10^1^	9.4720 × 10^2^	5.8311 × 10^7^	2.4223 × 10^4^	9.4729 × 10^3^	1.9389 × 10^4^	1.4842 × 10^6^	7.7598 × 10^−6^
	Best	1.3199 × 10^3^	1.4160 × 10^3^	1.9875 × 10^4^	2.5253 × 10^3^	1.6409 × 10^3^	6.1442 × 10^3^	3.9425 × 10^4^	4.4188 × 10^10^
	Worst	1.3683 × 10^3^	4.7927 × 10^3^	2.7490 × 10^8^	6.4166 × 10^4^	5.0489 × 10^4^	7.3974 × 10^4^	8.2149 × 10^6^	4.4188 × 10^10^
	Rank	1	4	15	12	5	11	13	16
F14	Mean	1.4311 × 10^3^	1.4650 × 10^3^	2.3242 × 10^5^	1.9603 × 10^3^	3.6451 × 10^3^	2.2042 × 10^4^	1.6724 × 10^3^	1.2512 × 10^9^
	SD	5.0149 × 10^0^	2.3286 × 10^1^	2.5275 × 10^5^	7.2654 × 10^2^	1.8430 × 10^3^	2.3049 × 10^4^	6.9015 × 10^1^	7.2748 × 10^−7^
	Best	1.4094 × 10^3^	1.4407 × 10^3^	1.0375 × 10^4^	1.5666 × 10^3^	1.8844 × 10^3^	2.4612 × 10^3^	1.5544 × 10^3^	1.2512 × 10^9^
	Worst	1.4425 × 10^3^	1.5370 × 10^3^	1.0934 × 10^6^	5.6839 × 10^3^	8.2507 × 10^3^	1.0122 × 10^5^	1.8846 × 10^3^	1.2512 × 10^9^
	Rank	1	4	14	6	7	13	5	15
F15	Mean	1.5127 × 10^3^	1.6391 × 10^3^	8.3415 × 10^4^	1.7055 × 10^4^	3.3281 × 10^3^	9.1385 × 10^3^	1.6470 × 10^4^	6.5157 × 10^9^
	SD	2.6066 × 10^0^	6.7661 × 10^1^	2.9047 × 10^5^	1.3881 × 10^4^	2.2348 × 10^3^	8.9083 × 10^3^	7.8562 × 10^3^	0.0000 × 10^0^
	Best	1.5061 × 10^3^	1.5564 × 10^3^	2.5407 × 10^3^	2.1123 × 10^3^	1.6378 × 10^3^	1.9701 × 10^3^	6.7731 × 10^3^	6.5157 × 10^9^
	Worst	1.5173 × 10^3^	1.8189 × 10^3^	1.3730 × 10^6^	4.0913 × 10^4^	1.1128 × 10^4^	3.5962 × 10^4^	4.0042 × 10^4^	6.5157 × 10^9^
	Rank	1	4	15	11	5	8	10	16
F16	Mean	1.9251 × 10^3^	2.5907 × 10^3^	2.8371 × 10^3^	2.5963 × 10^3^	2.4968 × 10^3^	2.3700 × 10^3^	2.6808 × 10^3^	2.7334 × 10^4^
	SD	1.8669 × 10^2^	3.5885 × 10^2^	3.0518 × 10^2^	3.4957 × 10^2^	1.7502 × 10^2^	3.0282 × 10^2^	2.6380 × 10^2^	7.4003 × 10^−12^
	Best	1.6297 × 10^3^	1.9778 × 10^3^	2.1008 × 10^3^	1.9930 × 10^3^	2.0124 × 10^3^	1.9413 × 10^3^	2.2329 × 10^3^	2.7334 × 10^4^
	Worst	2.3053 × 10^3^	3.5528 × 10^3^	3.3413 × 10^3^	3.3922 × 10^3^	2.8635 × 10^3^	3.0221 × 10^3^	3.4490 × 10^3^	2.7334 × 10^4^
	Rank	1	10	14	11	9	8	13	16
F17	Mean	1.7617 × 10^3^	2.0398 × 10^3^	2.1301 × 10^3^	2.2077 × 10^3^	1.9374 × 10^3^	2.0461 × 10^3^	2.1272 × 10^3^	2.8557 × 10^5^
	SD	2.7223 × 10^1^	8.9632 × 10^1^	2.1118 × 10^2^	2.6283 × 10^2^	7.0692 × 10^1^	1.3760 × 10^2^	1.4788 × 10^2^	1.1841 × 10^−10^
	Best	1.7332 × 10^3^	1.9053 × 10^3^	1.8137 × 10^3^	1.7919 × 10^3^	1.7855 × 10^3^	1.8320 × 10^3^	1.8772 × 10^3^	2.8557 × 10^5^
	Worst	1.8744 × 10^3^	2.2485 × 10^3^	2.6878 × 10^3^	2.7175 × 10^3^	2.1351 × 10^3^	2.3592 × 10^3^	2.4195 × 10^3^	2.8557 × 10^5^
	Rank	1	9	12	14	8	10	11	16
F18	Mean	1.8273 × 10^3^	1.9537 × 10^3^	9.9141 × 10^5^	4.9977 × 10^4^	8.6990 × 10^4^	6.4418 × 10^5^	4.3250 × 10^4^	4.7363 × 10^9^
	SD	3.7725 × 10^0^	7.1992 × 10^1^	1.1736 × 10^6^	9.6863 × 10^4^	3.6110 × 10^4^	6.8176 × 10^5^	4.7279 × 10^4^	0.0000 × 10^0^
	Best	1.8111 × 10^3^	1.8754 × 10^3^	8.3849 × 10^4^	7.8035 × 10^3^	4.3226 × 10^4^	1.0383 × 10^5^	5.5284 × 10^3^	4.7363 × 10^9^
	Worst	1.8329 × 10^3^	2.1290 × 10^3^	5.3656 × 10^6^	5.4656 × 10^5^	2.0837 × 10^5^	2.9183 × 10^6^	2.6664 × 10^5^	4.7363 × 10^9^
	Rank	1	4	15	6	9	14	5	16
F19	Mean	1.9116 × 10^3^	1.9459 × 10^3^	1.1715 × 10^5^	1.9424 × 10^4^	7.6671 × 10^3^	7.9661 × 10^3^	5.6184 × 10^4^	6.6479 × 10^9^
	SD	2.4247 × 10^0^	3.5982 × 10^1^	2.0513 × 10^5^	1.6613 × 10^4^	3.8390 × 10^3^	1.1401 × 10^4^	1.1043 × 10^5^	1.9400 × 10^−6^
	Best	1.9076 × 10^3^	1.9243 × 10^3^	3.7066 × 10^3^	2.2305 × 10^3^	2.0982 × 10^3^	2.0989 × 10^3^	5.0213 × 10^3^	6.6479 × 10^9^
	Worst	1.9159 × 10^3^	2.0788 × 10^3^	9.0356 × 10^5^	5.2798 × 10^4^	1.4818 × 10^4^	4.5040 × 10^4^	6.1435 × 10^5^	6.6479 × 10^9^
	Rank	1	4	14	11	6	8	13	16
F20	Mean	2.0793 × 10^3^	2.3480 × 10^3^	2.4383 × 10^3^	2.4766 × 10^3^	2.3565 × 10^3^	2.3864 × 10^3^	2.4434 × 10^3^	5.4969 × 10^3^
	SD	5.6942 × 10^1^	1.8023 × 10^2^	1.3478 × 10^2^	2.2811 × 10^2^	6.5716 × 10^1^	1.1771 × 10^2^	9.1942 × 10^1^	9.2504 × 10^−13^
	Best	2.0031 × 10^3^	2.1617 × 10^3^	2.2432 × 10^3^	2.1305 × 10^3^	2.2707 × 10^3^	2.1950 × 10^3^	2.3241 × 10^3^	5.4969 × 10^3^
	Worst	2.1773 × 10^3^	2.7325 × 10^3^	2.7064 × 10^3^	2.9824 × 10^3^	2.4916 × 10^3^	2.5963 × 10^3^	2.7017 × 10^3^	5.4969 × 10^3^
	Rank	1	8	12	14	9	10	13	16

**Table A3:** Statistical results of the 16 algorithms based on the CEC 2017 benchmark functions (F21 to F29 for the first eight algorithms).

Functions	Index	MSESFOA	SFOA	SHO	FLA	TTAO	COA	BKA	IVY
F21	Mean	2.3095 × 10^3^	2.4157 × 10^3^	2.4611 × 10^3^	2.4189 × 10^3^	2.3893 × 10^3^	2.4205 × 10^3^	2.4938 × 10^3^	3.2361 × 10^3^
	SD	7.8386 × 10^1^	3.6642 × 10^1^	2.3919 × 10^1^	3.1493 × 10^1^	1.4560 × 10^1^	3.1170 × 10^1^	3.8495 × 10^1^	1.3876 × 10^−12^
	Best	2.1700 × 10^3^	2.3620 × 10^3^	2.4126 × 10^3^	2.3717 × 10^3^	2.3477 × 10^3^	2.3698 × 10^3^	2.4455 × 10^3^	3.2361 × 10^3^
	Worst	2.3897 × 10^3^	2.5045 × 10^3^	2.5058 × 10^3^	2.4610 × 10^3^	2.4180 × 10^3^	2.4796 × 10^3^	2.6080 × 10^3^	3.2361 × 10^3^
	Rank	1	9	12	10	8	11	14	16
F22	Mean	2.3049 × 10^3^	2.3087 × 10^3^	4.9220 × 10^3^	5.1111 × 10^3^	2.3083 × 10^3^	2.6837 × 10^3^	4.8228 × 10^3^	1.2585 × 10^4^
	SD	1.8010 × 10^1^	2.7509 × 10^1^	1.5507 × 10^3^	1.9386 × 10^3^	2.7535 × 10^1^	1.2787 × 10^3^	2.2495 × 10^3^	2.5414 × 10^3^
	Best	2.3000 × 10^3^	2.3001 × 10^3^	2.4891 × 10^3^	2.3000 × 10^3^	2.3000 × 10^3^	2.3001 × 10^3^	2.3370 × 10^3^	3.2361 × 10^3^
	Worst	2.3711 × 10^3^	2.4090 × 10^3^	7.6926 × 10^3^	7.3742 × 10^3^	2.4093 × 10^3^	7.3291 × 10^3^	8.3278 × 10^3^	1.3253 × 10^4^
	Rank	1	3	13	15	2	6	12	16
F23	Mean	2.6609 × 10^3^	2.7696 × 10^3^	2.8997 × 10^3^	2.8028 × 10^3^	2.7488 × 10^3^	2.7892 × 10^3^	3.0242 × 10^3^	8.0606 × 10^3^
	SD	1.1230 × 10^2^	4.1583 × 10^1^	5.1792 × 10^1^	3.1273 × 10^1^	1.7995 × 10^1^	3.7069 × 10^1^	8.4822 × 10^1^	1.2636 × 10^−2^
	Best	2.4000 × 10^3^	2.7144 × 10^3^	2.8321 × 10^3^	2.7417 × 10^3^	2.7158 × 10^3^	2.7341 × 10^3^	2.8850 × 10^3^	8.0606 × 10^3^
	Worst	2.7379 × 10^3^	2.8628 × 10^3^	3.0321 × 10^3^	2.8452 × 10^3^	2.7838 × 10^3^	2.8400 × 10^3^	3.2033 × 10^3^	8.0606 × 10^3^
	Rank	1	9	13	11	8	10	15	16
F24	Mean	2.8651 × 10^3^	2.9373 × 10^3^	3.1875 × 10^3^	2.9657 × 10^3^	2.9024 × 10^3^	2.9298 × 10^3^	3.1701 × 10^3^	5.1970 × 10^3^
	SD	5.2617 × 10^1^	2.6994 × 10^1^	6.1590 × 10^1^	4.8456 × 10^1^	1.8397 × 10^1^	4.4610 × 10^1^	9.5444 × 10^1^	2.7751 × 10^−12^
	Best	2.6786 × 10^3^	2.8881 × 10^3^	3.1017 × 10^3^	2.9013 × 10^3^	2.8641 × 10^3^	2.8650 × 10^3^	3.0159 × 10^3^	5.1970 × 10^3^
	Worst	2.9341 × 10^3^	2.9950 × 10^3^	3.3060 × 10^3^	3.0810 × 10^3^	2.9266 × 10^3^	3.0445 × 10^3^	3.3776 × 10^3^	5.1970 × 10^3^
	Rank	2	10	14	11	8	9	13	16
F25	Mean	2.8858 × 10^3^	2.8873 × 10^3^	3.2145 × 10^3^	2.8935 × 10^3^	2.9097 × 10^3^	2.8933 × 10^3^	2.9338 × 10^3^	9.2455 × 10^3^
	SD	1.8151 × 10^0^	1.2997 × 10^0^	1.0118 × 10^2^	1.3554 × 10^1^	1.3218 × 10^1^	1.5067 × 10^1^	1.2250 × 10^2^	1.0416 × 10^−2^
	Best	2.8800 × 10^3^	2.8835 × 10^3^	3.1016 × 10^3^	2.8835 × 10^3^	2.8907 × 10^3^	2.8839 × 10^3^	2.8847 × 10^3^	9.2455 × 10^3^
	Worst	2.8869 × 10^3^	2.8898 × 10^3^	3.4269 × 10^3^	2.9246 × 10^3^	2.9401 × 10^3^	2.9349 × 10^3^	3.5620 × 10^3^	9.2455 × 10^3^
	Rank	1	5	15	9	12	8	13	16
F26	Mean	3.1915 × 10^3^	3.9823 × 10^3^	6.3331 × 10^3^	5.1391 × 10^3^	4.0175 × 10^3^	4.6012 × 10^3^	6.3194 × 10^3^	1.6233 × 10^4^
	SD	6.0967 × 10^2^	1.0066 × 10^3^	4.8941 × 10^2^	1.1263 × 10^3^	1.0009 × 10^3^	1.4107 × 10^3^	1.7420 × 10^3^	9.2504 × 10^−12^
	Best	2.8000 × 10^3^	2.8006 × 10^3^	5.5785 × 10^3^	2.9000 × 10^3^	2.8000 × 10^3^	2.8005 × 10^3^	2.9069 × 10^3^	1.6233 × 10^4^
	Worst	4.5855 × 10^3^	5.4278 × 10^3^	7.4813 × 10^3^	6.4873 × 10^3^	5.2586 × 10^3^	7.1022 × 10^3^	8.2087 × 10^3^	1.6233 × 10^4^
	Rank	1	3	14	11	4	10	13	16
F27	Mean	3.1999 × 10^3^	3.2051 × 10^3^	3.3660 × 10^3^	3.2532 × 10^3^	3.2384 × 10^3^	3.2716 × 10^3^	3.3069 × 10^3^	1.0647 × 10^4^
	SD	1.0128 × 10^1^	9.1934 × 10^0^	4.9261 × 10^1^	2.9091 × 10^1^	1.4803 × 10^1^	3.9035 × 10^1^	5.1338 × 10^1^	3.7002 × 10^−12^
	Best	3.1801 × 10^3^	3.1842 × 10^3^	3.3109 × 10^3^	3.2152 × 10^3^	3.2172 × 10^3^	3.2183 × 10^3^	3.2302 × 10^3^	1.0647 × 10^4^
	Worst	3.2193 × 10^3^	3.2212 × 10^3^	3.4806 × 10^3^	3.3186 × 10^3^	3.2730 × 10^3^	3.3357 × 10^3^	3.4265 × 10^3^	1.0647 × 10^4^
	Rank	1	3	15	11	9	12	13	16
F28	Mean	3.1077 × 10^3^	3.2036 × 10^3^	3.8537 × 10^3^	3.1509 × 10^3^	3.2018 × 10^3^	3.2117 × 10^3^	3.5473 × 10^3^	1.0248 × 10^4^
	SD	2.8893 × 10^1^	4.3964 × 10^1^	3.2289 × 10^2^	5.5717 × 10^1^	1.0368 × 10^1^	3.7466 × 10^1^	6.0564 × 10^2^	1.8501 × 10^−12^
	Best	3.1000 × 10^3^	3.1148 × 10^3^	3.4608 × 10^3^	3.1000 × 10^3^	3.1840 × 10^3^	3.1183 × 10^3^	3.2078 × 10^3^	1.0248 × 10^4^
	Worst	3.2140 × 10^3^	3.2787 × 10^3^	4.6129 × 10^3^	3.2401 × 10^3^	3.2259 × 10^3^	3.2784 × 10^3^	4.9024 × 10^3^	1.0248 × 10^4^
	Rank	1	8	15	4	7	9	13	16
F29	Mean	3.3862 × 10^3^	3.7194 × 10^3^	4.0163 × 10^3^	4.0494 × 10^3^	3.7605 × 10^3^	3.8264 × 10^3^	4.2290 × 10^3^	2.3891 × 10^5^
	SD	6.6669 × 10^1^	1.5530 × 10^2^	2.3952 × 10^2^	2.6122 × 10^2^	7.5951 × 10^1^	2.0862 × 10^2^	4.0359 × 10^2^	5.9203 × 10^−11^
	Best	3.2250 × 10^3^	3.5101 × 10^3^	3.5536 × 10^3^	3.5659 × 10^3^	3.5724 × 10^3^	3.4477 × 10^3^	3.8509 × 10^3^	2.3891 × 10^5^
	Worst	3.5331 × 10^3^	4.0603 × 10^3^	4.6439 × 10^3^	4.5414 × 10^3^	3.8777 × 10^3^	4.2932 × 10^3^	5.2415 × 10^3^	2.3891 × 10^5^
	Rank	1	8	12	13	9	10	14	16

**Table A4:** Statistical results of the 16 algorithms based on the CEC 2017 benchmark functions (F30 for the first eight algorithms).

Functions	Index	MSESFOA	SFOA	SHO	FLA	TTAO	COA	BKA	IVY
F30	Mean	4.9536 × 10^3^	5.6099 × 10^3^	2.4271 × 10^6^	1.1102 × 10^4^	7.0566 × 10^3^	2.2291 × 10^4^	9.8044 × 10^5^	1.0275 × 10^10^
	SD	1.7379 × 10^2^	9.0575 × 10^2^	3.1986 × 10^6^	3.7252 × 10^3^	1.2922 × 10^3^	1.1100 × 10^4^	2.3939 × 10^6^	1.9400 × 10^−6^
	Best	4.0584 × 10^3^	4.9514 × 10^3^	9.7571 × 10^4^	5.0394 × 10^3^	5.4761 × 10^3^	8.6388 × 10^3^	2.9878 × 10^4^	1.0275 × 10^10^
	Worst	5.0905 × 10^3^	9.2566 × 10^3^	1.5561 × 10^7^	2.0362 × 10^4^	1.0279 × 10^4^	4.5512 × 10^4^	9.7422 × 10^6^	1.0275 × 10^10^
	Rank	1	3	14	8	5	11	13	16
Mean rank		1.2759	7.0690	13.5862	9.2759	7.3103	10.5172	12.1724	15.9655
Final rank		1	7	14	10	8	11	13	16

**Table A5:** Statistical results of the 16 algorithms based on the CEC 2017 benchmark functions (F1 to F8 for the second eight algorithms).

Functions	Index	NRBO	PKO	EAO	ALA	RIME	IRIME	SAO	ISAO
F1	Mean	8.0429 × 10^9^	1.8577 × 10^3^	1.0008 × 10^2^	1.5376 × 10^3^	2.3710 × 10^4^	1.1033 × 10^4^	5.2897 × 10^3^	4.8892 × 10^3^
	SD	1.8533 × 10^9^	1.5384 × 10^3^	1.6421 × 10^−1^	1.5217 × 10^3^	1.0404 × 10^4^	7.6348 × 10^3^	5.9613 × 10^3^	5.4491 × 10^3^
	Best	4.4798 × 10^9^	1.0173 × 10^2^	1.0000 × 10^2^	1.0392 × 10^2^	9.7359 × 10^3^	2.3687 × 10^2^	1.0626 × 10^2^	1.0092 × 10^2^
	Worst	1.2294 × 10^10^	4.9550 × 10^3^	1.0068 × 10^2^	6.0552 × 10^3^	5.0014 × 10^4^	2.2480 × 10^4^	2.0520 × 10^4^	1.9772 × 10^4^
	Rank	14	4	2	3	12	10	8	7
F3	Mean	2.8940 × 10^4^	1.3821 × 10^4^	3.0001 × 10^2^	3.0000 × 10^2^	3.1277 × 10^2^	3.0011 × 10^2^	3.1491 × 10^4^	2.8824 × 10^4^
	SD	6.6362 × 10^3^	6.1747 × 10^3^	3.0868 × 10^−2^	1.8125 × 10^−5^	8.6146 × 10^0^	7.5614 × 10^−2^	7.0525 × 10^3^	7.6540 × 10^3^
	Best	1.2682 × 10^4^	4.4350 × 10^3^	3.0000 × 10^2^	3.0000 × 10^2^	3.0562 × 10^2^	3.0003 × 10^2^	2.0812 × 10^4^	1.5808 × 10^4^
	Worst	3.8834 × 10^4^	2.9076 × 10^4^	3.0017 × 10^2^	3.0000 × 10^2^	3.5159 × 10^2^	3.0035 × 10^2^	4.5658 × 10^4^	5.3528 × 10^4^
	Rank	13	10	4	3	7	5	14	12
F4	Mean	9.3854 × 10^2^	4.9465 × 10^2^	4.5468 × 10^2^	4.6379 × 10^2^	4.9309 × 10^2^	4.9666 × 10^2^	4.5198 × 10^2^	4.5370 × 10^2^
	SD	1.9352 × 10^2^	1.5193 × 10^1^	1.8756 × 10^1^	1.6639 × 10^1^	1.6197 × 10^1^	2.3083 × 10^1^	2.3210 × 10^1^	1.9998 × 10^1^
	Best	7.0157 × 10^2^	4.6931 × 10^2^	4.0000 × 10^2^	4.0399 × 10^2^	4.5859 × 10^2^	4.1949 × 10^2^	4.0001 × 10^2^	4.0021 × 10^2^
	Worst	1.5708 × 10^3^	5.1846 × 10^2^	4.6790 × 10^2^	5.0819 × 10^2^	5.2047 × 10^2^	5.2260 × 10^2^	4.7374 × 10^2^	4.7153 × 10^2^
	Rank	13	11	5	6	10	12	3	4
F5	Mean	7.7988 × 10^2^	5.5226 × 10^2^	5.4145 × 10^2^	5.6214 × 10^2^	5.6413 × 10^2^	6.5441 × 10^2^	5.4768 × 10^2^	5.4685 × 10^2^
	SD	3.6514 × 10^1^	1.6136 × 10^1^	2.3177 × 10^1^	1.8779 × 10^1^	1.4168 × 10^1^	3.6597 × 10^1^	1.6515 × 10^1^	1.5504 × 10^1^
	Best	7.0084 × 10^2^	5.2189 × 10^2^	5.1890 × 10^2^	5.2885 × 10^2^	5.3781 × 10^2^	5.8457 × 10^2^	5.1592 × 10^2^	5.1890 × 10^2^
	Worst	8.5675 × 10^2^	5.7562 × 10^2^	6.2388 × 10^2^	5.9595 × 10^2^	5.9285 × 10^2^	7.2260 × 10^2^	5.9084 × 10^2^	5.8980 × 10^2^
	Rank	15	5	2	6	7	11	4	3
F6	Mean	6.6712 × 10^2^	6.0001 × 10^2^	6.0000 × 10^2^	6.0008 × 10^2^	6.0034 × 10^2^	6.0576 × 10^2^	6.0000 × 10^2^	6.0000 × 10^2^
	SD	6.8758 × 10^0^	2.1280 × 10^−2^	2.1722 × 10^−3^	1.1119 × 10^−1^	1.3175 × 10^−1^	4.5941 × 10^0^	1.4102 × 10^−4^	4.3569 × 10^−3^
	Best	6.5614 × 10^2^	6.0000 × 10^2^	6.0000 × 10^2^	6.0001 × 10^2^	6.0014 × 10^2^	6.0052 × 10^2^	6.0000 × 10^2^	6.0000 × 10^2^
	Worst	6.7675 × 10^2^	6.0007 × 10^2^	6.0001 × 10^2^	6.0055 × 10^2^	6.0064 × 10^2^	6.1795 × 10^2^	6.0000 × 10^2^	6.0002 × 10^2^
	Rank	15	5	3	6	7	9	2	4
F7	Mean	1.1242 × 10^3^	7.7914 × 10^2^	7.5441 × 10^2^	8.2506 × 10^2^	7.9542 × 10^2^	9.0934 × 10^2^	7.6503 × 10^2^	7.6089 × 10^2^
	SD	6.6681 × 10^1^	1.3936 × 10^1^	7.3837 × 10^0^	2.1673 × 10^1^	2.2273 × 10^1^	3.7586 × 10^1^	1.1794 × 10^1^	8.8025 × 10^0^
	Best	9.9701 × 10^2^	7.5682 × 10^2^	7.3753 × 10^2^	7.6794 × 10^2^	7.6201 × 10^2^	8.4757 × 10^2^	7.5008 × 10^2^	7.4786 × 10^2^
	Worst	1.2486 × 10^3^	8.0111 × 10^2^	7.6783 × 10^2^	8.6220 × 10^2^	8.3812 × 10^2^	9.7763 × 10^2^	7.9241 × 10^2^	7.8856 × 10^2^
	Rank	14	5	1	8	6	10	3	2
F8	Mean	1.0237 × 10^3^	8.5542 × 10^2^	8.3185 × 10^2^	8.7510 × 10^2^	8.6477 × 10^2^	9.8899 × 10^2^	8.4422 × 10^2^	8.4398 × 10^2^
	SD	3.5912 × 10^1^	1.6703 × 10^1^	1.0394 × 10^1^	2.4800 × 10^1^	2.0017 × 10^1^	4.3391 × 10^1^	9.8716 × 10^0^	1.4085 × 10^1^
	Best	9.4281 × 10^2^	8.2189 × 10^2^	8.1293 × 10^2^	8.3482 × 10^2^	8.2688 × 10^2^	8.9850 × 10^2^	8.2489 × 10^2^	8.1890 × 10^2^
	Worst	1.0769 × 10^3^	8.8756 × 10^2^	8.4776 × 10^2^	9.4408 × 10^2^	9.0619 × 10^2^	1.0736 × 10^3^	8.6865 × 10^2^	8.8159 × 10^2^
	Rank	15	4	1	7	6	14	3	2

**Table A6:** Statistical results of the 16 algorithms based on the CEC 2017 benchmark functions (F9 to F17 for the second eight algorithms).

Functions	Index	NRBO	PKO	EAO	ALA	RIME	IRIME	SAO	ISAO
F9	Mean	4.9820 × 10^3^	9.1129 × 10^2^	9.0083 × 10^2^	1.0064 × 10^3^	9.4775 × 10^2^	5.2065 × 10^3^	9.0817 × 10^2^	9.0006 × 10^2^
	SD	8.9718 × 10^2^	2.4434 × 10^1^	7.9286 × 10^−1^	8.9019 × 10^1^	9.2505 × 10^1^	2.3325 × 10^3^	1.7589 × 10^1^	1.3736 × 10^−1^
	Best	3.4506 × 10^3^	9.0000 × 10^2^	9.0000 × 10^2^	9.1612 × 10^2^	9.0726 × 10^2^	1.8349 × 10^3^	9.0000 × 10^2^	9.0000 × 10^2^
	Worst	6.9106 × 10^3^	9.7968 × 10^2^	9.0273 × 10^2^	1.2111 × 10^3^	1.2640 × 10^3^	1.0585 × 10^4^	9.5827 × 10^2^	9.0054 × 10^2^
	Rank	14	5	3	7	6	15	4	2
F10	Mean	6.9789 × 10^3^	4.5782 × 10^3^	7.6165 × 10^3^	4.4213 × 10^3^	3.7415 × 10^3^	4.5149 × 10^3^	3.7350 × 10^3^	3.5436 × 10^3^
	SD	5.8528 × 10^2^	4.9813 × 10^2^	8.6521 × 10^2^	4.8436 × 10^2^	5.7916 × 10^2^	6.6378 × 10^2^	5.7407 × 10^2^	5.3291 × 10^2^
	Best	5.3267 × 10^3^	3.6725 × 10^3^	3.2547 × 10^3^	3.0292 × 10^3^	2.1356 × 10^3^	3.0401 × 10^3^	2.7765 × 10^3^	2.3586 × 10^3^
	Worst	8.5347 × 10^3^	5.7672 × 10^3^	8.2173 × 10^3^	5.2315 × 10^3^	4.6667 × 10^3^	5.7023 × 10^3^	5.0237 × 10^3^	4.8635 × 10^3^
	Rank	13	8	15	5	4	7	3	2
F11	Mean	1.7022 × 10^3^	1.1412 × 10^3^	1.1260 × 10^3^	1.1479 × 10^3^	1.2310 × 10^3^	1.2834 × 10^3^	1.1367 × 10^3^	1.1436 × 10^3^
	SD	2.1083 × 10^2^	2.9348 × 10^1^	2.5201 × 10^1^	2.7269 × 10^1^	3.7115 × 10^1^	5.7581 × 10^1^	2.9441 × 10^1^	3.3637 × 10^1^
	Best	1.4666 × 10^3^	1.1071 × 10^3^	1.1050 × 10^3^	1.1120 × 10^3^	1.1636 × 10^3^	1.1469 × 10^3^	1.1091 × 10^3^	1.1111 × 10^3^
	Worst	2.6854 × 10^3^	1.2094 × 10^3^	1.1899 × 10^3^	1.2102 × 10^3^	1.3051 × 10^3^	1.3785 × 10^3^	1.2031 × 10^3^	1.2426 × 10^3^
	Rank	14	4	2	6	9	12	3	5
F12	Mean	4.7420 × 10^8^	1.7003 × 10^5^	2.7292 × 10^3^	3.9031 × 10^3^	2.2998 × 10^6^	2.9158 × 10^6^	3.6253 × 10^4^	3.8951 × 10^4^
	SD	2.1735 × 10^8^	1.2346 × 10^5^	6.8702 × 10^2^	9.9095 × 10^2^	1.7353 × 10^6^	1.8376 × 10^6^	1.6980 × 10^4^	2.2317 × 10^4^
	Best	9.5059 × 10^7^	1.6696 × 10^4^	1.7281 × 10^3^	2.6766 × 10^3^	1.8848 × 10^5^	2.7127 × 10^5^	8.7070 × 10^3^	3.3220 × 10^3^
	Worst	8.6963 × 10^8^	5.2826 × 10^5^	4.9549 × 10^3^	6.8979 × 10^3^	6.4494 × 10^6^	6.2508 × 10^6^	7.4660 × 10^4^	8.6939 × 10^4^
	Rank	15	9	2	3	11	12	7	8
F13	Mean	2.9641 × 10^6^	3.0038 × 10^4^	1.7177 × 10^3^	1.4545 × 10^3^	2.2113 × 10^4^	1.3585 × 10^4^	1.6035 × 10^4^	2.2166 × 10^4^
	SD	7.9621 × 10^6^	2.2659 × 10^4^	2.7745 × 10^2^	4.1154 × 10^1^	1.7292 × 10^4^	1.5732 × 10^4^	1.6033 × 10^4^	2.1816 × 10^4^
	Best	6.4567 × 10^4^	1.4566 × 10^3^	1.3901 × 10^3^	1.3635 × 10^3^	2.6510 × 10^3^	1.6876 × 10^3^	1.5922 × 10^3^	1.3270 × 10^3^
	Worst	4.3952 × 10^7^	6.2733 × 10^4^	2.5506 × 10^3^	1.5612 × 10^3^	5.8662 × 10^4^	5.7588 × 10^4^	5.7585 × 10^4^	6.2180 × 10^4^
	Rank	14	10	3	2	8	6	7	9
F14	Mean	1.2512 × 10^9^	4.7833 × 10^3^	1.2212 × 10^4^	1.4324 × 10^3^	1.4539 × 10^3^	1.1073 × 10^4^	1.5226 × 10^4^	8.5999 × 10^3^
	SD	7.2748 × 10^−7^	5.3968 × 10^3^	7.6772 × 10^3^	4.5013 × 10^0^	1.0977 × 10^1^	9.9533 × 10^3^	9.0973 × 10^3^	4.7293 × 10^3^
	Best	1.2512 × 10^9^	1.6063 × 10^3^	2.3910 × 10^3^	1.4232 × 10^3^	1.4382 × 10^3^	1.8720 × 10^3^	1.4946 × 10^3^	1.4762 × 10^3^
	Worst	1.2512 × 10^9^	2.9634 × 10^4^	3.8613 × 10^4^	1.4402 × 10^3^	1.4843 × 10^3^	4.7779 × 10^4^	3.4620 × 10^4^	1.6383 × 10^4^
	Rank	15	8	11	2	3	10	12	9
F15	Mean	3.2170 × 10^4^	2.1126 × 10^4^	1.5648 × 10^3^	1.5687 × 10^3^	9.9240 × 10^3^	2.0100 × 10^4^	5.0211 × 10^3^	4.6520 × 10^3^
	SD	2.7293 × 10^4^	1.5647 × 10^4^	3.8272 × 10^1^	3.0504 × 10^1^	1.1354 × 10^4^	1.5567 × 10^4^	5.3608 × 10^3^	2.8414 × 10^3^
	Best	3.7733 × 10^3^	1.5221 × 10^3^	1.5241 × 10^3^	1.5348 × 10^3^	1.7169 × 10^3^	1.7150 × 10^3^	1.5579 × 10^3^	1.6771 × 10^3^
	Worst	1.0782 × 10^5^	4.2703 × 10^4^	1.6730 × 10^3^	1.6655 × 10^3^	3.7415 × 10^4^	4.2902 × 10^4^	2.6699 × 10^4^	1.2085 × 10^4^
	Rank	14	13	2	3	9	12	7	6
F16	Mean	3.3699 × 10^3^	2.2176 × 10^3^	1.9370 × 10^3^	2.1154 × 10^3^	2.2961 × 10^3^	2.6393 × 10^3^	2.3139 × 10^3^	2.2105 × 10^3^
	SD	3.3177 × 10^2^	1.8923 × 10^2^	3.9607 × 10^2^	2.2138 × 10^2^	2.1758 × 10^2^	2.7905 × 10^2^	2.4908 × 10^2^	2.2509 × 10^2^
	Best	2.5475 × 10^3^	1.8400 × 10^3^	1.6172 × 10^3^	1.7524 × 10^3^	1.8499 × 10^3^	2.2691 × 10^3^	1.7545 × 10^3^	1.8658 × 10^3^
	Worst	3.9430 × 10^3^	2.7700 × 10^3^	2.9722 × 10^3^	2.7228 × 10^3^	2.7320 × 10^3^	3.3115 × 10^3^	2.7485 × 10^3^	2.7539 × 10^3^
	Rank	15	5	2	3	6	12	7	4
F17	Mean	2.3766 × 10^3^	1.8292 × 10^3^	1.7862 × 10^3^	1.8677 × 10^3^	1.8916 × 10^3^	2.1784 × 10^3^	1.9203 × 10^3^	1.9325 × 10^3^
	SD	1.9529 × 10^2^	7.1593 × 10^1^	4.3817 × 10^1^	8.0386 × 10^1^	1.0797 × 10^2^	1.6465 × 10^2^	1.8530 × 10^2^	1.4434 × 10^2^
	Best	2.0353 × 10^3^	1.7439 × 10^3^	1.7411 × 10^3^	1.7680 × 10^3^	1.7623 × 10^3^	1.8774 × 10^3^	1.7284 × 10^3^	1.7410 × 10^3^
	Worst	2.7372 × 10^3^	2.0435 × 10^3^	1.9473 × 10^3^	2.0877 × 10^3^	2.1508 × 10^3^	2.4643 × 10^3^	2.3237 × 10^3^	2.3295 × 10^3^
	Rank	15	3	2	4	5	13	6	7

**Table A7:** Statistical results of the 16 algorithms based on the CEC 2017 benchmark functions (F18 to F26 for the second eight algorithms).

Functions	Index	NRBO	PKO	EAO	ALA	RIME	IRIME	SAO	ISAO
F18	Mean	2.7917 × 10^5^	2.2028 × 10^5^	1.8468 × 10^3^	1.8654 × 10^3^	2.4652 × 10^5^	2.2935 × 10^5^	7.6306 × 10^4^	8.6333 × 10^4^
	SD	3.2203 × 10^5^	1.3095 × 10^5^	2.1954 × 10^1^	1.3974 × 10^1^	2.5576 × 10^5^	1.3354 × 10^5^	5.7813 × 10^4^	4.5412 × 10^4^
	Best	1.9582 × 10^4^	6.4795 × 10^4^	1.8245 × 10^3^	1.8404 × 10^3^	6.0832 × 10^4^	4.6613 × 10^4^	1.6989 × 10^4^	2.6028 × 10^4^
	Worst	1.3845 × 10^6^	5.9045 × 10^5^	1.9290 × 10^3^	1.8969 × 10^3^	1.3697 × 10^6^	6.3345 × 10^5^	2.7185 × 10^5^	2.3901 × 10^5^
	Rank	13	10	2	3	12	11	7	8
F19	Mean	3.6611 × 10^5^	2.2836 × 10^4^	1.9161 × 10^3^	1.9279 × 10^3^	1.2515 × 10^4^	1.3818 × 10^4^	5.1288 × 10^3^	7.7625 × 10^3^
	SD	5.0003 × 10^5^	2.2603 × 10^4^	3.2936 × 10^0^	7.3857 × 10^0^	1.0877 × 10^4^	1.4173 × 10^4^	2.6056 × 10^3^	8.9114 × 10^3^
	Best	1.5657 × 10^4^	1.9905 × 10^3^	1.9109 × 10^3^	1.9195 × 10^3^	2.2363 × 10^3^	1.9917 × 10^3^	1.9145 × 10^3^	2.2392 × 10^3^
	Worst	1.7980 × 10^6^	5.6283 × 10^4^	1.9238 × 10^3^	1.9428 × 10^3^	3.4078 × 10^4^	4.8196 × 10^4^	9.0833 × 10^3^	3.9395 × 10^4^
	Rank	15	12	2	3	9	10	5	7
F20	Mean	2.6450 × 10^3^	2.1935 × 10^3^	2.1258 × 10^3^	2.1474 × 10^3^	2.1953 × 10^3^	2.4233 × 10^3^	2.2602 × 10^3^	2.2853 × 10^3^
	SD	1.4175 × 10^2^	1.0128 × 10^2^	6.1077 × 10^1^	9.4849 × 10^1^	7.2333 × 10^1^	1.4811 × 10^2^	1.6011 × 10^2^	1.3201 × 10^2^
	Best	2.4211 × 10^3^	2.0455 × 10^3^	2.0519 × 10^3^	2.0353 × 10^3^	2.0621 × 10^3^	2.0740 × 10^3^	2.0285 × 10^3^	2.1497 × 10^3^
	Worst	3.0316 × 10^3^	2.4237 × 10^3^	2.2559 × 10^3^	2.3520 × 10^3^	2.3169 × 10^3^	2.6487 × 10^3^	2.5181 × 10^3^	2.5929 × 10^3^
	Rank	15	4	2	3	5	11	6	7
F21	Mean	2.5647 × 10^3^	2.3413 × 10^3^	2.3270 × 10^3^	2.3688 × 10^3^	2.3673 × 10^3^	2.4661 × 10^3^	2.3456 × 10^3^	2.3455 × 10^3^
	SD	4.4152 × 10^1^	1.1318 × 10^1^	1.4510 × 10^1^	1.7527 × 10^1^	1.6250 × 10^1^	5.4099 × 10^1^	1.1688 × 10^1^	1.1962 × 10^1^
	Best	2.4687 × 10^3^	2.3233 × 10^3^	2.3069 × 10^3^	2.3444 × 10^3^	2.3388 × 10^3^	2.3838 × 10^3^	2.3244 × 10^3^	2.3289 × 10^3^
	Worst	2.6693 × 10^3^	2.3762 × 10^3^	2.3796 × 10^3^	2.4094 × 10^3^	2.3955 × 10^3^	2.5777 × 10^3^	2.3688 × 10^3^	2.3813 × 10^3^
	Rank	15	3	2	7	6	13	5	4
F22	Mean	4.1721 × 10^3^	4.3113 × 10^3^	2.8245 × 10^3^	3.6399 × 10^3^	3.2218 × 10^3^	4.9324 × 10^3^	2.4585 × 10^3^	2.6633 × 10^3^
	SD	1.8975 × 10^3^	2.0855 × 10^3^	1.7285 × 10^3^	1.9491 × 10^3^	1.3741 × 10^3^	1.9427 × 10^3^	4.5669 × 10^2^	9.3068 × 10^2^
	Best	2.5444 × 10^3^	2.3000 × 10^3^	2.3000 × 10^3^	2.3000 × 10^3^	2.3005 × 10^3^	2.3000 × 10^3^	2.3000 × 10^3^	2.3000 × 10^3^
	Worst	8.8993 × 10^3^	7.2553 × 10^3^	9.1081 × 10^3^	7.4889 × 10^3^	6.1686 × 10^3^	7.6198 × 10^3^	4.0251 × 10^3^	5.0582 × 10^3^
	Rank	10	11	7	9	8	14	4	5
F23	Mean	2.9654 × 10^3^	2.7017 × 10^3^	2.6856 × 10^3^	2.7331 × 10^3^	2.7194 × 10^3^	2.8867 × 10^3^	2.6986 × 10^3^	2.6886 × 10^3^
	SD	6.8532 × 10^1^	1.2257 × 10^1^	2.4700 × 10^1^	1.8887 × 10^1^	1.0536 × 10^1^	7.0673 × 10^1^	1.4912 × 10^1^	1.0714 × 10^1^
	Best	2.8707 × 10^3^	2.6772 × 10^3^	2.6619 × 10^3^	2.7025 × 10^3^	2.6997 × 10^3^	2.8066 × 10^3^	2.6784 × 10^3^	2.6651 × 10^3^
	Worst	3.1151 × 10^3^	2.7292 × 10^3^	2.7714 × 10^3^	2.7756 × 10^3^	2.7431 × 10^3^	3.0070 × 10^3^	2.7258 × 10^3^	2.7138 × 10^3^
	Rank	14	5	2	7	6	12	4	3
F24	Mean	3.1398 × 10^3^	2.8804 × 10^3^	2.8569 × 10^3^	2.8988 × 10^3^	2.8978 × 10^3^	3.2390 × 10^3^	2.8743 × 10^3^	2.8702 × 10^3^
	SD	4.9965 × 10^1^	1.7900 × 10^1^	5.5424 × 10^1^	1.6852 × 10^1^	2.0128 × 10^1^	1.1978 × 10^2^	1.5583 × 10^1^	2.4139 × 10^1^
	Best	3.0237 × 10^3^	2.8551 × 10^3^	2.8308 × 10^3^	2.8661 × 10^3^	2.8674 × 10^3^	3.0486 × 10^3^	2.8521 × 10^3^	2.8510 × 10^3^
	Worst	3.2368 × 10^3^	2.9202 × 10^3^	3.1424 × 10^3^	2.9314 × 10^3^	2.9445 × 10^3^	3.4502 × 10^3^	2.9246 × 10^3^	2.9511 × 10^3^
	Rank	12	5	1	7	6	15	4	3
F25	Mean	3.1854 × 10^3^	2.8870 × 10^3^	2.8869 × 10^3^	2.8868 × 10^3^	2.8940 × 10^3^	2.8976 × 10^3^	2.8874 × 10^3^	2.8873 × 10^3^
	SD	9.1030 × 10^1^	1.2523 × 10^0^	7.7418 × 10^−2^	1.1270 × 10^0^	1.6626 × 10^1^	2.3112 × 10^1^	3.7330 × 10^−1^	3.3742 × 10^−1^
	Best	3.0761 × 10^3^	2.8835 × 10^3^	2.8868 × 10^3^	2.8835 × 10^3^	2.8841 × 10^3^	2.8843 × 10^3^	2.8868 × 10^3^	2.8868 × 10^3^
	Worst	3.4111 × 10^3^	2.8886 × 10^3^	2.8870 × 10^3^	2.8874 × 10^3^	2.9392 × 10^3^	2.9681 × 10^3^	2.8879 × 10^3^	2.8878 × 10^3^
	Rank	14	4	3	2	10	11	7	6
F26	Mean	6.7873 × 10^3^	4.2100 × 10^3^	3.7966 × 10^3^	4.5381 × 10^3^	4.1516 × 10^3^	5.5652 × 10^3^	4.0263 × 10^3^	4.0660 × 10^3^
	SD	9.7937 × 10^2^	1.6153 × 10^2^	2.0998 × 10^2^	2.1684 × 10^2^	5.4016 × 10^2^	1.0908 × 10^3^	1.3804 × 10^2^	1.4303 × 10^2^
	Best	4.6154 × 10^3^	3.9996 × 10^3^	2.9000 × 10^3^	4.1209 × 10^3^	2.9005 × 10^3^	2.8007 × 10^3^	3.7726 × 10^3^	3.7823 × 10^3^
	Worst	8.5011 × 10^3^	4.5776 × 10^3^	4.2225 × 10^3^	4.8639 × 10^3^	4.8582 × 10^3^	7.1496 × 10^3^	4.2624 × 10^3^	4.3170 × 10^3^
	Rank	15	8	2	9	7	12	5	6

**Table A8:** Statistical results of the 16 algorithms based on the CEC 2017 benchmark functions (F27 to F30 for the second eight algorithms).

Functions	Index	NRBO	PKO	EAO	ALA	RIME	IRIME	SAO	ISAO
F27	Mean	3.3283 × 10^3^	3.2097 × 10^3^	3.2018 × 10^3^	3.2095 × 10^3^	3.2126 × 10^3^	3.2522 × 10^3^	3.2172 × 10^3^	3.2178 × 10^3^
	SD	6.4715 × 10^1^	2.4847 × 10^1^	8.1620 × 10^0^	1.1797 × 10^1^	8.2174 × 10^0^	2.1891 × 10^1^	1.1480 × 10^1^	2.5795 × 10^1^
	Best	3.2436 × 10^3^	3.1953 × 10^3^	3.1874 × 10^3^	3.1873 × 10^3^	3.1938 × 10^3^	3.2221 × 10^3^	3.2033 × 10^3^	3.1999 × 10^3^
	Worst	3.4457 × 10^3^	3.2987 × 10^3^	3.2134 × 10^3^	3.2325 × 10^3^	3.2240 × 10^3^	3.3051 × 10^3^	3.2454 × 10^3^	3.3076 × 10^3^
	Rank	14	5	2	4	6	10	7	8
F28	Mean	3.6892 × 10^3^	3.2181 × 10^3^	3.1272 × 10^3^	3.1424 × 10^3^	3.2330 × 10^3^	3.2297 × 10^3^	3.1541 × 10^3^	3.1525 × 10^3^
	SD	1.0712 × 10^2^	2.2784 × 10^1^	5.0783 × 10^1^	5.4514 × 10^1^	2.2382 × 10^1^	4.5578 × 10^1^	6.5758 × 10^1^	6.7734 × 10^1^
	Best	3.4271 × 10^3^	3.1889 × 10^3^	3.1000 × 10^3^	3.1000 × 10^3^	3.1983 × 10^3^	3.1002 × 10^3^	3.1000 × 10^3^	3.1000 × 10^3^
	Worst	3.9761 × 10^3^	3.2572 × 10^3^	3.2540 × 10^3^	3.2486 × 10^3^	3.2647 × 10^3^	3.3483 × 10^3^	3.2666 × 10^3^	3.2666 × 10^3^
	Rank	14	10	2	3	12	11	6	5
F29	Mean	4.6473 × 10^3^	3.4664 × 10^3^	3.4721 × 10^3^	3.5622 × 10^3^	3.6168 × 10^3^	3.9653 × 10^3^	3.5492 × 10^3^	3.4957 × 10^3^
	SD	3.0874 × 10^2^	9.3393 × 10^1^	3.9950 × 10^1^	1.3794 × 10^2^	1.4662 × 10^2^	2.1241 × 10^2^	1.7707 × 10^2^	1.3933 × 10^2^
	Best	3.9957 × 10^3^	3.3252 × 10^3^	3.3982 × 10^3^	3.2923 × 10^3^	3.4165 × 10^3^	3.6137 × 10^3^	3.3041 × 10^3^	3.3452 × 10^3^
	Worst	5.1470 × 10^3^	3.8213 × 10^3^	3.5389 × 10^3^	3.8446 × 10^3^	4.0443 × 10^3^	4.3609 × 10^3^	3.8746 × 10^3^	3.8189 × 10^3^
	Rank	15	2	3	6	7	11	5	4
F30	Mean	1.4360 × 10^7^	1.3711 × 10^4^	5.3873 × 10^3^	5.8753 × 10^3^	3.3878 × 10^4^	1.6543 × 10^4^	8.7498 × 10^3^	7.9475 × 10^3^
	SD	1.8074 × 10^7^	2.7012 × 10^3^	3.0221 × 10^2^	7.6433 × 10^2^	2.0363 × 10^4^	5.8815 × 10^3^	3.4494 × 10^3^	2.4962 × 10^3^
	Best	1.2999 × 10^6^	5.5489 × 10^3^	4.9842 × 10^3^	5.1111 × 10^3^	1.1497 × 10^4^	6.9578 × 10^3^	5.2105 × 10^3^	5.1244 × 10^3^
	Worst	9.2141 × 10^7^	1.7384 × 10^4^	6.4731 × 10^3^	8.3525 × 10^3^	9.9888 × 10^4^	2.8244 × 10^4^	1.5661 × 10^4^	1.4207 × 10^4^
	Rank	15	9	2	4	12	10	7	6
Mean rank		14.1034	6.7931	3.1724	4.8621	7.6552	11.0690	5.6897	5.4483
Final rank		15	6	2	3	9	12	5	4

## Appendix B. Statistical Results of the 16 Algorithms Based on the CEC 2022 Benchmark Functions

**Table A9:** Statistical results of the 16 algorithms based on the CEC 2022 benchmark functions (F1 to F9 for the first eight algorithms).

Functions	Index	MSESFOA	SFOA	SHO	FLA	TTAO	COA	BKA	IVY
F1	Mean	3.0000 × 10^2^	3.3841 × 10^2^	1.3083 × 10^4^	3.0003 × 10^2^	3.0139 × 10^2^	1.4685 × 10^4^	2.4841 × 10^3^	9.5587 × 10^12^
	SD	4.6488 × 10^−4^	3.0602 × 10^1^	3.4283 × 10^3^	1.1168 × 10^−1^	8.2622 × 10^−1^	6.2941 × 10^3^	4.4137 × 10^3^	1.9865 × 10^−3^
	Best	3.0000 × 10^2^	3.0720 × 10^2^	6.1435 × 10^3^	3.0000 × 10^2^	3.0018 × 10^2^	4.6475 × 10^3^	3.0152 × 10^2^	9.5587 × 10^12^
	Worst	3.0000 × 10^2^	4.6594 × 10^2^	2.2090 × 10^4^	3.0044 × 10^2^	3.0358 × 10^2^	2.5884 × 10^4^	1.3049 × 10^4^	9.5587 × 10^12^
	Rank	2	8	14	3	5	15	9	16
F2	Mean	4.3479 × 10^2^	4.4844 × 10^2^	6.1179 × 10^2^	4.3973 × 10^2^	4.5903 × 10^2^	4.5120 × 10^2^	4.5584 × 10^2^	7.5087 × 10^3^
	SD	2.0832 × 10^1^	1.1342 × 10^1^	7.9536 × 10^1^	2.1418 × 10^1^	1.1621 × 10^1^	1.8797 × 10^1^	4.6102 × 10^1^	1.8501 × 10^−12^
	Best	4.0000 × 10^2^	4.0681 × 10^2^	4.9015 × 10^2^	4.0000 × 10^2^	4.4909 × 10^2^	4.0987 × 10^2^	4.0408 × 10^2^	7.5087 × 10^3^
	Worst	4.4908 × 10^2^	4.7323 × 10^2^	8.4309 × 10^2^	4.7482 × 10^2^	4.7522 × 10^2^	4.7397 × 10^2^	6.8809 × 10^2^	7.5087 × 10^3^
	Rank	1	7	14	2	13	10	12	16
F3	Mean	6.0000 × 10^2^	6.1033 × 10^2^	6.2428 × 10^2^	6.0543 × 10^2^	6.0387 × 10^2^	6.1900 × 10^2^	6.4403 × 10^2^	7.6031 × 10^2^
	SD	2.2515 × 10^−4^	2.9691 × 10^0^	5.8715 × 10^0^	5.0915 × 10^0^	3.8575 × 10^0^	1.1618 × 10^1^	1.1404 × 10^1^	0.0000 × 10 ^0^
	Best	6.0000 × 10^2^	6.0601 × 10^2^	6.1548 × 10^2^	6.0017 × 10^2^	6.0013 × 10^2^	6.0430 × 10^2^	6.3172 × 10^2^	7.6031 × 10^2^
	Worst	6.0000 × 10^2^	6.1798 × 10^2^	6.3616 × 10^2^	6.1988 × 10^2^	6.1225 × 10^2^	6.4047 × 10^2^	6.7223 × 10^2^	7.6031 × 10^2^
	Rank	1	11	13	10	9	12	14	16
F4	Mean	8.2800 × 10^2^	9.2074 × 10^2^	8.8312 × 10^2^	8.5167 × 10^2^	8.4433 × 10^2^	8.8379 × 10^2^	8.6900 × 10^2^	1.0774 × 10^3^
	SD	9.4366 × 10^0^	1.9229 × 10^1^	1.6695 × 10^1^	1.9919 × 10^1^	7.8835 × 10^0^	1.2805 × 10^1^	1.9026 × 10^1^	4.6252 × 10^−13^
	Best	8.1094 × 10^2^	8.7974 × 10^2^	8.5046 × 10^2^	8.2686 × 10^2^	8.2241 × 10^2^	8.4318 × 10^2^	8.3503 × 10^2^	1.0774 × 10^3^
	Worst	8.4182 × 10^2^	9.5049 × 10^2^	9.1172 × 10^2^	9.0947 × 10^2^	8.6065 × 10^2^	8.9850 × 10^2^	9.1362 × 10^2^	1.0774 × 10^3^
	Rank	2	15	11	9	7	12	10	16
F5	Mean	9.0000 × 10^2^	1.2490 × 10^3^	2.1800 × 10^3^	1.0105 × 10^3^	9.1979 × 10^2^	2.0846 × 10^3^	1.8812 × 10^3^	1.0492 × 10^4^
	SD	2.3313 × 10^−8^	2.8430 × 10^2^	2.4229 × 10^2^	9.6662 × 10^1^	2.9644 × 10^1^	6.5398 × 10^2^	2.8932 × 10^2^	0.0000 × 10 ^0^
	Best	9.0000 × 10^2^	9.6559 × 10^2^	1.6638 × 10^3^	9.0163 × 10^2^	9.0153 × 10^2^	9.2290 × 10^2^	1.3535 × 10^3^	1.0492 × 10^4^
	Worst	9.0000 × 10^2^	2.1094 × 10^3^	2.6660 × 10^3^	1.2271 × 10^3^	1.0256 × 10^3^	2.7421 × 10^3^	2.2770 × 10^3^	1.0492 × 10^4^
	Rank	1	10	14	9	7	13	11	16
F6	Mean	1.8329 × 10^3^	2.1460 × 10^3^	5.2319 × 10^5^	1.8360 × 10^4^	3.9336 × 10^3^	6.0548 × 10^3^	4.7552 × 10^3^	8.8592 × 10^9^
	SD	1.4444 × 10^1^	1.9473 × 10^2^	1.3419 × 10^6^	6.5073 × 10^3^	1.6472 × 10^3^	5.6516 × 10^3^	4.1319 × 10^3^	7.7598 × 10^−6^
	Best	1.8004 × 10^3^	1.9418 × 10^3^	3.3469 × 10^3^	2.2268 × 10^3^	1.9087 × 10^3^	1.9937 × 10^3^	1.9328 × 10^3^	8.8592 × 10^9^
	Worst	1.8700 × 10^3^	2.9174 × 10^3^	4.5566 × 10^6^	2.4890 × 10^4^	8.1378 × 10^3^	2.5122 × 10^4^	1.9787 × 10^4^	8.8592 × 10^9^
	Rank	1	3	15	12	5	9	8	16
F7	Mean	2.0304 × 10^3^	2.0868 × 10^3^	2.0869 × 10^3^	2.0678 × 10^3^	2.0593 × 10^3^	2.0675 × 10^3^	2.0976 × 10^3^	2.6919 × 10^3^
	SD	3.3109 × 10^0^	1.5137 × 10^1^	3.3273 × 10^1^	3.0260 × 10^1^	1.6075 × 10^1^	3.1353 × 10^1^	3.4128 × 10^1^	4.6252 × 10^−13^
	Best	2.0217 × 10^3^	2.0505 × 10^3^	2.0380 × 10^3^	2.0310 × 10^3^	2.0286 × 10^3^	2.0250 × 10^3^	2.0461 × 10^3^	2.6919 × 10^3^
	Worst	2.0362 × 10^3^	2.1139 × 10^3^	2.1500 × 10^3^	2.1549 × 10^3^	2.1016 × 10^3^	2.1819 × 10^3^	2.1958 × 10^3^	2.6919 × 10^3^
	Rank	2	11	12	10	8	9	13	16
F8	Mean	2.2229 × 10^3^	2.2370 × 10^3^	2.2274 × 10^3^	2.2635 × 10^3^	2.2301 × 10^3^	2.2488 × 10^3^	2.2541 × 10^3^	2.2528 × 10^5^
	SD	2.1355 × 10^0^	4.3963 × 10^0^	3.0469 × 10^0^	5.6367 × 10^1^	1.4510 × 10^0^	3.6294 × 10^1^	4.8418 × 10^1^	5.9203 × 10^−11^
	Best	2.2134 × 10^3^	2.2301 × 10^3^	2.2238 × 10^3^	2.2207 × 10^3^	2.2271 × 10^3^	2.2214 × 10^3^	2.2252 × 10^3^	2.2528 × 10^5^
	Worst	2.2243 × 10^3^	2.2479 × 10^3^	2.2405 × 10^3^	2.3462 × 10^3^	2.2328 × 10^3^	2.3556 × 10^3^	2.3490 × 10^3^	2.2528 × 10^5^
	Rank	1	10	6	14	9	12	13	16
F9	Mean	2.4808 × 10^3^	2.4808 × 10^3^	2.5576 × 10^3^	2.4808 × 10^3^	2.4808 × 10^3^	2.4808 × 10^3^	2.4914 × 10^3^	6.6181 × 10^3^
	SD	4.6252 × 10^−13^	4.4287 × 10^−4^	2.9802 × 10^1^	4.6252 × 10^−13^	8.4380 × 10^−8^	4.2771 × 10^−3^	2.6901 × 10^1^	9.2504 × 10^−13^
	Best	2.4808 × 10^3^	2.4808 × 10^3^	2.5226 × 10^3^	2.4808 × 10^3^	2.4808 × 10^3^	2.4808 × 10^3^	2.4808 × 10^3^	6.6181 × 10^3^
	Worst	2.4808 × 10^3^	2.4808 × 10^3^	2.6282 × 10^3^	2.4808 × 10^3^	2.4808 × 10^3^	2.4808 × 10^3^	2.5588 × 10^3^	6.6181 × 10^3^
	Rank	1	8	15	1	7	10	13	16

**Table A10:** Statistical results of the 16 algorithms based on the CEC 2022 benchmark functions (F10 to F12 for the first eight algorithms).

Functions	Index	MSESFOA	SFOA	SHO	FLA	TTAO	COA	BKA	IVY
F10	Mean	2.5005 × 10^3^	2.9645 × 10^3^	2.9565 × 10^3^	3.8669 × 10^3^	2.5101 × 10^3^	3.0083 × 10^3^	3.4151 × 10^3^	1.0921 × 10^4^
	SD	1.0120 × 10^−1^	9.8770 × 10^2^	4.2049 × 10^2^	8.7727 × 10^2^	3.6193 × 10^1^	7.8861 × 10^2^	1.0269 × 10^3^	5.5503 × 10^−12^
	Best	2.5003 × 10^3^	2.5006 × 10^3^	2.5051 × 10^3^	2.5004 × 10^3^	2.5004 × 10^3^	2.5006 × 10^3^	2.5006 × 10^3^	1.0921 × 10^4^
	Worst	2.5007 × 10^3^	5.7880 × 10^3^	3.5981 × 10^3^	5.3509 × 10^3^	2.6445 × 10^3^	5.0825 × 10^3^	5.6130 × 10^3^	1.0921 × 10^4^
	Rank	1	9	8	14	3	10	13	16
F11	Mean	2.9100 × 10^3^	3.2799 × 10^3^	4.9412 × 10^3^	2.9000 × 10^3^	2.9000 × 10^3^	2.9142 × 10^3^	3.1819 × 10^3^	1.0696 × 10^4^
	SD	3.0513 × 10^1^	8.4644 × 10^1^	6.0239 × 10^2^	0.0000 × 10 ^0^	2.2034 × 10^−5^	2.9441 × 10^1^	8.0208 × 10^2^	1.8501 × 10^−12^
	Best	2.9000 × 10^3^	3.0263 × 10^3^	3.9212 × 10^3^	2.9000 × 10^3^	2.9000 × 10^3^	2.9004 × 10^3^	2.9146 × 10^3^	1.0696 × 10^4^
	Worst	3.0000 × 10^3^	3.3432 × 10^3^	6.1632 × 10^3^	2.9000 × 10^3^	2.9000 × 10^3^	3.0003 × 10^3^	6.1277 × 10^3^	1.0696 × 10^4^
	Rank	4	13	15	1	2	9	12	16
F12	Mean	2.9351 × 10^3^	2.9423 × 10^3^	3.0399 × 10^3^	2.9781 × 10^3^	2.9511 × 10^3^	2.9791 × 10^3^	3.0109 × 10^3^	9.2280 × 10^3^
	SD	7.6122 × 10^0^	3.4702 × 10^0^	3.6653 × 10^1^	3.1605 × 10^1^	1.2166 × 10^1^	2.7417 × 10^1^	5.7831 × 10^1^	5.5503 × 10^−12^
	Best	2.9003 × 10^3^	2.9366 × 10^3^	2.9988 × 10^3^	2.9452 × 10^3^	2.9380 × 10^3^	2.9393 × 10^3^	2.9562 × 10^3^	9.2280 × 10^3^
	Worst	2.9439 × 10^3^	2.9492 × 10^3^	3.1136 × 10^3^	3.0751 × 10^3^	2.9859 × 10^3^	3.0232 × 10^3^	3.1590 × 10^3^	9.2280 × 10^3^
	Rank	1	4	15	10	9	11	13	16
Mean rank		1.5000	9.0833	12.6667	7.9167	7.0000	11.0000	11.7500	16.0000
Final rank		1	10	14	9	6	12	13	16

**Table A11:** Statistical results of the 16 algorithms based on the CEC 2022 benchmark functions (F1 to F6 for the second set of eight algorithms).

Functions	Index	NRBO	PKO	EAO	ALA	RIME	IRIME	SAO	ISAO
F1	Mean	6.3210 × 10^3^	9.5540 × 10^3^	3.0000 × 10^2^	3.0210 × 10^2^	3.0225 × 10^2^	3.0005 × 10^2^	6.0853 × 10^3^	3.9336 × 10^3^
	SD	2.2909 × 10^3^	4.4310 × 10^3^	0.0000 × 10 ^0^	1.8744 × 10^0^	1.0508 × 10^0^	3.6960 × 10^−2^	2.9798 × 10^3^	1.9262 × 10^3^
	Best	1.9935 × 10^3^	2.4310 × 10^3^	3.0000 × 10^2^	3.0031 × 10^2^	3.0067 × 10^2^	3.0001 × 10^2^	2.0929 × 10^3^	1.1936 × 10^3^
	Worst	1.2033 × 10^4^	1.6429 × 10^4^	3.0000 × 10^2^	3.0890 × 10^2^	3.0427 × 10^2^	3.0015 × 10^2^	1.5876 × 10^4^	8.8417 × 10^3^
	Rank	12	13	1	6	7	4	11	10
F2	Mean	6.1432 × 10^2^	4.4910 × 10^2^	4.4497 × 10^2^	4.4526 × 10^2^	4.4999 × 10^2^	4.4406 × 10^2^	4.4799 × 10^2^	4.5233 × 10^2^
	SD	7.0899 × 10^1^	1.2098 × 10^−2^	1.2342 × 10^1^	1.0710 × 10^1^	1.6429 × 10^1^	3.3323 × 10^1^	1.3969 × 10^1^	7.3755 × 10^0^
	Best	5.1766 × 10^2^	4.4909 × 10^2^	4.0000 × 10^2^	4.0641 × 10^2^	4.0005 × 10^2^	4.0094 × 10^2^	4.0509 × 10^2^	4.4908 × 10^2^
	Worst	7.5851 × 10^2^	4.4915 × 10^2^	4.4908 × 10^2^	4.4908 × 10^2^	4.7465 × 10^2^	5.3070 × 10^2^	4.6806 × 10^2^	4.6909 × 10^2^
	Rank	15	8	4	5	9	3	6	11
F3	Mean	6.4477 × 10^2^	6.0012 × 10^2^	6.0000 × 10^2^	6.0010 × 10^2^	6.0044 × 10^2^	6.0335 × 10^2^	6.0000 × 10^2^	6.0000 × 10^2^
	SD	9.2734 × 10^0^	9.7997 × 10^−2^	1.8920 × 10^−3^	8.6187 × 10^−2^	4.0276 × 10^−1^	4.7596 × 10^0^	4.5870 × 10^−3^	4.5969 × 10^−4^
	Best	6.3196 × 10^2^	6.0000 × 10^2^	6.0000 × 10^2^	6.0001 × 10^2^	6.0012 × 10^2^	6.0022 × 10^2^	6.0000 × 10^2^	6.0000 × 10^2^
	Worst	6.5584 × 10^2^	6.0036 × 10^2^	6.0001 × 10^2^	6.0034 × 10^2^	6.0152 × 10^2^	6.1661 × 10^2^	6.0002 × 10^2^	6.0000 × 10^2^
	Rank	15	6	3	5	7	8	4	2
F4	Mean	8.9286 × 10^2^	8.2805 × 10^2^	8.2114 × 10^2^	8.4579 × 10^2^	8.4369 × 10^2^	8.9254 × 10^2^	8.2854 × 10^2^	8.2830 × 10^2^
	SD	2.1155 × 10^1^	9.4817 × 10^0^	9.1578 × 10^0^	1.3058 × 10^1^	1.6665 × 10^1^	3.0672 × 10^1^	8.0705 × 10^0^	1.1247 × 10^1^
	Best	8.4560 × 10^2^	8.1396 × 10^2^	8.0796 × 10^2^	8.2288 × 10^2^	8.1993 × 10^2^	8.4278 × 10^2^	8.1492 × 10^2^	8.1592 × 10^2^
	Worst	9.3194 × 10^2^	8.5020 × 10^2^	8.5094 × 10^2^	8.7713 × 10^2^	8.8261 × 10^2^	9.5322 × 10^2^	8.4388 × 10^2^	8.5766 × 10^2^
	Rank	14	3	1	8	6	13	5	4
F5	Mean	1.9804 × 10^3^	9.0018 × 10^2^	9.0008 × 10^2^	9.2229 × 10^2^	9.0480 × 10^2^	2.2754 × 10^3^	9.0049 × 10^2^	9.0001 × 10^2^
	SD	3.5675 × 10^2^	3.7296 × 10^−1^	1.3733 × 10^−1^	4.1721 × 10^1^	6.2709 × 10^0^	1.0660 × 10^3^	9.4323 × 10^−1^	3.0954 × 10^−2^
	Best	1.1356 × 10^3^	9.0000 × 10^2^	9.0000 × 10^2^	9.0091 × 10^2^	9.0011 × 10^2^	9.0969 × 10^2^	9.0000 × 10^2^	9.0000 × 10^2^
	Worst	2.5403 × 10^3^	9.0112 × 10^2^	9.0054 × 10^2^	1.1269 × 10^3^	9.2889 × 10^2^	4.1083 × 10^3^	9.0282 × 10^2^	9.0009 × 10^2^
	Rank	12	4	3	8	6	15	5	2
F6	Mean	4.6359 × 10^4^	4.7219 × 10^4^	1.8409 × 10^3^	3.5972 × 10^3^	7.7708 × 10^3^	1.2915 × 10^4^	4.6648 × 10^3^	4.1955 × 10^3^
	SD	1.7458 × 10^5^	4.0806 × 10^4^	2.6505 × 10^1^	3.1406 × 10^3^	5.3160 × 10^3^	8.4271 × 10^3^	4.7183 × 10^3^	2.6231 × 10^3^
	Best	1.9404 × 10^3^	4.6697 × 10^3^	1.8095 × 10^3^	1.9472 × 10^3^	2.5744 × 10^3^	1.9192 × 10^3^	1.8484 × 10^3^	2.1710 × 10^3^
	Worst	9.6246 × 10^5^	1.5323 × 10^5^	1.9378 × 10^3^	1.4591 × 10^4^	2.0824 × 10^4^	2.5129 × 10^4^	2.0617 × 10^4^	1.3063 × 10^4^
	Rank	13	14	2	4	10	11	7	6

**Table A12:** Statistical results of the 16 algorithms based on the CEC 2022 benchmark functions (F7 to F12 for the second set of eight algorithms).

Functions	Index	NRBO	PKO	EAO	ALA	RIME	IRIME	SAO	ISAO
F7	Mean	2.1442 × 10^3^	2.0444 × 10^3^	2.0298 × 10^3^	2.0438 × 10^3^	2.0334 × 10^3^	2.1029 × 10^3^	2.0442 × 10^3^	2.0391 × 10^3^
	SD	3.6064 × 10^1^	1.0586 × 10^1^	1.5276 × 10^1^	2.5001 × 10^1^	1.0062 × 10^1^	5.7306 × 10^1^	1.7519 × 10^1^	1.4288 × 10^1^
	Best	2.0773 × 10^3^	2.0270 × 10^3^	2.0192 × 10^3^	2.0262 × 10^3^	2.0229 × 10^3^	2.0267 × 10^3^	2.0023 × 10^3^	2.0210 × 10^3^
	Worst	2.2248 × 10^3^	2.0710 × 10^3^	2.0826 × 10^3^	2.1676 × 10^3^	2.0580 × 10^3^	2.3023 × 10^3^	2.0936 × 10^3^	2.0777 × 10^3^
	Rank	15	7	1	5	3	14	6	4
F8	Mean	2.2774 × 10^3^	2.2291 × 10^3^	2.2253 × 10^3^	2.2255 × 10^3^	2.2257 × 10^3^	2.2456 × 10^3^	2.2276 × 10^3^	2.2237 × 10^3^
	SD	5.7758 × 10^1^	2.3377 × 10^0^	6.3168 × 10^0^	2.1874 × 10^0^	2.1523 × 10^0^	4.7238 × 10^1^	2.1976 × 10^1^	6.1825 × 10^0^
	Best	2.2320 × 10^3^	2.2243 × 10^3^	2.2018 × 10^3^	2.2223 × 10^3^	2.2219 × 10^3^	2.2215 × 10^3^	2.2202 × 10^3^	2.2204 × 10^3^
	Worst	2.3742 × 10^3^	2.2329 × 10^3^	2.2294 × 10^3^	2.2308 × 10^3^	2.2296 × 10^3^	2.3592 × 10^3^	2.3391 × 10^3^	2.2376 × 10^3^
	Rank	15	8	3	4	5	11	7	2
F9	Mean	2.5357 × 10^3^	2.4808 × 10^3^	2.4808 × 10^3^	2.4808 × 10^3^	2.4808 × 10^3^	2.4808 × 10^3^	2.4808 × 10^3^	2.4808 × 10^3^
	SD	1.9976 × 10^1^	1.2011 × 10^−3^	4.6252 × 10^−13^	2.2527 × 10^−11^	3.6626 × 10^−2^	5.4817 × 10^−2^	4.6252 × 10^−13^	4.6252 × 10^−13^
	Best	2.5005 × 10^3^	2.4808 × 10^3^	2.4808 × 10^3^	2.4808 × 10^3^	2.4808 × 10^3^	2.4808 × 10^3^	2.4808 × 10^3^	2.4808 × 10^3^
	Worst	2.5751 × 10^3^	2.4808 × 10^3^	2.4808 × 10^3^	2.4808 × 10^3^	2.4809 × 10^3^	2.4810 × 10^3^	2.4808 × 10^3^	2.4808 × 10^3^
	Rank	14	9	1	6	11	12	1	1
F10	Mean	4.4459 × 10^3^	3.3041 × 10^3^	2.5050 × 10^3^	3.1663 × 10^3^	2.5439 × 10^3^	2.7155 × 10^3^	2.6493 × 10^3^	2.5735 × 10^3^
	SD	1.4137 × 10^3^	8.6133 × 10^2^	2.4621 × 10^1^	7.3546 × 10^2^	6.3700 × 10^1^	1.8883 × 10^2^	2.9596 × 10^2^	2.2446 × 10^2^
	Best	2.5010 × 10^3^	2.5004 × 10^3^	2.5003 × 10^3^	2.5003 × 10^3^	2.5003 × 10^3^	2.5003 × 10^3^	2.5004 × 10^3^	2.5004 × 10^3^
	Worst	5.8296 × 10^3^	4.4422 × 10^3^	2.6353 × 10^3^	4.1255 × 10^3^	2.6712 × 10^3^	3.1680 × 10^3^	3.9437 × 10^3^	3.6239 × 10^3^
	Rank	15	12	2	11	4	7	6	5
F11	Mean	4.0035 × 10^3^	2.9002 × 10^3^	2.9200 × 10^3^	2.9100 × 10^3^	2.9358 × 10^3^	2.9125 × 10^3^	2.9100 × 10^3^	2.9100 × 10^3^
	SD	2.5023 × 10^2^	1.2678 × 10^−1^	4.0684 × 10^1^	3.0513 × 10^1^	8.1784 × 10^0^	2.4414 × 10^1^	3.0513 × 10^1^	3.0513 × 10^1^
	Best	3.5403 × 10^3^	2.9000 × 10^3^	2.9000 × 10^3^	2.9000 × 10^3^	2.9228 × 10^3^	2.9003 × 10^3^	2.9000 × 10^3^	2.9000 × 10^3^
	Worst	4.4032 × 10^3^	2.9003 × 10^3^	3.0000 × 10^3^	3.0000 × 10^3^	2.9554 × 10^3^	3.0000 × 10^3^	3.0000 × 10^3^	3.0000 × 10^3^
	Rank	14	3	10	4	11	8	4	4
F12	Mean	3.0235 × 10^3^	2.9406 × 10^3^	2.9387 × 10^3^	2.9478 × 10^3^	2.9473 × 10^3^	2.9964 × 10^3^	2.9448 × 10^3^	2.9460 × 10^3^
	SD	5.3432 × 10^1^	1.6621 × 10^0^	4.7481 × 10^0^	8.5159 × 10^0^	8.4253 × 10^0^	2.5645 × 10^1^	7.8390 × 10^0^	7.3803 × 10^0^
	Best	2.9704 × 10^3^	2.9376 × 10^3^	2.9318 × 10^3^	2.9368 × 10^3^	2.9365 × 10^3^	2.9594 × 10^3^	2.9348 × 10^3^	2.9377 × 10^3^
	Worst	3.1719 × 10^3^	2.9432 × 10^3^	2.9468 × 10^3^	2.9693 × 10^3^	2.9728 × 10^3^	3.0436 × 10^3^	2.9649 × 10^3^	2.9671 × 10^3^
	Rank	14	3	2	8	7	12	5	6
Mean rank		14.0000	7.5000	2.7500	6.1667	7.1667	9.8333	5.5833	4.7500
Final rank		15	8	2	5	7	11	4	3

## Appendix C. The Results of Ablation Study

**Table A13:** Statistical results of the ablation study on CEC2017 (F1 to F10).

Functions	Index	SFOA	SFOA+A	SFOA+M	SFOA+C	MSESFOA
F1	Mean	7.9185 × 10^3^	4.5986 × 10^3^	1.0009 × 10^2^	1.0000 × 10^2^	1.0000 × 10^2^
	SD	6.9407 × 10^3^	7.2756 × 10^3^	9.1402 × 10^−2^	7.1779 × 10^−6^	2.8700 × 10^−7^
	Best	7.0592 × 10^2^	1.0591 × 10^2^	1.0001 × 10^2^	1.0000 × 10^2^	1.0000 × 10^2^
	Worst	2.2361 × 10^4^	2.0936 × 10^4^	1.0048 × 10^2^	1.0000 × 10^2^	1.0000 × 10^2^
	Rank	5	4	3	2	1
F3	Mean	3.0012 × 10^2^	3.0001 × 10^2^	3.0000 × 10^2^	3.0000 × 10^2^	3.0000 × 10^2^
	SD	1.1044 × 10^−1^	1.6519 × 10^−2^	1.7250 × 10^−3^	2.0401 × 10^−7^	4.8754 × 10^−11^
	Best	3.0001 × 10^2^	3.0000 × 10^2^	3.0000 × 10^2^	3.0000 × 10^2^	3.0000 × 10^2^
	Worst	3.0052 × 10^2^	3.0007 × 10^2^	3.0001 × 10^2^	3.0000 × 10^2^	3.0000 × 10^2^
	Rank	5	4	3	2	1
F4	Mean	4.7163 × 10^2^	4.6535 × 10^2^	4.4861 × 10^2^	4.4727 × 10^2^	4.3116 × 10^2^
	SD	2.1732 × 10^1^	2.4308 × 10^1^	2.3408 × 10^1^	2.5728 × 10^1^	3.0968 × 10^1^
	Best	4.0057 × 10^2^	4.0102 × 10^2^	4.0000 × 10^2^	4.0000 × 10^2^	4.0000 × 10^2^
	Worst	4.9023 × 10^2^	4.8799 × 10^2^	4.6412 × 10^2^	4.6790 × 10^2^	4.6412 × 10^2^
	Rank	5	4	3	2	1
F5	Mean	6.2533 × 10^2^	6.1053 × 10^2^	5.5691 × 10^2^	5.6396 × 10^2^	5.3727 × 10^2^
	SD	3.2945 × 10^1^	2.8583 × 10^1^	1.1411 × 10^1^	1.7466 × 10^1^	2.0379 × 10^1^
	Best	5.6651 × 10^2^	5.6760 × 10^2^	5.3200 × 10^2^	5.3582 × 10^2^	5.0149 × 10^2^
	Worst	6.8876 × 10^2^	6.8227 × 10^2^	5.7876 × 10^2^	5.9950 × 10^2^	5.7153 × 10^2^
	Rank	5	4	2	3	1
F6	Mean	6.0491 × 10^2^	6.0489 × 10^2^	6.0002 × 10^2^	6.0000 × 10^2^	6.0000 × 10^2^
	SD	2.4464 × 10^0^	2.1913 × 10^0^	9.3953 × 10^−3^	3.3219 × 10^−11^	5.1358 × 10^−12^
	Best	6.0171 × 10^2^	6.0174 × 10^2^	6.0001 × 10^2^	6.0000 × 10^2^	6.0000 × 10^2^
	Worst	6.1137 × 10^2^	6.1211 × 10^2^	6.0005 × 10^2^	6.0000 × 10^2^	6.0000 × 10^2^
	Rank	5	4	3	2	1
F7	Mean	9.3848 × 10^2^	8.7985 × 10^2^	7.9486 × 10^2^	8.0234 × 10^2^	7.7437 × 10^2^
	SD	2.8192 × 10^1^	4.2683 × 10^1^	1.2639 × 10^1^	2.2947 × 10^1^	2.2429 × 10^1^
	Best	8.7694 × 10^2^	8.1246 × 10^2^	7.7459 × 10^2^	7.7183 × 10^2^	7.2102 × 10^2^
	Worst	9.7780 × 10^2^	9.6392 × 10^2^	8.1733 × 10^2^	8.4701 × 10^2^	7.9550 × 10^2^
	Rank	5	4	2	3	1
F8	Mean	9.4793 × 10^2^	9.0143 × 10^2^	8.5661 × 10^2^	8.7158 × 10^2^	8.6079 × 10^2^
	SD	5.2149 × 10^1^	2.3021 × 10^1^	1.2400 × 10^1^	1.5786 × 10^1^	1.4003 × 10^1^
	Best	8.4562 × 10^2^	8.6463 × 10^2^	8.2702 × 10^2^	8.4179 × 10^2^	8.3651 × 10^2^
	Worst	1.0360 × 10^3^	9.6178 × 10^2^	8.8781 × 10^2^	9.0707 × 10^2^	8.8656 × 10^2^
	Rank	5	4	1	3	2
F9	Mean	1.4601 × 10^3^	1.3998 × 10^3^	9.0059 × 10^2^	9.0031 × 10^2^	9.0000 × 10^2^
	SD	7.0916 × 10^2^	5.1534 × 10^2^	8.9506 × 10^−1^	8.0809 × 10^−1^	0.0000 × 10 ^0^
	Best	9.3309 × 10^2^	9.2176 × 10^2^	9.0000 × 10^2^	9.0000 × 10^2^	9.0000 × 10^2^
	Worst	3.6862 × 10^3^	2.7895 × 10^3^	9.0376 × 10^2^	9.0436 × 10^2^	9.0000 × 10^2^
	Rank	5	4	3	2	1
F10	Mean	7.0490 × 10^3^	7.1048 × 10^3^	3.7479 × 10^3^	3.7217 × 10^3^	3.2489 × 10^3^
	SD	6.9067 × 10^2^	7.7422 × 10^2^	4.3719 × 10^2^	3.9307 × 10^2^	4.2993 × 10^2^
	Best	5.3917 × 10^3^	4.4582 × 10^3^	2.4498 × 10^3^	2.9612 × 10^3^	2.2195 × 10^3^
	Worst	7.9652 × 10^3^	8.1944 × 10^3^	4.3437 × 10^3^	4.5025 × 10^3^	4.1316 × 10^3^
	Rank	4	5	3	2	1

**Table A14:** Statistical results of the ablation study on CEC2017 (F11 to F20).

Functions	Index	SFOA	SFOA+A	SFOA+M	SFOA+C	MSESFOA
F11	Mean	1.1898 × 10^3^	1.1741 × 10^3^	1.1204 × 10^3^	1.1151 × 10^3^	1.1193 × 10^3^
	SD	2.9827 × 10^1^	4.1725 × 10^1^	1.7146 × 10^1^	6.5458 × 10^0^	1.9067 × 10^1^
	Best	1.1258 × 10^3^	1.1198 × 10^3^	1.1053 × 10^3^	1.1061 × 10^3^	1.1010 × 10^3^
	Worst	1.2625 × 10^3^	1.2979 × 10^3^	1.1821 × 10^3^	1.1320 × 10^3^	1.1908 × 10^3^
	Rank	5	4	3	1	2
F12	Mean	2.3425 × 10^4^	1.4706 × 10^4^	2.9464 × 10^3^	5.4725 × 10^3^	1.6474 × 10^3^
	SD	2.4467 × 10^4^	2.7231 × 10^4^	2.6664 × 10^3^	6.7268 × 10^3^	3.3413 × 10^2^
	Best	2.4868 × 10^3^	1.9573 × 10^3^	1.2279 × 10^3^	1.8628 × 10^3^	1.2445 × 10^3^
	Worst	1.0619 × 10^5^	1.4399 × 10^5^	1.5139 × 10^4^	3.0494 × 10^4^	2.9117 × 10^3^
	Rank	5	4	2	3	1
F13	Mean	2.1416 × 10^3^	1.8285 × 10^3^	1.3407 × 10^3^	1.3937 × 10^3^	1.3369 × 10^3^
	SD	9.4720 × 10^2^	4.2331 × 10^2^	7.8961 × 10^0^	6.2280 × 10^1^	1.2308 × 10^1^
	Best	1.4160 × 10^3^	1.4124 × 10^3^	1.3271 × 10^3^	1.3314 × 10^3^	1.3199 × 10^3^
	Worst	4.7927 × 10^3^	2.6695 × 10^3^	1.3593 × 10^3^	1.5721 × 10^3^	1.3683 × 10^3^
	Rank	5	4	2	3	1
F14	Mean	1.4650 × 10^3^	1.4558 × 10^3^	1.4300 × 10^3^	1.4398 × 10^3^	1.4311 × 10^3^
	SD	2.3286 × 10^1^	1.9095 × 10^1^	6.2091 × 10^0^	7.5312 × 10^0^	5.0149 × 10^0^
	Best	1.4407 × 10^3^	1.4309 × 10^3^	1.4101 × 10^3^	1.4296 × 10^3^	1.4094 × 10^3^
	Worst	1.5370 × 10^3^	1.5119 × 10^3^	1.4377 × 10^3^	1.4609 × 10^3^	1.4425 × 10^3^
	Rank	5	4	1	3	2
F15	Mean	1.6391 × 10^3^	1.6416 × 10^3^	1.5151 × 10^3^	1.5362 × 10^3^	1.5127 × 10^3^
	SD	6.7661 × 10^1^	8.0179 × 10^1^	3.9270 × 10^0^	2.8180 × 10^1^	2.6066 × 10^0^
	Best	1.5564 × 10^3^	1.5357 × 10^3^	1.5072 × 10^3^	1.5107 × 10^3^	1.5061 × 10^3^
	Worst	1.8189 × 10^3^	1.8224 × 10^3^	1.5245 × 10^3^	1.5987 × 10^3^	1.5173 × 10^3^
	Rank	4	5	2	3	1
F16	Mean	2.5907 × 10^3^	2.6209 × 10^3^	1.9891 × 10^3^	1.9893 × 10^3^	1.9251 × 10^3^
	SD	3.5885 × 10^2^	3.5331 × 10^2^	1.4690 × 10^2^	1.7314 × 10^2^	1.8669 × 10^2^
	Best	1.9778 × 10^3^	1.9969 × 10^3^	1.6498 × 10^3^	1.6263 × 10^3^	1.6297 × 10^3^
	Worst	3.5528 × 10^3^	3.3307 × 10^3^	2.2515 × 10^3^	2.2691 × 10^3^	2.3053 × 10^3^
	Rank	4	5	2	3	1
F17	Mean	2.0398 × 10^3^	2.0254 × 10^3^	1.7959 × 10^3^	1.8014 × 10^3^	1.7617 × 10^3^
	SD	8.9632 × 10^1^	1.0895 × 10^2^	3.3490 × 10^1^	4.7674 × 10^1^	2.7223 × 10^1^
	Best	1.9053 × 10^3^	1.7846 × 10^3^	1.7484 × 10^3^	1.7357 × 10^3^	1.7332 × 10^3^
	Worst	2.2485 × 10^3^	2.2414 × 10^3^	1.8773 × 10^3^	1.8930 × 10^3^	1.8744 × 10^3^
	Rank	5	4	2	3	1
F18	Mean	1.9537 × 10^3^	1.9359 × 10^3^	1.8301 × 10^3^	1.8581 × 10^3^	1.8273 × 10^3^
	SD	7.1992 × 10^1^	7.8583 × 10^1^	4.3005 × 10^0^	4.4875 × 10^1^	3.7725 × 10^0^
	Best	1.8754 × 10^3^	1.8381 × 10^3^	1.8198 × 10^3^	1.8306 × 10^3^	1.8111 × 10^3^
	Worst	2.1290 × 10^3^	2.1666 × 10^3^	1.8431 × 10^3^	2.0255 × 10^3^	1.8329 × 10^3^
	Rank	5	4	2	3	1
F19	Mean	1.9459 × 10^3^	1.9718 × 10^3^	1.9134 × 10^3^	1.9167 × 10^3^	1.9116 × 10^3^
	SD	3.5982 × 10^1^	5.6020 × 10^1^	2.3985 × 10^0^	3.5367 × 10^0^	2.4247 × 10^0^
	Best	1.9243 × 10^3^	1.9208 × 10^3^	1.9095 × 10^3^	1.9105 × 10^3^	1.9076 × 10^3^
	Worst	2.0788 × 10^3^	2.0950 × 10^3^	1.9182 × 10^3^	1.9240 × 10^3^	1.9159 × 10^3^
	Rank	4	5	2	3	1
F20	Mean	2.3480 × 10^3^	2.3276 × 10^3^	2.0893 × 10^3^	2.0856 × 10^3^	2.0793 × 10^3^
	SD	1.8023 × 10^2^	1.2932 × 10^2^	4.6998 × 10^1^	5.2398 × 10^1^	5.6942 × 10^1^
	Best	2.1617 × 10^3^	2.0911 × 10^3^	2.0364 × 10^3^	2.0366 × 10^3^	2.0031 × 10^3^
	Worst	2.7325 × 10^3^	2.5937 × 10^3^	2.2336 × 10^3^	2.2114 × 10^3^	2.1773 × 10^3^
	Rank	5	4	3	2	1

**Table A15:** Statistical results of the ablation study on CEC2017 (F21 to F30).

Functions	Index	SFOA	SFOA+A	SFOA+M	SFOA+C	MSESFOA
F21	Mean	2.4157 × 10^3^	2.3963 × 10^3^	2.3568 × 10^3^	2.3394 × 10^3^	2.3095 × 10^3^
	SD	3.6642 × 10^1^	2.6973 × 10^1^	1.2043 × 10^1^	6.5483 × 10^1^	7.8386 × 10^1^
	Best	2.3620 × 10^3^	2.3547 × 10^3^	2.3348 × 10^3^	2.2000 × 10^3^	2.1700 × 10^3^
	Worst	2.5045 × 10^3^	2.4800 × 10^3^	2.3818 × 10^3^	2.4025 × 10^3^	2.3897 × 10^3^
	Rank	5	4	3	2	1
F22	Mean	2.3087 × 10^3^	2.3004 × 10^3^	2.3000 × 10^3^	2.3000 × 10^3^	2.3049 × 10^3^
	SD	2.7509 × 10^1^	9.6723 × 10^−1^	5.7922 × 10^−3^	2.2342 × 10^−13^	1.8010 × 10^1^
	Best	2.3001 × 10^3^	2.3000 × 10^3^	2.3000 × 10^3^	2.3000 × 10^3^	2.3000 × 10^3^
	Worst	2.4090 × 10^3^	2.3038 × 10^3^	2.3000 × 10^3^	2.3000 × 10^3^	2.3711 × 10^3^
	Rank	5	3	2	1	4
F23	Mean	2.7696 × 10^3^	2.7495 × 10^3^	2.6987 × 10^3^	2.7138 × 10^3^	2.6609 × 10^3^
	SD	4.1583 × 10^1^	2.6584 × 10^1^	1.1277 × 10^1^	1.9645 × 10^1^	1.1230 × 10^2^
	Best	2.7144 × 10^3^	2.6938 × 10^3^	2.6792 × 10^3^	2.6611 × 10^3^	2.4000 × 10^3^
	Worst	2.8628 × 10^3^	2.8017 × 10^3^	2.7219 × 10^3^	2.7496 × 10^3^	2.7379 × 10^3^
	Rank	5	4	2	3	1
F24	Mean	2.9373 × 10^3^	2.9115 × 10^3^	2.8750 × 10^3^	2.9075 × 10^3^	2.8651 × 10^3^
	SD	2.6994 × 10^1^	1.8054 × 10^1^	1.4837 × 10^1^	2.6710 × 10^1^	5.2617 × 10^1^
	Best	2.8881 × 10^3^	2.8850 × 10^3^	2.8495 × 10^3^	2.8641 × 10^3^	2.6786 × 10^3^
	Worst	2.9950 × 10^3^	2.9553 × 10^3^	2.9118 × 10^3^	2.9687 × 10^3^	2.9341 × 10^3^
	Rank	5	4	2	3	1
F25	Mean	2.8873 × 10^3^	2.8868 × 10^3^	2.8864 × 10^3^	2.8859 × 10^3^	2.8858 × 10^3^
	SD	1.2997 × 10^0^	2.4055 × 10^0^	1.0283 × 10^0^	1.5242 × 10^0^	1.8151 × 10^0^
	Best	2.8835 × 10^3^	2.8834 × 10^3^	2.8834 × 10^3^	2.8834 × 10^3^	2.8800 × 10^3^
	Worst	2.8898 × 10^3^	2.8965 × 10^3^	2.8869 × 10^3^	2.8870 × 10^3^	2.8869 × 10^3^
	Rank	5	4	3	2	1
F26	Mean	3.9823 × 10^3^	3.4492 × 10^3^	3.6166 × 10^3^	3.2720 × 10^3^	3.1915 × 10^3^
	SD	1.0066 × 10^3^	8.1280 × 10^2^	5.6399 × 10^2^	6.3967 × 10^2^	6.0967 × 10^2^
	Best	2.8006 × 10^3^	2.8003 × 10^3^	2.9000 × 10^3^	2.8000 × 10^3^	2.8000 × 10^3^
	Worst	5.4278 × 10^3^	4.9812 × 10^3^	4.2849 × 10^3^	4.5744 × 10^3^	4.5855 × 10^3^
	Rank	5	3	4	2	1
F27	Mean	3.2051 × 10^3^	3.2005 × 10^3^	3.2023 × 10^3^	3.1993 × 10^3^	3.1999 × 10^3^
	SD	9.1934 × 10^0^	1.1222 × 10^1^	1.4472 × 10^1^	1.2772 × 10^1^	1.0128 × 10^1^
	Best	3.1842 × 10^3^	3.1751 × 10^3^	3.1653 × 10^3^	3.1775 × 10^3^	3.1801 × 10^3^
	Worst	3.2212 × 10^3^	3.2212 × 10^3^	3.2258 × 10^3^	3.2318 × 10^3^	3.2193 × 10^3^
	Rank	5	3	4	1	2
F28	Mean	3.2036 × 10^3^	3.1863 × 10^3^	3.1377 × 10^3^	3.1186 × 10^3^	3.1077 × 10^3^
	SD	4.3964 × 10^1^	6.0191 × 10^1^	5.9780 × 10^1^	4.2429 × 10^1^	2.8893 × 10^1^
	Best	3.1148 × 10^3^	3.1001 × 10^3^	3.1000 × 10^3^	3.1000 × 10^3^	3.1000 × 10^3^
	Worst	3.2787 × 10^3^	3.2833 × 10^3^	3.2618 × 10^3^	3.2140 × 10^3^	3.2140 × 10^3^
	Rank	5	4	3	2	1
F29	Mean	3.7194 × 10^3^	3.6718 × 10^3^	3.4332 × 10^3^	3.4544 × 10^3^	3.3862 × 10^3^
	SD	1.5530 × 10^2^	1.8207 × 10^2^	5.9289 × 10^1^	5.8084 × 10^1^	6.6669 × 10^1^
	Best	3.5101 × 10^3^	3.4456 × 10^3^	3.3408 × 10^3^	3.3636 × 10^3^	3.2250 × 10^3^
	Worst	4.0603 × 10^3^	4.1223 × 10^3^	3.5958 × 10^3^	3.5766 × 10^3^	3.5331 × 10^3^
	Rank	5	4	2	3	1
F30	Mean	5.6099 × 10^3^	5.4308 × 10^3^	5.0339 × 10^3^	5.0871 × 10^3^	4.9536 × 10^3^
	SD	9.0575 × 10^2^	1.1878 × 10^3^	1.0301 × 10^2^	1.3867 × 10^2^	1.7379 × 10^2^
	Best	4.9514 × 10^3^	4.9441 × 10^3^	4.9430 × 10^3^	4.9439 × 10^3^	4.0584 × 10^3^
	Worst	9.2566 × 10^3^	1.1529 × 10^4^	5.2848 × 10^3^	5.3763 × 10^3^	5.0905 × 10^3^
	Rank	5	4	2	3	1
Mean rank		4.8621	4.0345	2.4483	2.4138	1.2414
Final rank		5	4	3	2	1

**Table A16:** Statistical results of the ablation study on CEC2022 (F1 to F10).

Functions	Index	SFOA	SFOA+A	SFOA+M	SFOA+C	MSESFOA
F1	Mean	3.3841 × 10^2^	3.0562 × 10^2^	3.0257 × 10^2^	3.0008 × 10^2^	3.0000 × 10^2^
	SD	3.0602 × 10^1^	4.3846 × 10^0^	1.9113 × 10^0^	4.0744 × 10^−2^	4.6488 × 10^−4^
	Best	3.0720 × 10^2^	3.0120 × 10^2^	3.0043 × 10^2^	3.0003 × 10^2^	3.0000 × 10^2^
	Worst	4.6594 × 10^2^	3.2374 × 10^2^	3.0940 × 10^2^	3.0020 × 10^2^	3.0000 × 10^2^
	Rank	5	4	3	2	1
F2	Mean	4.4844 × 10^2^	4.3956 × 10^2^	4.4279 × 10^2^	4.3989 × 10^2^	4.3479 × 10^2^
	SD	1.1342 × 10^1^	1.5514 × 10^1^	1.0994 × 10^1^	1.5602 × 10^1^	2.0832 × 10^1^
	Best	4.0681 × 10^2^	4.0415 × 10^2^	4.0211 × 10^2^	4.0002 × 10^2^	4.0000 × 10^2^
	Worst	4.7323 × 10^2^	4.4955 × 10^2^	4.4908 × 10^2^	4.4908 × 10^2^	4.4908 × 10^2^
	Rank	5	2	4	3	1
F3	Mean	6.1033 × 10^2^	6.1014 × 10^2^	6.0038 × 10^2^	6.0000 × 10^2^	6.0000 × 10^2^
	SD	2.9691 × 10^0^	3.6669 × 10^0^	1.1698 × 10^−1^	1.6921 × 10^−4^	2.2515 × 10^−4^
	Best	6.0601 × 10^2^	6.0514 × 10^2^	6.0018 × 10^2^	6.0000 × 10^2^	6.0000 × 10^2^
	Worst	6.1798 × 10^2^	6.1984 × 10^2^	6.0063 × 10^2^	6.0000 × 10^2^	6.0000 × 10^2^
	Rank	5	4	3	2	1
F4	Mean	9.2074 × 10^2^	9.1386 × 10^2^	8.4468 × 10^2^	8.4528 × 10^2^	8.2800 × 10^2^
	SD	1.9229 × 10^1^	1.7310 × 10^1^	1.1192 × 10^1^	1.2012 × 10^1^	9.4366 × 10^0^
	Best	8.7974 × 10^2^	8.8129 × 10^2^	8.2593 × 10^2^	8.2213 × 10^2^	8.1094 × 10^2^
	Worst	9.5049 × 10^2^	9.4672 × 10^2^	8.6777 × 10^2^	8.7269 × 10^2^	8.4182 × 10^2^
	Rank	5	4	2	3	1
F5	Mean	1.2490 × 10^3^	1.0746 × 10^3^	9.0722 × 10^2^	9.0017 × 10^2^	9.0000 × 10^2^
	SD	2.8430 × 10^2^	1.2809 × 10^2^	5.0899 × 10^0^	5.7045 × 10^−1^	2.3313 × 10^−8^
	Best	9.6559 × 10^2^	9.1718 × 10^2^	9.0039 × 10^2^	9.0000 × 10^2^	9.0000 × 10^2^
	Worst	2.1094 × 10^3^	1.4113 × 10^3^	9.2553 × 10^2^	9.0272 × 10^2^	9.0000 × 10^2^
	Rank	5	4	3	2	1
F6	Mean	2.1460 × 10^3^	2.0172 × 10^3^	1.8677 × 10^3^	1.9841 × 10^3^	1.8329 × 10^3^
	SD	1.9473 × 10^2^	8.5465 × 10^1^	2.7628 × 10^1^	1.0290 × 10^2^	1.4444 × 10^1^
	Best	1.9418 × 10^3^	1.8813 × 10^3^	1.8297 × 10^3^	1.8500 × 10^3^	1.8004 × 10^3^
	Worst	2.9174 × 10^3^	2.2251 × 10^3^	1.9768 × 10^3^	2.3217 × 10^3^	1.8700 × 10^3^
	Rank	5	4	2	3	1
F7	Mean	2.0868 × 10^3^	2.0814 × 10^3^	2.0355 × 10^3^	2.0341 × 10^3^	2.0304 × 10^3^
	SD	1.5137 × 10^1^	1.4429 × 10^1^	4.7657 × 10^0^	4.7858 × 10^0^	3.3109 × 10^0^
	Best	2.0505 × 10^3^	2.0544 × 10^3^	2.0274 × 10^3^	2.0259 × 10^3^	2.0217 × 10^3^
	Worst	2.1139 × 10^3^	2.1061 × 10^3^	2.0455 × 10^3^	2.0488 × 10^3^	2.0362 × 10^3^
	Rank	5	4	3	2	1
F8	Mean	2.2370 × 10^3^	2.2372 × 10^3^	2.2254 × 10^3^	2.2245 × 10^3^	2.2229 × 10^3^
	SD	4.3963 × 10^0^	3.3754 × 10^0^	7.8076 × 10^−1^	9.9243 × 10^−1^	2.1355 × 10^0^
	Best	2.2301 × 10^3^	2.2295 × 10^3^	2.2239 × 10^3^	2.2205 × 10^3^	2.2134 × 10^3^
	Worst	2.2479 × 10^3^	2.2430 × 10^3^	2.2267 × 10^3^	2.2265 × 10^3^	2.2243 × 10^3^
	Rank	4	5	3	2	1
F9	Mean	2.4808 × 10^3^	2.4808 × 10^3^	2.4808 × 10^3^	2.4808 × 10^3^	2.4808 × 10^3^
	SD	4.4287 × 10^−4^	9.3154 × 10^−5^	2.2957 × 10^−6^	6.8603 × 10^−13^	0.0000 × 10 ^0^
	Best	2.4808 × 10^3^	2.4808 × 10^3^	2.4808 × 10^3^	2.4808 × 10^3^	2.4808 × 10^3^
	Worst	2.4808 × 10^3^	2.4808 × 10^3^	2.4808 × 10^3^	2.4808 × 10^3^	2.4808 × 10^3^
	Rank	5	4	3	2	1
F10	Mean	2.9645 × 10^3^	2.6016 × 10^3^	2.5379 × 10^3^	2.5005 × 10^3^	2.5005 × 10^3^
	SD	9.8770 × 10^2^	5.2266 × 10^2^	6.2931 × 10^1^	7.4423 × 10^−2^	1.0120 × 10^−1^
	Best	2.5006 × 10^3^	2.5004 × 10^3^	2.5005 × 10^3^	2.5004 × 10^3^	2.5003 × 10^3^
	Worst	5.7880 × 10^3^	5.3644 × 10^3^	2.6491 × 10^3^	2.5007 × 10^3^	2.5007 × 10^3^
	Rank	5	4	3	2	1

**Table A17:** Statistical results of the ablation study on CEC2022 (F11 to F12).

Functions	Index	SFOA	SFOA+A	SFOA+M	SFOA+C	MSESFOA
F11	Mean	3.2799 × 10^3^	3.1389 × 10^3^	2.9713 × 10^3^	2.9600 × 10^3^	**2.9100 × 10^3^**
	SD	8.4644 × 10^1^	1.4819 × 10^2^	5.1879 × 10^1^	4.9827 × 10^1^	3.0513 × 10^1^
	Best	3.0263 × 10^3^	2.6886 × 10^3^	2.9030 × 10^3^	2.9000 × 10^3^	2.9000 × 10^3^
	Worst	3.3432 × 10^3^	3.3078 × 10^3^	3.0269 × 10^3^	3.0000 × 10^3^	3.0000 × 10^3^
	Rank	5	4	3	2	1
F12	Mean	2.9423 × 10^3^	2.9398 × 10^3^	2.9394 × 10^3^	2.9376 × 10^3^	**2.9351 × 10^3^**
	SD	3.4702 × 10^0^	3.7352 × 10^0^	4.4202 × 10^0^	3.2527 × 10^0^	7.6122 × 10^0^
	Best	2.9366 × 10^3^	2.9342 × 10^3^	2.9319 × 10^3^	2.9327 × 10^3^	2.9003 × 10^3^
	Worst	2.9492 × 10^3^	2.9481 × 10^3^	2.9488 × 10^3^	2.9443 × 10^3^	2.9439 × 10^3^
	Rank	5	4	3	2	1
Mean rank		4.9167	3.9167	2.9167	2.2500	1.0000
Final rank		5	4	3	2	1

## Appendix D. The Results of Sensitivity Analysis

**Table A18:** Statistical results of the parameter sensitivity analysis on CEC2017 (F1 to F13).

Functions	Index	Default	P-Low	P-High	F-Low	F-High	Stage-60	Stage-80
F1	Mean	1.0000 × 10^2^	1.0000 × 10^2^	1.0000 × 10^2^	1.0000 × 10^2^	1.0000 × 10^2^	1.0000 × 10^2^	1.0000 × 10^2^
	SD	2.8700 × 10^−7^	4.5209 × 10^−7^	1.3939 × 10^−7^	1.5913 × 10^−7^	9.2471 × 10^−7^	2.8266 × 10^−7^	3.5529 × 10^−7^
	Best	1.0000 × 10^2^	1.0000 × 10^2^	1.0000 × 10^2^	1.0000 × 10^2^	1.0000 × 10^2^	1.0000 × 10^2^	1.0000 × 10^2^
	Worst	1.0000 × 10^2^	1.0000 × 10^2^	1.0000 × 10^2^	1.0000 × 10^2^	1.0000 × 10^2^	1.0000 × 10^2^	1.0000 × 10^2^
F3	Mean	3.0000 × 10^2^	3.0000 × 10^2^	3.0000 × 10^2^	3.0000 × 10^2^	3.0000 × 10^2^	3.0000 × 10^2^	3.0000 × 10^2^
	SD	4.8754 × 10^−11^	1.2784 × 10^−10^	1.1489 × 10^−11^	3.2705 × 10^−13^	2.0053 × 10^−10^	5.6355 × 10^−11^	2.2266 × 10^−11^
	Best	3.0000 × 10^2^	3.0000 × 10^2^	3.0000 × 10^2^	3.0000 × 10^2^	3.0000 × 10^2^	3.0000 × 10^2^	3.0000 × 10^2^
	Worst	3.0000 × 10^2^	3.0000 × 10^2^	3.0000 × 10^2^	3.0000 × 10^2^	3.0000 × 10^2^	3.0000 × 10^2^	3.0000 × 10^2^
F4	Mean	4.3116 × 10^2^	4.4329 × 10^2^	4.5434 × 10^2^	4.4264 × 10^2^	4.3966 × 10^2^	4.4400 × 10^2^	4.4400 × 10^2^
	SD	3.0968 × 10^1^	2.7599 × 10^1^	1.7796 × 10^1^	2.7315 × 10^1^	2.9676 × 10^1^	2.6769 × 10^1^	2.6769 × 10^1^
	Best	4.0000 × 10^2^	4.0000 × 10^2^	4.0000 × 10^2^	4.0000 × 10^2^	4.0000 × 10^2^	4.0000 × 10^2^	4.0000 × 10^2^
	Worst	4.6412 × 10^2^	4.7189 × 10^2^	4.7189 × 10^2^	4.6412 × 10^2^	4.6412 × 10^2^	4.6412 × 10^2^	4.6412 × 10^2^
F5	Mean	5.3727 × 10^2^	5.5813 × 10^2^	5.5669 × 10^2^	5.5032 × 10^2^	5.5601 × 10^2^	5.6161 × 10^2^	5.6022 × 10^2^
	SD	2.0379 × 10^1^	1.2455 × 10^1^	1.8460 × 10^1^	1.2045 × 10^1^	1.5817 × 10^1^	2.1272 × 10^1^	2.1262 × 10^1^
	Best	5.0149 × 10^2^	5.3594 × 10^2^	5.2587 × 10^2^	5.2885 × 10^2^	5.2392 × 10^2^	5.2612 × 10^2^	5.2688 × 10^2^
	Worst	5.7153 × 10^2^	5.8465 × 10^2^	6.0447 × 10^2^	5.7064 × 10^2^	5.9452 × 10^2^	6.0845 × 10^2^	6.0845 × 10^2^
F6	Mean	6.0000 × 10^2^	6.0000 × 10^2^	6.0000 × 10^2^	6.0000 × 10^2^	6.0000 × 10^2^	6.0000 × 10^2^	6.0000 × 10^2^
	SD	5.1358 × 10^−12^	2.0035 × 10^−7^	5.0691 × 10^−11^	2.5001 × 10^−8^	4.7582 × 10^−8^	1.0043 × 10^−7^	1.0473 × 10^−7^
	Best	6.0000 × 10^2^	6.0000 × 10^2^	6.0000 × 10^2^	6.0000 × 10^2^	6.0000 × 10^2^	6.0000 × 10^2^	6.0000 × 10^2^
	Worst	6.0000 × 10^2^	6.0000 × 10^2^	6.0000 × 10^2^	6.0000 × 10^2^	6.0000 × 10^2^	6.0000 × 10^2^	6.0000 × 10^2^
F7	Mean	7.7437 × 10^2^	7.8319 × 10^2^	7.9040 × 10^2^	7.8201 × 10^2^	7.8867 × 10^2^	7.8569 × 10^2^	7.8345 × 10^2^
	SD	2.2429 × 10^1^	1.3508 × 10^1^	1.8886 × 10^1^	1.6971 × 10^1^	1.6372 × 10^1^	1.6696 × 10^1^	1.6488 × 10^1^
	Best	7.2102 × 10^2^	7.5247 × 10^2^	7.6775 × 10^2^	7.5730 × 10^2^	7.6373 × 10^2^	7.5783 × 10^2^	7.5805 × 10^2^
	Worst	7.9550 × 10^2^	8.0373 × 10^2^	8.4010 × 10^2^	8.2585 × 10^2^	8.3607 × 10^2^	8.1999 × 10^2^	8.1739 × 10^2^
F8	Mean	8.6079 × 10^2^	8.5707 × 10^2^	8.6247 × 10^2^	8.6620 × 10^2^	8.6079 × 10^2^	8.6016 × 10^2^	8.5878 × 10^2^
	SD	1.4003 × 10^1^	1.8169 × 10^1^	1.8199 × 10^1^	1.7317 × 10^1^	1.4814 × 10^1^	1.4281 × 10^1^	1.4888 × 10^1^
	Best	8.3651 × 10^2^	8.3084 × 10^2^	8.2885 × 10^2^	8.3489 × 10^2^	8.3753 × 10^2^	8.3781 × 10^2^	8.3152 × 10^2^
	Worst	8.8656 × 10^2^	9.0149 × 10^2^	8.9950 × 10^2^	9.0187 × 10^2^	8.9452 × 10^2^	8.9136 × 10^2^	8.8760 × 10^2^
F9	Mean	9.0000 × 10^2^	9.0008 × 10^2^	9.0006 × 10^2^	9.0003 × 10^2^	9.0000 × 10^2^	9.0003 × 10^2^	9.0003 × 10^2^
	SD	0.0000 × 10 ^0^	1.7961 × 10^−1^	1.5708 × 10^−1^	1.1527 × 10^−1^	1.6346 × 10^−2^	1.6614 × 10^−1^	1.6614 × 10^−1^
	Best	9.0000 × 10^2^	9.0000 × 10^2^	9.0000 × 10^2^	9.0000 × 10^2^	9.0000 × 10^2^	9.0000 × 10^2^	9.0000 × 10^2^
	Worst	9.0000 × 10^2^	9.0054 × 10^2^	9.0045 × 10^2^	9.0045 × 10^2^	9.0009 × 10^2^	9.0091 × 10^2^	9.0091 × 10^2^
F10	Mean	3.2489 × 10^3^	3.5854 × 10^3^	3.3091 × 10^3^	3.3970 × 10^3^	3.3823 × 10^3^	3.3884 × 10^3^	3.3527 × 10^3^
	SD	4.2993 × 10^2^	3.4067 × 10^2^	3.8123 × 10^2^	3.5541 × 10^2^	2.8198 × 10^2^	3.8046 × 10^2^	3.8351 × 10^2^
	Best	2.2195 × 10^3^	2.9745 × 10^3^	2.6195 × 10^3^	2.7238 × 10^3^	2.7504 × 10^3^	2.2757 × 10^3^	2.2648 × 10^3^
	Worst	4.1316 × 10^3^	4.4878 × 10^3^	3.9028 × 10^3^	4.1471 × 10^3^	3.8386 × 10^3^	4.0273 × 10^3^	3.9269 × 10^3^
F11	Mean	1.1193 × 10^3^	1.1163 × 10^3^	1.1150 × 10^3^	1.1159 × 10^3^	1.1159 × 10^3^	1.1195 × 10^3^	1.1171 × 10^3^
	SD	1.9067 × 10^1^	1.6823 × 10^1^	6.8224 × 10^0^	1.2348 × 10^1^	1.6269 × 10^1^	1.5263 × 10^1^	1.2377 × 10^1^
	Best	1.1010 × 10^3^	1.1031 × 10^3^	1.1050 × 10^3^	1.1041 × 10^3^	1.1030 × 10^3^	1.1060 × 10^3^	1.1051 × 10^3^
	Worst	1.1908 × 10^3^	1.1779 × 10^3^	1.1279 × 10^3^	1.1709 × 10^3^	1.1947 × 10^3^	1.1699 × 10^3^	1.1699 × 10^3^
F12	Mean	1.6474 × 10^3^	3.1976 × 10^3^	1.6012 × 10^3^	1.7795 × 10^3^	1.8103 × 10^3^	1.8232 × 10^3^	1.9630 × 10^3^
	SD	3.3413 × 10^2^	7.4718 × 10^3^	4.6846 × 10^2^	6.1654 × 10^2^	8.1654 × 10^2^	6.8051 × 10^2^	1.2692 × 10^3^
	Best	1.2445 × 10^3^	1.2358 × 10^3^	1.2089 × 10^3^	1.2125 × 10^3^	1.3505 × 10^3^	1.3360 × 10^3^	1.3364 × 10^3^
	Worst	2.9117 × 10^3^	4.2589 × 10^4^	3.8222 × 10^3^	3.4665 × 10^3^	5.6861 × 10^3^	4.7689 × 10^3^	7.7755 × 10^3^
F13	Mean	1.3369 × 10^3^	1.3383 × 10^3^	1.3310 × 10^3^	1.3314 × 10^3^	1.3356 × 10^3^	1.3387 × 10^3^	1.3394 × 10^3^
	SD	1.2308 × 10^1^	2.2174 × 10^1^	9.4829 × 10^0^	1.3540 × 10^1^	1.4123 × 10^1^	1.8742 × 10^1^	2.1097 × 10^1^
	Best	1.3199 × 10^3^	1.3060 × 10^3^	1.3131 × 10^3^	1.3090 × 10^3^	1.3141 × 10^3^	1.3175 × 10^3^	1.3167 × 10^3^
	Worst	1.3683 × 10^3^	1.4313 × 10^3^	1.3539 × 10^3^	1.3876 × 10^3^	1.3854 × 10^3^	1.4150 × 10^3^	1.4034 × 10^3^

**Table A19:** Statistical results of the parameter sensitivity analysis on CEC2017 (F14 to F26).

Functions	Index	Default	P-Low	P-High	F-Low	F-High	Stage-60	Stage-80
F14	Mean	1.4311 × 10^3^	1.4314 × 10^3^	1.4299 × 10^3^	1.4308 × 10^3^	1.4301 × 10^3^	1.4307 × 10^3^	1.4303 × 10^3^
	SD	5.0149 × 10^0^	7.9687 × 10^0^	5.5493 × 10^0^	5.9007 × 10^0^	9.1331 × 10^0^	5.7327 × 10^0^	5.8898 × 10^0^
	Best	1.4094 × 10^3^	1.4071 × 10^3^	1.4141 × 10^3^	1.4080 × 10^3^	1.4062 × 10^3^	1.4136 × 10^3^	1.4134 × 10^3^
	Worst	1.4425 × 10^3^	1.4410 × 10^3^	1.4391 × 10^3^	1.4391 × 10^3^	1.4454 × 10^3^	1.4382 × 10^3^	1.4404 × 10^3^
F15	Mean	1.5127 × 10^3^	1.5131 × 10^3^	1.5132 × 10^3^	1.5109 × 10^3^	1.5124 × 10^3^	1.5130 × 10^3^	1.5130 × 10^3^
	SD	2.6066 × 10^0^	3.6921 × 10^0^	3.3216 × 10^0^	3.6932 × 10^0^	3.1630 × 10^0^	3.4692 × 10^0^	3.0811 × 10^0^
	Best	1.5061 × 10^3^	1.5057 × 10^3^	1.5075 × 10^3^	1.5040 × 10^3^	1.5037 × 10^3^	1.5076 × 10^3^	1.5071 × 10^3^
	Worst	1.5173 × 10^3^	1.5241 × 10^3^	1.5216 × 10^3^	1.5182 × 10^3^	1.5205 × 10^3^	1.5202 × 10^3^	1.5204 × 10^3^
F16	Mean	1.9251 × 10^3^	2.0309 × 10^3^	1.9910 × 10^3^	1.9225 × 10^3^	2.0541 × 10^3^	2.0003 × 10^3^	1.9988 × 10^3^
	SD	1.8669 × 10^2^	1.8797 × 10^2^	1.5425 × 10^2^	2.0767 × 10^2^	1.8465 × 10^2^	1.4875 × 10^2^	1.5024 × 10^2^
	Best	1.6297 × 10^3^	1.6361 × 10^3^	1.6614 × 10^3^	1.6251 × 10^3^	1.7672 × 10^3^	1.6258 × 10^3^	1.6261 × 10^3^
	Worst	2.3053 × 10^3^	2.3669 × 10^3^	2.3175 × 10^3^	2.4503 × 10^3^	2.4867 × 10^3^	2.2510 × 10^3^	2.2509 × 10^3^
F17	Mean	1.7617 × 10^3^	1.7923 × 10^3^	1.7704 × 10^3^	1.7895 × 10^3^	1.7822 × 10^3^	1.7680 × 10^3^	1.7663 × 10^3^
	SD	2.7223 × 10^1^	4.4461 × 10^1^	3.6220 × 10^1^	5.0432 × 10^1^	4.6132 × 10^1^	2.8352 × 10^1^	2.7623 × 10^1^
	Best	1.7332 × 10^3^	1.7285 × 10^3^	1.7382 × 10^3^	1.7302 × 10^3^	1.7312 × 10^3^	1.7366 × 10^3^	1.7365 × 10^3^
	Worst	1.8744 × 10^3^	1.8941 × 10^3^	1.8826 × 10^3^	1.9071 × 10^3^	1.9109 × 10^3^	1.8669 × 10^3^	1.8671 × 10^3^
F18	Mean	1.8273 × 10^3^	1.8286 × 10^3^	1.8270 × 10^3^	1.8275 × 10^3^	1.8284 × 10^3^	1.8275 × 10^3^	1.8274 × 10^3^
	SD	3.7725 × 10^0^	3.2517 × 10^0^	3.7878 × 10^0^	2.4410 × 10^0^	4.2818 × 10^0^	4.4512 × 10^0^	4.3252 × 10^0^
	Best	1.8111 × 10^3^	1.8244 × 10^3^	1.8094 × 10^3^	1.8221 × 10^3^	1.8108 × 10^3^	1.8126 × 10^3^	1.8089 × 10^3^
	Worst	1.8329 × 10^3^	1.8410 × 10^3^	1.8325 × 10^3^	1.8329 × 10^3^	1.8355 × 10^3^	1.8356 × 10^3^	1.8335 × 10^3^
F19	Mean	1.9116 × 10^3^	1.9138 × 10^3^	1.9128 × 10^3^	1.9118 × 10^3^	1.9138 × 10^3^	1.9129 × 10^3^	1.9124 × 10^3^
	SD	2.4247 × 10^0^	2.8697 × 10^0^	2.9186 × 10^0^	2.3894 × 10^0^	3.0509 × 10^0^	3.3273 × 10^0^	3.1174 × 10^0^
	Best	1.9076 × 10^3^	1.9082 × 10^3^	1.9069 × 10^3^	1.9076 × 10^3^	1.9077 × 10^3^	1.9082 × 10^3^	1.9074 × 10^3^
	Worst	1.9159 × 10^3^	1.9182 × 10^3^	1.9185 × 10^3^	1.9164 × 10^3^	1.9186 × 10^3^	1.9231 × 10^3^	1.9215 × 10^3^
F20	Mean	2.0793 × 10^3^	2.0811 × 10^3^	2.0687 × 10^3^	2.0810 × 10^3^	2.0665 × 10^3^	2.0819 × 10^3^	2.0776 × 10^3^
	SD	5.6942 × 10^1^	4.6785 × 10^1^	5.1680 × 10^1^	4.9688 × 10^1^	4.8891 × 10^1^	5.8864 × 10^1^	5.6224 × 10^1^
	Best	2.0031 × 10^3^	2.0323 × 10^3^	2.0199 × 10^3^	2.0224 × 10^3^	2.0283 × 10^3^	2.0340 × 10^3^	2.0324 × 10^3^
	Worst	2.1773 × 10^3^	2.1885 × 10^3^	2.1621 × 10^3^	2.1673 × 10^3^	2.1800 × 10^3^	2.2482 × 10^3^	2.2483 × 10^3^
F21	Mean	2.3095 × 10^3^	2.3381 × 10^3^	2.3645 × 10^3^	2.3333 × 10^3^	2.3431 × 10^3^	2.3376 × 10^3^	2.3362 × 10^3^
	SD	7.8386 × 10^1^	5.6632 × 10^1^	3.9149 × 10^1^	5.6385 × 10^1^	4.2165 × 10^1^	5.6565 × 10^1^	5.6132 × 10^1^
	Best	2.1700 × 10^3^	2.2000 × 10^3^	2.2000 × 10^3^	2.2000 × 10^3^	2.2000 × 10^3^	2.2000 × 10^3^	2.2000 × 10^3^
	Worst	2.3897 × 10^3^	2.3842 × 10^3^	2.4282 × 10^3^	2.4128 × 10^3^	2.3899 × 10^3^	2.3891 × 10^3^	2.3891 × 10^3^
F22	Mean	2.3049 × 10^3^	2.3000 × 10^3^	2.3001 × 10^3^	2.3000 × 10^3^	2.3000 × 10^3^	2.3001 × 10^3^	2.3001 × 10^3^
	SD	1.8010 × 10^1^	1.8882 × 10^−13^	4.4792 × 10^−1^	2.0685 × 10^−13^	1.6889 × 10^−13^	4.4909 × 10^−1^	4.4909 × 10^−1^
	Best	2.3000 × 10^3^	2.3000 × 10^3^	2.3000 × 10^3^	2.3000 × 10^3^	2.3000 × 10^3^	2.3000 × 10^3^	2.3000 × 10^3^
	Worst	2.3711 × 10^3^	2.3000 × 10^3^	2.3025 × 10^3^	2.3000 × 10^3^	2.3000 × 10^3^	2.3025 × 10^3^	2.3025 × 10^3^
F23	Mean	2.6609 × 10^3^	2.7079 × 10^3^	2.7094 × 10^3^	2.7064 × 10^3^	2.7072 × 10^3^	2.7082 × 10^3^	2.7075 × 10^3^
	SD	1.1230 × 10^2^	1.6003 × 10^1^	1.6284 × 10^1^	8.6237 × 10^0^	1.6267 × 10^1^	1.7818 × 10^1^	1.8528 × 10^1^
	Best	2.4000 × 10^3^	2.6790 × 10^3^	2.6762 × 10^3^	2.6905 × 10^3^	2.6834 × 10^3^	2.6745 × 10^3^	2.6745 × 10^3^
	Worst	2.7379 × 10^3^	2.7421 × 10^3^	2.7428 × 10^3^	2.7224 × 10^3^	2.7496 × 10^3^	2.7643 × 10^3^	2.7643 × 10^3^
F24	Mean	2.8651 × 10^3^	2.8847 × 10^3^	2.8811 × 10^3^	2.8820 × 10^3^	2.8860 × 10^3^	2.8859 × 10^3^	2.8859 × 10^3^
	SD	5.2617 × 10^1^	1.4154 × 10^1^	1.5526 × 10^1^	1.7671 × 10^1^	1.6468 × 10^1^	1.5052 × 10^1^	1.5052 × 10^1^
	Best	2.6786 × 10^3^	2.8635 × 10^3^	2.8549 × 10^3^	2.8502 × 10^3^	2.8572 × 10^3^	2.8561 × 10^3^	2.8561 × 10^3^
	Worst	2.9341 × 10^3^	2.9259 × 10^3^	2.9170 × 10^3^	2.9188 × 10^3^	2.9348 × 10^3^	2.9178 × 10^3^	2.9178 × 10^3^
F25	Mean	2.8858 × 10^3^	2.8863 × 10^3^	2.8859 × 10^3^	2.8865 × 10^3^	2.8865 × 10^3^	2.8866 × 10^3^	2.8866 × 10^3^
	SD	1.8151 × 10^0^	1.2900 × 10^0^	1.5253 × 10^0^	1.0409 × 10^0^	1.0280 × 10^0^	8.6045 × 10^−1^	8.6045 × 10^−1^
	Best	2.8800 × 10^3^	2.8834 × 10^3^	2.8834 × 10^3^	2.8834 × 10^3^	2.8834 × 10^3^	2.8834 × 10^3^	2.8834 × 10^3^
	Worst	2.8869 × 10^3^	2.8870 × 10^3^	2.8870 × 10^3^	2.8869 × 10^3^	2.8869 × 10^3^	2.8869 × 10^3^	2.8869 × 10^3^
F26	Mean	3.1915 × 10^3^	3.0973 × 10^3^	3.2180 × 10^3^	3.1435 × 10^3^	3.2531 × 10^3^	3.2179 × 10^3^	3.2150 × 10^3^
	SD	6.0967 × 10^2^	4.6042 × 10^2^	5.9974 × 10^2^	5.1717 × 10^2^	6.4083 × 10^2^	6.1642 × 10^2^	6.1159 × 10^2^
	Best	2.8000 × 10^3^	2.8000 × 10^3^	2.8000 × 10^3^	2.8000 × 10^3^	2.8000 × 10^3^	2.8000 × 10^3^	2.8000 × 10^3^
	Worst	4.5855 × 10^3^	4.2594 × 10^3^	4.4633 × 10^3^	4.3652 × 10^3^	4.7330 × 10^3^	4.8545 × 10^3^	4.8545 × 10^3^

**Table A20:** Statistical results of the parameter sensitivity analysis on CEC2017 (F27 to F30).

Functions	Index	Default	P-Low	P-High	F-Low	F-High	Stage-60	Stage-80
F27	Mean	3.1999 × 10^3^	3.1936 × 10^3^	3.1943 × 10^3^	3.1981 × 10^3^	3.1952 × 10^3^	3.1922 × 10^3^	3.1921 × 10^3^
	SD	1.0128 × 10^1^	1.1230 × 10^1^	1.0728 × 10^1^	1.0641 × 10^1^	9.2239 × 10^0^	8.1950 × 10^0^	8.1313 × 10^0^
	Best	3.1801 × 10^3^	3.1640 × 10^3^	3.1671 × 10^3^	3.1608 × 10^3^	3.1724 × 10^3^	3.1713 × 10^3^	3.1713 × 10^3^
	Worst	3.2193 × 10^3^	3.2128 × 10^3^	3.2119 × 10^3^	3.2164 × 10^3^	3.2149 × 10^3^	3.2077 × 10^3^	3.2077 × 10^3^
F28	Mean	3.1077 × 10^3^	3.1426 × 10^3^	3.1148 × 10^3^	3.1386 × 10^3^	3.1261 × 10^3^	3.1283 × 10^3^	3.1283 × 10^3^
	SD	2.8893 × 10^1^	5.8005 × 10^1^	3.8521 × 10^1^	5.1639 × 10^1^	4.8763 × 10^1^	4.7719 × 10^1^	4.7719 × 10^1^
	Best	3.1000 × 10^3^	3.1000 × 10^3^	3.1000 × 10^3^	3.1000 × 10^3^	3.1000 × 10^3^	3.1000 × 10^3^	3.1000 × 10^3^
	Worst	3.2140 × 10^3^	3.2539 × 10^3^	3.2140 × 10^3^	3.2140 × 10^3^	3.2517 × 10^3^	3.2140 × 10^3^	3.2140 × 10^3^
F29	Mean	3.3862 × 10^3^	3.4141 × 10^3^	3.3861 × 10^3^	3.4080 × 10^3^	3.4114 × 10^3^	3.3926 × 10^3^	3.3877 × 10^3^
	SD	6.6669 × 10^1^	5.3343 × 10^1^	5.0896 × 10^1^	5.0058 × 10^1^	5.3125 × 10^1^	5.7513 × 10^1^	5.6636 × 10^1^
	Best	3.2250 × 10^3^	3.2956 × 10^3^	3.3044 × 10^3^	3.3280 × 10^3^	3.3244 × 10^3^	3.2836 × 10^3^	3.2817 × 10^3^
	Worst	3.5331 × 10^3^	3.5271 × 10^3^	3.5151 × 10^3^	3.5130 × 10^3^	3.5383 × 10^3^	3.5037 × 10^3^	3.5012 × 10^3^
F30	Mean	4.9536 × 10^3^	5.0106 × 10^3^	4.9831 × 10^3^	4.9842 × 10^3^	4.9976 × 10^3^	5.0155 × 10^3^	5.0156 × 10^3^
	SD	1.7379 × 10^2^	7.2065 × 10^1^	5.7686 × 10^1^	5.2256 × 10^1^	6.8348 × 10^1^	7.9302 × 10^1^	7.9287 × 10^1^
	Best	4.0584 × 10^3^	4.9426 × 10^3^	4.9430 × 10^3^	4.9426 × 10^3^	4.9433 × 10^3^	4.9430 × 10^3^	4.9434 × 10^3^
	Worst	5.0905 × 10^3^	5.2265 × 10^3^	5.0898 × 10^3^	5.0882 × 10^3^	5.1854 × 10^3^	5.2271 × 10^3^	5.2274 × 10^3^

**Note:** Default denotes the original MSESFOA setting. P-low, Default, and P-high use mutation probability ranges [0.05, 0.25], [0.10, 0.40], and [0.15, 0.55], respectively. F-low, Default, and F-high use F = 0.4 + 0.3r, 0.5 + 0.3r, and 0.6 + 0.3r, respectively, where r is uniformly distributed in [0, 1]. Stage-60, Default, and Stage-80 initiate the late mutation stage at 60%, 70%, and 80% of the maximum iterations, respectively. Only the indicated parameter is changed. SD is the sample standard deviation of 30 runs.

**Table A21:** Rankings of parameter settings within the three sensitivity analysis groups on CEC2017.

Functions	P-Low	Default	P-High	F-Low	Default	F-High	60%	Default	80%
F1	3	2	1	1	2	3	2	1	3
F3	3	2	1	1	2	3	2	3	1
F4	2	1	3	3	1	2	3	1	2
F5	3	1	2	2	1	3	3	1	2
F6	3	1	2	2	1	3	2	1	3
F7	2	1	3	2	1	3	3	1	2
F8	1	2	3	3	2	1	2	3	1
F9	3	1	2	3	1	2	2.5	1	2.5
F10	3	1	2	3	1	2	3	1	2
F11	2	3	1	1	3	2	3	2	1
F12	3	2	1	2	1	3	2	1	3
F13	3	2	1	1	3	2	2	1	3
F14	3	2	1	2	3	1	2	3	1
F15	2	1	3	1	3	2	3	1	2
F16	3	1	2	1	2	3	3	1	2
F17	3	1	2	3	1	2	3	1	2
F18	3	2	1	2	1	3	3	1	2
F19	3	1	2	2	1	3	3	1	2
F20	3	2	1	3	2	1	3	2	1
F21	2	1	3	2	1	3	3	1	2
F22	1	3	2	1.5	3	1.5	1.5	3	1.5
F23	2	1	3	2	1	3	3	1	2
F24	3	1	2	2	1	3	2.5	1	2.5
F25	3	1	2	2	1	3	2.5	1	2.5
F26	1	2	3	1	2	3	3	1	2
F27	1	3	2	2	3	1	2	3	1
F28	3	1	2	3	1	2	2.5	1	2.5
F29	3	2	1	2	1	3	3	1	2
F30	3	1	2	2	1	3	2	1	3
**Mean rank**	2.5172	1.5517	1.9310	1.9828	1.6207	2.3966	2.5690	1.4138	2.0172
**Final rank**	3	1	2	2	1	3	3	1	2

**Note:** Rankings are calculated separately within each parameter group using unrounded mean fitness values. Lower mean fitness values receive better ranks, and exact ties receive average ranks. Mean rank is averaged across the 29 tested functions of CEC2017, with F2 excluded. Final rank is determined by the mean rank.

**Table A22:** Statistical results of the parameter sensitivity analysis on CEC2022.

Functions	Index	Default	P-Low	P-High	F-Low	F-High	Stage-60	Stage-80
F1	Mean	3.0000 × 10^2^	3.0000 × 10^2^	3.0000 × 10^2^	3.0000 × 10^2^	3.0000 × 10^2^	3.0000 × 10^2^	3.0000 × 10^2^
	SD	4.6488 × 10^−4^	1.0165 × 10^−3^	1.0158 × 10^−3^	1.1837 × 10^−4^	2.9848 × 10^−3^	1.0267 × 10^−3^	1.1023 × 10^−3^
	Best	3.0000 × 10^2^	3.0000 × 10^2^	3.0000 × 10^2^	3.0000 × 10^2^	3.0000 × 10^2^	3.0000 × 10^2^	3.0000 × 10^2^
	Worst	3.0000 × 10^2^	3.0000 × 10^2^	3.0000 × 10^2^	3.0000 × 10^2^	3.0001 × 10^2^	3.0000 × 10^2^	3.0000 × 10^2^
F2	Mean	4.3479 × 10^2^	4.4278 × 10^2^	4.4811 × 10^2^	4.4797 × 10^2^	4.4619 × 10^2^	4.4699 × 10^2^	4.4699 × 10^2^
	SD	2.0832 × 10^1^	1.4631 × 10^1^	1.8020 × 10^0^	1.8841 × 10^0^	8.9356 × 10^0^	2.1303 × 10^0^	2.1303 × 10^0^
	Best	4.0000 × 10^2^	4.0000 × 10^2^	4.4490 × 10^2^	4.4490 × 10^2^	4.0001 × 10^2^	4.4490 × 10^2^	4.4490 × 10^2^
	Worst	4.4908 × 10^2^	4.4908 × 10^2^	4.4908 × 10^2^	4.4908 × 10^2^	4.4908 × 10^2^	4.4908 × 10^2^	4.4908 × 10^2^
F3	Mean	6.0000 × 10^2^	6.0000 × 10^2^	6.0000 × 10^2^	6.0000 × 10^2^	6.0000 × 10^2^	6.0000 × 10^2^	6.0000 × 10^2^
	SD	2.2515 × 10^−4^	6.6612 × 10^−4^	1.6659 × 10^−4^	2.2297 × 10^−4^	2.6591 × 10^−4^	3.1250 × 10^−4^	3.3165 × 10^−4^
	Best	6.0000 × 10^2^	6.0000 × 10^2^	6.0000 × 10^2^	6.0000 × 10^2^	6.0000 × 10^2^	6.0000 × 10^2^	6.0000 × 10^2^
	Worst	6.0000 × 10^2^	6.0000 × 10^2^	6.0000 × 10^2^	6.0000 × 10^2^	6.0000 × 10^2^	6.0000 × 10^2^	6.0000 × 10^2^
F4	Mean	8.2800 × 10^2^	8.3651 × 10^2^	8.3038 × 10^2^	8.3439 × 10^2^	8.3570 × 10^2^	8.3396 × 10^2^	8.3231 × 10^2^
	SD	9.4366 × 10^0^	1.2288 × 10^1^	6.5807 × 10^0^	8.3745 × 10^0^	7.5778 × 10^0^	8.2793 × 10^0^	7.1355 × 10^0^
	Best	8.1094 × 10^2^	8.1639 × 10^2^	8.1941 × 10^2^	8.1657 × 10^2^	8.1794 × 10^2^	8.2184 × 10^2^	8.2173 × 10^2^
	Worst	8.4182 × 10^2^	8.6427 × 10^2^	8.4297 × 10^2^	8.5312 × 10^2^	8.5277 × 10^2^	8.5334 × 10^2^	8.4599 × 10^2^
F5	Mean	9.0000 × 10^2^	9.0012 × 10^2^	9.0003 × 10^2^	9.0000 × 10^2^	9.0006 × 10^2^	9.0003 × 10^2^	9.0002 × 10^2^
	SD	2.3313 × 10^−8^	3.4132 × 10^−1^	1.1527 × 10^−1^	6.4793 × 10^−11^	1.3820 × 10^−1^	1.1561 × 10^−1^	8.3971 × 10^−2^
	Best	9.0000 × 10^2^	9.0000 × 10^2^	9.0000 × 10^2^	9.0000 × 10^2^	9.0000 × 10^2^	9.0000 × 10^2^	9.0000 × 10^2^
	Worst	9.0000 × 10^2^	9.0173 × 10^2^	9.0045 × 10^2^	9.0000 × 10^2^	9.0046 × 10^2^	9.0045 × 10^2^	9.0045 × 10^2^
F6	Mean	1.8329 × 10^3^	1.8483 × 10^3^	1.8401 × 10^3^	1.8247 × 10^3^	2.6152 × 10^3^	1.8475 × 10^3^	1.8396 × 10^3^
	SD	1.4444 × 10^1^	2.8105 × 10^1^	2.6031 × 10^1^	1.2803 × 10^1^	4.2121 × 10^3^	1.9427 × 10^1^	1.6480 × 10^1^
	Best	1.8004 × 10^3^	1.8173 × 10^3^	1.8158 × 10^3^	1.8061 × 10^3^	1.8185 × 10^3^	1.8220 × 10^3^	1.8169 × 10^3^
	Worst	1.8700 × 10^3^	1.9185 × 10^3^	1.9624 × 10^3^	1.8695 × 10^3^	2.4916 × 10^4^	1.8983 × 10^3^	1.8806 × 10^3^
F7	Mean	2.0304 × 10^3^	2.0328 × 10^3^	2.0312 × 10^3^	2.0311 × 10^3^	2.0314 × 10^3^	2.0319 × 10^3^	2.0302 × 10^3^
	SD	3.3109 × 10^0^	3.5767 × 10^0^	3.1555 × 10^0^	3.0843 × 10^0^	3.1089 × 10^0^	3.5112 × 10^0^	3.0732 × 10^0^
	Best	2.0217 × 10^3^	2.0256 × 10^3^	2.0243 × 10^3^	2.0252 × 10^3^	2.0257 × 10^3^	2.0252 × 10^3^	2.0252 × 10^3^
	Worst	2.0362 × 10^3^	2.0441 × 10^3^	2.0374 × 10^3^	2.0401 × 10^3^	2.0369 × 10^3^	2.0377 × 10^3^	2.0391 × 10^3^
F8	Mean	2.2229 × 10^3^	2.2239 × 10^3^	2.2235 × 10^3^	2.2237 × 10^3^	2.2236 × 10^3^	2.2239 × 10^3^	2.2234 × 10^3^
	SD	2.1355 × 10^0^	7.5630 × 10^−1^	6.2832 × 10^−1^	6.8626 × 10^−1^	4.9942 × 10^−1^	5.3885 × 10^−1^	6.5413 × 10^−1^
	Best	2.2134 × 10^3^	2.2210 × 10^3^	2.2222 × 10^3^	2.2225 × 10^3^	2.2226 × 10^3^	2.2231 × 10^3^	2.2219 × 10^3^
	Worst	2.2243 × 10^3^	2.2253 × 10^3^	2.2242 × 10^3^	2.2250 × 10^3^	2.2244 × 10^3^	2.2253 × 10^3^	2.2244 × 10^3^
F9	Mean	2.4808 × 10^3^	2.4808 × 10^3^	2.4808 × 10^3^	2.4808 × 10^3^	2.4808 × 10^3^	2.4808 × 10^3^	2.4808 × 10^3^
	SD	0.0000 × 10 ^0^	5.4726 × 10^−13^	5.6647 × 10^−13^	6.0305 × 10^−13^	6.3754 × 10^−13^	5.2736 × 10^−13^	6.0305 × 10^−13^
	Best	2.4808 × 10^3^	2.4808 × 10^3^	2.4808 × 10^3^	2.4808 × 10^3^	2.4808 × 10^3^	2.4808 × 10^3^	2.4808 × 10^3^
	Worst	2.4808 × 10^3^	2.4808 × 10^3^	2.4808 × 10^3^	2.4808 × 10^3^	2.4808 × 10^3^	2.4808 × 10^3^	2.4808 × 10^3^
F10	Mean	2.5005 × 10^3^	2.5005 × 10^3^	2.5005 × 10^3^	2.5005 × 10^3^	2.5005 × 10^3^	2.5005 × 10^3^	2.5005 × 10^3^
	SD	1.0120 × 10^−1^	7.2941 × 10^−2^	6.2170 × 10^−2^	6.6451 × 10^−2^	5.5367 × 10^−2^	5.9357 × 10^−2^	4.7511 × 10^−2^
	Best	2.5003 × 10^3^	2.5004 × 10^3^	2.5004 × 10^3^	2.5004 × 10^3^	2.5005 × 10^3^	2.5004 × 10^3^	2.5004 × 10^3^
	Worst	2.5007 × 10^3^	2.5007 × 10^3^	2.5006 × 10^3^	2.5007 × 10^3^	2.5006 × 10^3^	2.5006 × 10^3^	2.5006 × 10^3^
F11	Mean	2.9100 × 10^3^	2.9300 × 10^3^	2.9600 × 10^3^	2.9533 × 10^3^	2.9500 × 10^3^	2.9400 × 10^3^	2.9400 × 10^3^
	SD	3.0513 × 10^1^	4.6609 × 10^1^	8.1368 × 10^1^	5.0742 × 10^1^	5.0855 × 10^1^	4.9827 × 10^1^	4.9827 × 10^1^
	Best	2.9000 × 10^3^	2.9000 × 10^3^	2.6000 × 10^3^	2.9000 × 10^3^	2.9000 × 10^3^	2.9000 × 10^3^	2.9000 × 10^3^
	Worst	3.0000 × 10^3^	3.0000 × 10^3^	3.0000 × 10^3^	3.0000 × 10^3^	3.0000 × 10^3^	3.0000 × 10^3^	3.0000 × 10^3^
F12	Mean	2.9351 × 10^3^	2.9373 × 10^3^	2.9371 × 10^3^	2.9370 × 10^3^	2.9377 × 10^3^	2.9373 × 10^3^	2.9373 × 10^3^
	SD	7.6122 × 10^0^	3.2706 × 10^0^	3.8933 × 10^0^	4.1118 × 10^0^	4.3443 × 10^0^	4.0151 × 10^0^	3.9638 × 10^0^
	Best	2.9003 × 10^3^	2.9319 × 10^3^	2.9319 × 10^3^	2.9311 × 10^3^	2.9319 × 10^3^	2.9299 × 10^3^	2.9298 × 10^3^
	Worst	2.9439 × 10^3^	2.9439 × 10^3^	2.9460 × 10^3^	2.9454 × 10^3^	2.9477 × 10^3^	2.9459 × 10^3^	2.9459 × 10^3^

**Note:** Default denotes the original MSESFOA setting. P-low, Default, and P-high use mutation probability ranges [0.05, 0.25], [0.10, 0.40], and [0.15, 0.55], respectively. F-low, Default, and F-high use F = 0.4 + 0.3r, 0.5 + 0.3r, and 0.6 + 0.3r, respectively, where r is uniformly distributed in [0, 1]. Stage-60, Default, and Stage-80 initiate the late mutation stage at 60%, 70%, and 80% of the maximum iterations, respectively. Only the indicated parameter is changed. SD is the sample standard deviation of 30 runs.

**Table A23:** Rankings of parameter settings within the three sensitivity analysis groups on CEC2022.

Functions	P-Low	Default	P-High	F-Low	Default	F-High	60%	Default	80%
F1	3	1	2	1	2	3	2	1	3
F2	2	1	3	3	1	2	2.5	1	2.5
F3	3	1	2	2	1	3	2	1	3
F4	3	1	2	2	1	3	3	1	2
F5	3	1	2	1	2	3	3	1	2
F6	3	1	2	1	2	3	3	1	2
F7	3	1	2	2	1	3	3	2	1
F8	3	1	2	3	1	2	3	1	2
F9	2.5	1	2.5	2.5	1	2.5	2.5	1	2.5
F10	3	1	2	2	1	3	3	1	2
F11	2	1	3	3	1	2	3	1	2
F12	3	1	2	2	1	3	2	1	3
**Mean rank**	2.7917	1.0000	2.2083	2.0417	1.2500	2.7083	2.6667	1.0833	2.2500
**Final rank**	3	1	2	2	1	3	3	1	2

**Note:** Rankings are calculated separately within each parameter group using unrounded mean fitness values. Lower mean fitness values receive better ranks, and exact ties receive average ranks. Mean rank is averaged across the 12 CEC2022 functions. Final rank is determined by the mean rank.

## Appendix E. Statistical Uncovered Ratio Results of the 16 Algorithms Based on the 2D WSN Deployment Problem

**Table A24:** Statistical uncovered ratio results of the 16 algorithms based on the 2D WSN deployment problem (first eight algorithms).

Problems	Index	MSESFOA	SFOA	SHO	FLA	TTAO	COA	BKA	IVY
2D-P1	Mean	0.0000 × 10 ^0^	1.2513 × 10^−2^	3.2613 × 10^−2^	7.2000 × 10^−4^	6.1000 × 10^−4^	3.3133 × 10^−3^	3.6667 × 10^−5^	2.4000 × 10^−4^
	SD	0.0000 × 10 ^0^	5.2401 × 10^−3^	7.9911 × 10^−3^	2.1969 × 10^−3^	3.2308 × 10^−4^	3.3047 × 10^−3^	6.6868 × 10^−5^	1.4527 × 10^−4^
	Best	0.0000 × 10 ^0^	6.0000 × 10^−3^	1.9400 × 10^−2^	0.0000 × 10 ^0^	1.0000 × 10^−4^	0.0000 × 10 ^0^	0.0000 × 10 ^0^	0.0000 × 10 ^0^
	Worst	0.0000 × 10 ^0^	2.2600 × 10^−2^	5.0900 × 10^−2^	7.2000 × 10^−3^	1.2000 × 10^−3^	1.1500 × 10^−2^	2.0000 × 10^−4^	5.0000 × 10^−4^
	Rank	1	14	16	10	9	12	3	5
2D-P2	Mean	1.8000 × 10^−4^	2.2080 × 10^−2^	5.5930 × 10^−2^	2.9467 × 10^−3^	3.3800 × 10^−3^	1.0847 × 10^−2^	4.1733 × 10^−3^	4.2333 × 10^−4^
	SD	1.7499 × 10^−4^	1.2113 × 10^−2^	1.0888 × 10^−2^	1.1999 × 10^−2^	9.8730 × 10^−4^	1.0995 × 10^−2^	9.6934 × 10^−3^	4.1745 × 10^−4^
	Best	0.0000 × 10 ^0^	2.1000 × 10^−3^	2.8900 × 10^−2^	0.0000 × 10 ^0^	1.2000 × 10^−3^	2.0000 × 10^−4^	0.0000 × 10 ^0^	0.0000 × 10 ^0^
	Worst	7.0000 × 10^−4^	4.4900 × 10^−2^	7.6600 × 10^−2^	6.5600 × 10^−2^	6.3000 × 10^−3^	3.7500 × 10^−2^	3.8400 × 10^−2^	1.9000 × 10^−3^
	Rank	1	14	16	8	9	12	11	4
2D-P3	Mean	3.8520 × 10^−3^	5.9407 × 10^−2^	9.8370 × 10^−2^	4.9033 × 10^−3^	1.5413 × 10^−2^	3.2587 × 10^−2^	1.6820 × 10^−2^	3.6467 × 10^−3^
	SD	1.9217 × 10^−3^	2.0537 × 10^−2^	1.5580 × 10^−2^	3.9259 × 10^−3^	3.6389 × 10^−3^	2.0366 × 10^−2^	2.3222 × 10^−2^	2.3965 × 10^−3^
	Best	6.0000 × 10^−4^	2.0200 × 10^−2^	6.3500 × 10^−2^	3.0000 × 10^−4^	9.2000 × 10^−3^	6.1000 × 10^−3^	2.6000 × 10^−3^	1.0000 × 10^−3^
	Worst	6.9000 × 10^−3^	9.2100 × 10^−2^	1.2370 × 10^−1^	2.1100 × 10^−2^	2.3300 × 10^−2^	7.9700 × 10^−2^	1.1160 × 10^−1^	1.0000 × 10^−2^
	Rank	4	14	16	6	9	12	10	2
2D-P4	Mean	3.0073 × 10^−2^	1.1240 × 10^−1^	1.8091 × 10^−1^	4.5247 × 10^−2^	5.9783 × 10^−2^	7.5627 × 10^−2^	5.1827 × 10^−2^	2.7961 × 10^−2^
	SD	8.7768 × 10^−3^	2.6328 × 10^−2^	1.5714 × 10^−2^	2.7877 × 10^−3^	8.3553 × 10^−3^	3.2097 × 10^−2^	7.4895 × 10^−3^	5.6497 × 10^−3^
	Best	1.9500 × 10^−2^	7.9300 × 10^−2^	1.5190 × 10^−1^	4.1100 × 10^−2^	4.5200 × 10^−2^	3.7600 × 10^−2^	3.9600 × 10^−2^	1.8520 × 10^−2^
	Worst	4.5200 × 10^−2^	1.4690 × 10^−1^	2.0130 × 10^−1^	4.9100 × 10^−2^	7.2100 × 10^−2^	1.3220 × 10^−1^	6.6400 × 10^−2^	4.2310 × 10^−2^
	Rank	3	14	16	6	11	12	9	2
2D-P5	Mean	3.9633 × 10^−3^	6.1683 × 10^−2^	9.5957 × 10^−2^	8.3067 × 10^−3^	1.8540 × 10^−2^	3.4927 × 10^−2^	1.1520 × 10^−2^	8.0800 × 10^−3^
	SD	2.3032 × 10^−3^	1.5399 × 10^−2^	1.0974 × 10^−2^	4.8249 × 10^−3^	4.1866 × 10^−3^	1.5877 × 10^−2^	1.2241 × 10^−2^	2.1929 × 10^−2^
	Best	3.0000 × 10^−4^	2.9100 × 10^−2^	7.3400 × 10^−2^	1.0000 × 10^−3^	8.6000 × 10^−3^	6.2000 × 10^−3^	2.7000 × 10^−3^	2.0000 × 10^−4^
	Worst	9.1000 × 10^−3^	8.9800 × 10^−2^	1.1250 × 10^−1^	2.0000 × 10^−2^	2.3800 × 10^−2^	6.2300 × 10^−2^	7.2200 × 10^−2^	1.0000 × 10^−1^
	Rank	1	14	16	6	11	12	8	5
2D-P6	Mean	1.6353 × 10^−2^	9.2060 × 10^−2^	1.4264 × 10^−1^	2.2787 × 10^−2^	4.3170 × 10^−2^	6.6653 × 10^−2^	3.1956 × 10^−2^	1.8363 × 10^−2^
	SD	5.1484 × 10^−3^	2.1042 × 10^−2^	1.3742 × 10^−2^	8.2328 × 10^−3^	5.0504 × 10^−3^	1.9338 × 10^−2^	1.4588 × 10^−2^	1.6145 × 10^−2^
	Best	5.5000 × 10^−3^	5.1900 × 10^−2^	1.1750 × 10^−1^	1.1100 × 10^−2^	3.3700 × 10^−2^	3.4600 × 10^−2^	1.1590 × 10^−2^	6.3000 × 10^−3^
	Worst	2.5600 × 10^−2^	1.3850 × 10^−1^	1.6800 × 10^−1^	3.9200 × 10^−2^	5.2400 × 10^−2^	1.0250 × 10^−1^	9.3900 × 10^−2^	7.0000 × 10^−2^
	Rank	1	14	16	6	10	13	9	3
2D-P7	Mean	5.6160 × 10^−2^	1.5920 × 10^−1^	2.1248 × 10^−1^	7.2973 × 10^−2^	9.3693 × 10^−2^	1.0886 × 10^−1^	8.4243 × 10^−2^	5.6417 × 10^−2^
	SD	1.1875 × 10^−2^	2.0870 × 10^−2^	1.7486 × 10^−2^	1.5416 × 10^−2^	9.5683 × 10^−3^	2.4637 × 10^−2^	3.0368 × 10^−2^	3.8064 × 10^−2^
	Best	2.5700 × 10^−2^	1.0030 × 10^−1^	1.7890 × 10^−1^	4.1600 × 10^−2^	6.7000 × 10^−2^	5.9000 × 10^−2^	5.2800 × 10^−2^	2.1900 × 10^−2^
	Worst	7.4000 × 10^−2^	2.0080 × 10^−1^	2.4610 × 10^−1^	1.0110 × 10^−1^	1.1040 × 10^−1^	1.5140 × 10^−1^	1.7580 × 10^−1^	1.8600 × 10^−1^
	Rank	3	14	16	7	10	12	9	5
2D-P8	Mean	1.5026 × 10^−1^	2.5782 × 10^−1^	3.0513 × 10^−1^	1.8696 × 10^−1^	1.9116 × 10^−1^	2.0651 × 10^−1^	1.8431 × 10^−1^	1.5257 × 10^−1^
	SD	1.4038 × 10^−2^	2.5130 × 10^−2^	1.5647 × 10^−2^	5.1365 × 10^−2^	1.0223 × 10^−2^	1.9612 × 10^−2^	3.3667 × 10^−2^	2.2196 × 10^−2^
	Best	1.2220 × 10^−1^	2.1130 × 10^−1^	2.7220 × 10^−1^	1.5330 × 10^−1^	1.7550 × 10^−1^	1.8260 × 10^−1^	1.5210 × 10^−1^	1.2100 × 10^−1^
	Worst	1.6900 × 10^−1^	3.1440 × 10^−1^	3.3580 × 10^−1^	3.3380 × 10^−1^	2.1340 × 10^−1^	2.5760 × 10^−1^	2.9610 × 10^−1^	1.9840 × 10^−1^
	Rank	3	14	16	9	10	12	8	5
Mean rank		2.125	14	16	7.25	9.875	12.125	8.375	3.875
Final rank		1	14	16	7	10	12	9	4

**Table A25:** Statistical uncovered ratio results of the 16 algorithms based on the 2D WSN deployment problem (second eight algorithms).

Problems	Index	NRBO	PKO	EAO	ALA	RIME	IRIME	SAO	ISAO
2D-P1	Mean	1.9603 × 10^−2^	3.1667 × 10^−4^	8.7333 × 10^−3^	4.9333 × 10^−4^	1.0000 × 10^−5^	7.2667 × 10^−4^	1.4333 × 10^−4^	3.9333 × 10^−4^
	SD	8.1496 × 10^−3^	4.6467 × 10^−4^	9.9416 × 10^−3^	9.8888 × 10^−4^	3.0513 × 10^−5^	2.1948 × 10^−3^	1.6543 × 10^−4^	4.6530 × 10^−4^
	Best	8.0000 × 10^−3^	0.0000 × 10 ^0^	0.0000 × 10 ^0^	0.0000 × 10 ^0^	0.0000 × 10 ^0^	0.0000 × 10 ^0^	0.0000 × 10 ^0^	0.0000 × 10 ^0^
	Worst	3.4500 × 10^−2^	2.3000 × 10^−3^	2.5700 × 10^−2^	3.1000 × 10^−3^	1.0000 × 10^−4^	7.2000 × 10^−3^	5.0000 × 10^−4^	1.5000 × 10^−3^
	Rank	15	6	13	8	2	11	4	7
2D-P2	Mean	3.9200 × 10^−2^	1.4233 × 10^−3^	1.7717 × 10^−2^	3.6433 × 10^−3^	2.3667 × 10^−4^	5.5000 × 10^−4^	4.0000 × 10^−4^	1.3600 × 10^−3^
	SD	1.2179 × 10^−2^	1.2670 × 10^−3^	1.7293 × 10^−2^	3.8045 × 10^−3^	3.1892 × 10^−4^	8.0247 × 10^−4^	4.1689 × 10^−4^	1.2486 × 10^−3^
	Best	1.9300 × 10^−2^	1.0000 × 10^−4^	6.0000 × 10^−4^	0.0000 × 10 ^0^	0.0000 × 10 ^0^	0.0000 × 10 ^0^	0.0000 × 10 ^0^	0.0000 × 10 ^0^
	Worst	7.1100 × 10^−2^	4.3000 × 10^−3^	5.3600 × 10^−2^	1.1900 × 10^−2^	1.4000 × 10^−3^	2.7000 × 10^−3^	1.3000 × 10^−3^	6.0000 × 10^−3^
	Rank	15	7	13	10	2	5	3	6
2D-P3	Mean	8.4963 × 10^−2^	7.7133 × 10^−3^	3.3307 × 10^−2^	1.8900 × 10^−2^	3.7233 × 10^−3^	4.2900 × 10^−3^	3.6067 × 10^−3^	5.1733 × 10^−3^
	SD	1.5648 × 10^−2^	4.2653 × 10^−3^	2.2378 × 10^−2^	8.6807 × 10^−3^	2.8181 × 10^−3^	2.8700 × 10^−3^	3.5637 × 10^−3^	1.8021 × 10^−3^
	Best	5.2200 × 10^−2^	1.8000 × 10^−3^	9.1000 × 10^−3^	7.1000 × 10^−3^	5.0000 × 10^−4^	3.0000 × 10^−4^	3.0000 × 10^−4^	2.6000 × 10^−3^
	Worst	1.1190 × 10^−1^	1.9000 × 10^−2^	9.3900 × 10^−2^	4.2700 × 10^−2^	1.4400 × 10^−2^	1.1000 × 10^−2^	1.5000 × 10^−2^	9.1000 × 10^−3^
	Rank	15	8	13	11	3	5	1	7
2D-P4	Mean	1.5023 × 10^−1^	4.7660 × 10^−2^	7.7163 × 10^−2^	4.6543 × 10^−2^	3.4010 × 10^−2^	3.6190 × 10^−2^	5.9530 × 10^−2^	2.5140 × 10^−2^
	SD	1.9441 × 10^−2^	7.9086 × 10^−3^	3.8068 × 10^−2^	1.2863 × 10^−2^	6.7234 × 10^−3^	9.3062 × 10^−3^	6.1439 × 10^−2^	4.7219 × 10^−3^
	Best	1.1690 × 10^−1^	4.0800 × 10^−2^	4.8600 × 10^−2^	3.9800 × 10^−2^	2.3200 × 10^−2^	1.9400 × 10^−2^	2.2900 × 10^−2^	2.0000 × 10^−2^
	Worst	1.7400 × 10^−1^	6.0800 × 10^−2^	1.7050 × 10^−1^	7.8500 × 10^−2^	4.6600 × 10^−2^	4.3900 × 10^−2^	1.8400 × 10^−1^	3.2100 × 10^−2^
	Rank	15	8	13	7	4	5	10	1
2D-P5	Mean	7.9230 × 10^−2^	1.0623 × 10^−2^	4.4813 × 10^−2^	1.8377 × 10^−2^	4.2143 × 10^−3^	4.2900 × 10^−3^	7.5533 × 10^−3^	1.3517 × 10^−2^
	SD	1.6730 × 10^−2^	6.2371 × 10^−3^	2.7945 × 10^−2^	9.2390 × 10^−3^	3.2667 × 10^−3^	2.7870 × 10^−3^	3.0959 × 10^−3^	5.4235 × 10^−3^
	Best	4.7200 × 10^−2^	7.0000 × 10^−4^	1.0400 × 10^−2^	2.1000 × 10^−3^	1.0000 × 10^−3^	1.0000 × 10^−3^	2.8000 × 10^−3^	4.7000 × 10^−3^
	Worst	1.2960 × 10^−1^	2.8900 × 10^−2^	9.5500 × 10^−2^	3.9700 × 10^−2^	1.5000 × 10^−2^	1.3600 × 10^−2^	1.4900 × 10^−2^	3.1700 × 10^−2^
	Rank	15	7	13	10	2	3	4	9
2D-P6	Mean	1.2616 × 10^−1^	2.3980 × 10^−2^	5.9123 × 10^−2^	4.8533 × 10^−2^	1.8437 × 10^−2^	2.1567 × 10^−2^	1.8220 × 10^−2^	2.7307 × 10^−2^
	SD	1.7136 × 10^−2^	1.2240 × 10^−2^	3.0844 × 10^−2^	1.6497 × 10^−2^	1.5945 × 10^−2^	1.7789 × 10^−2^	4.5280 × 10^−3^	8.1800 × 10^−3^
	Best	9.8600 × 10^−2^	6.5000 × 10^−3^	2.0100 × 10^−2^	2.3500 × 10^−2^	4.4000 × 10^−3^	8.8000 × 10^−3^	1.0000 × 10^−2^	1.2000 × 10^−2^
	Worst	1.6180 × 10^−1^	5.6900 × 10^−2^	1.3630 × 10^−1^	9.5000 × 10^−2^	8.9000 × 10^−2^	9.6000 × 10^−2^	2.6800 × 10^−2^	4.8500 × 10^−2^
	Rank	15	7	12	11	4	5	2	8
2D-P7	Mean	1.9446 × 10^−1^	7.4133 × 10^−2^	1.1632 × 10^−1^	9.4417 × 10^−2^	5.0007 × 10^−2^	5.6320 × 10^−2^	4.5423 × 10^−2^	5.7160 × 10^−2^
	SD	1.3793 × 10^−2^	1.5346 × 10^−2^	3.4346 × 10^−2^	1.6900 × 10^−2^	8.8190 × 10^−3^	7.7826 × 10^−3^	9.6975 × 10^−3^	7.8569 × 10^−3^
	Best	1.6490 × 10^−1^	4.7800 × 10^−2^	7.2900 × 10^−2^	6.3800 × 10^−2^	3.4000 × 10^−2^	3.7900 × 10^−2^	3.1500 × 10^−2^	4.3900 × 10^−2^
	Worst	2.1760 × 10^−1^	1.0260 × 10^−1^	1.9490 × 10^−1^	1.3940 × 10^−1^	6.7800 × 10^−2^	6.7800 × 10^−2^	6.9500 × 10^−2^	7.3400 × 10^−2^
	Rank	15	8	13	11	2	4	1	6
2D-P8	Mean	2.9606 × 10^−1^	1.6705 × 10^−1^	2.1690 × 10^−1^	1.9765 × 10^−1^	1.5196 × 10^−1^	1.5761 × 10^−1^	1.4522 × 10^−1^	1.4169 × 10^−1^
	SD	1.5343 × 10^−2^	1.8146 × 10^−2^	4.1279 × 10^−2^	2.1385 × 10^−2^	8.8947 × 10^−3^	1.0271 × 10^−2^	3.2721 × 10^−2^	6.8763 × 10^−3^
	Best	2.7290 × 10^−1^	1.2560 × 10^−1^	1.7840 × 10^−1^	1.6500 × 10^−1^	1.3810 × 10^−1^	1.3860 × 10^−1^	1.2290 × 10^−1^	1.2940 × 10^−1^
	Worst	3.4020 × 10^−1^	2.0080 × 10^−1^	3.1240 × 10^−1^	2.4250 × 10^−1^	1.7250 × 10^−1^	1.7830 × 10^−1^	3.0560 × 10^−1^	1.5680 × 10^−1^
	Rank	15	7	13	11	4	6	2	1
Mean rank		15	7.25	12.875	9.875	2.875	5.5	3.375	5.625
Final rank		15	7	13	10	2	5	3	6

## Appendix F. Statistical Uncovered Ratio Results of the 16 Algorithms Based on the 3D WSN Deployment Problem

**Table A26:** Statistical uncovered ratio results of the 16 algorithms based on the 3D WSN deployment problem (first eight algorithms).

Problems	Index	MSESFOA	SFOA	SHO	FLA	TTAO	COA	BKA	IVY
3D-P1	Mean	2.5333 × 10^−5^	1.0718 × 10^−2^	2.1340 × 10^−2^	4.3333 × 10^−5^	4.6000 × 10^−4^	3.5933 × 10^−3^	1.2667 × 10^−4^	1.3500 × 10^−4^
	SD	3.6363 × 10^−5^	4.6443 × 10^−3^	3.8127 × 10^−3^	8.2768 × 10^−5^	2.3723 × 10^−4^	4.5478 × 10^−3^	1.2713 × 10^−4^	6.1798 × 10^−5^
	Best	0.0000 × 10 ^0^	3.9000 × 10^−3^	1.2950 × 10^−2^	0.0000 × 10 ^0^	1.5000 × 10^−4^	5.0000 × 10^−5^	0.0000 × 10 ^0^	0.0000 × 10 ^0^
	Worst	1.0000 × 10^−4^	2.2550 × 10^−2^	2.6900 × 10^−2^	3.0000 × 10^−4^	1.1500 × 10^−3^	1.5700 × 10^−2^	5.5000 × 10^−4^	3.5000 × 10^−4^
	Rank	1	15	16	4	10	13	7	8
3D-P2	Mean	2.4467 × 10^−4^	1.8050 × 10^−2^	3.7253 × 10^−2^	4.3667 × 10^−4^	2.2550 × 10^−3^	7.2783 × 10^−3^	1.1268 × 10^−2^	2.7833 × 10^−4^
	SD	2.1229 × 10^−4^	6.8680 × 10^−3^	5.9650 × 10^−3^	5.7490 × 10^−4^	7.0348 × 10^−4^	7.2170 × 10^−3^	1.3742 × 10^−2^	2.0371 × 10^−4^
	Best	0.0000 × 10 ^0^	4.8000 × 10^−3^	2.6000 × 10^−2^	0.0000 × 10 ^0^	9.0000 × 10^−4^	1.2500 × 10^−3^	2.0000 × 10^−4^	5.0000 × 10^−5^
	Worst	7.5000 × 10^−4^	3.4350 × 10^−2^	4.9550 × 10^−2^	2.5000 × 10^−3^	3.0500 × 10^−3^	2.9850 × 10^−2^	3.0750 × 10^−2^	1.0000 × 10^−3^
	Rank	1	14	16	5	10	12	13	3
3D-P3	Mean	3.1647 × 10^−3^	3.5020 × 10^−2^	7.1933 × 10^−2^	4.5217 × 10^−3^	8.4650 × 10^−3^	1.4618 × 10^−2^	1.6430 × 10^−2^	4.4000 × 10^−3^
	SD	1.4869 × 10^−3^	1.1773 × 10^−2^	9.1764 × 10^−3^	2.3355 × 10^−3^	1.1365 × 10^−3^	9.1607 × 10^−3^	1.9848 × 10^−2^	4.0454 × 10^−3^
	Best	5.7000 × 10^−4^	1.7550 × 10^−2^	5.5700 × 10^−2^	1.6000 × 10^−3^	6.8500 × 10^−3^	3.1500 × 10^−3^	2.5500 × 10^−3^	3.0000 × 10^−4^
	Worst	5.2500 × 10^−3^	6.0350 × 10^−2^	8.8750 × 10^−2^	9.7500 × 10^−3^	1.1150 × 10^−2^	4.0300 × 10^−2^	6.4600 × 10^−2^	1.2500 × 10^−2^
	Rank	2	14	16	7	9	12	13	6
3D-P4	Mean	1.7192 × 10^−2^	6.8877 × 10^−2^	1.3261 × 10^−1^	2.6117 × 10^−2^	2.8470 × 10^−2^	4.3637 × 10^−2^	2.7613 × 10^−2^	2.3175 × 10^−2^
	SD	7.7522 × 10^−3^	2.1484 × 10^−2^	1.8209 × 10^−2^	6.7775 × 10^−3^	5.0949 × 10^−3^	1.4947 × 10^−2^	2.5737 × 10^−2^	2.4943 × 10^−2^
	Best	1.9400 × 10^−3^	2.6550 × 10^−2^	7.7850 × 10^−2^	1.2750 × 10^−2^	8.5500 × 10^−3^	1.0350 × 10^−2^	4.0500 × 10^−3^	4.9500 × 10^−3^
	Worst	3.5400 × 10^−2^	1.1005 × 10^−1^	1.6155 × 10^−1^	3.9450 × 10^−2^	3.7150 × 10^−2^	7.8600 × 10^−2^	1.3745 × 10^−1^	9.6000 × 10^−2^
	Rank	2	14	16	8	10	13	9	7
Mean rank		1.5	14.25	16	6	9.75	12.5	10.5	6
Final rank		1	14	16	6	9	13	11	6

**Table A27:** Statistical uncovered ratio results of the 16 algorithms based on the 3D WSN deployment problem (second set of eight algorithms).

Problems	Index	NRBO	PKO	EAO	ALA	RIME	IRIME	SAO	ISAO
3D-P1	Mean	9.2033 × 10^−3^	1.1783 × 10^−3^	2.9500 × 10^−4^	8.7000 × 10^−4^	7.8333 × 10^−5^	6.1667 × 10^−5^	3.5000 × 10^−5^	3.6667 × 10^−5^
	SD	3.6128 × 10^−3^	8.3229 × 10^−4^	3.6255 × 10^−4^	6.0395 × 10^−4^	1.1347 × 10^−4^	9.0671 × 10^−5^	8.0032 × 10^−5^	6.8145 × 10^−5^
	Best	5.0500 × 10^−3^	1.5000 × 10^−4^	5.0000 × 10^−5^	0.0000 × 10 ^0^	0.0000 × 10 ^0^	0.0000 × 10 ^0^	0.0000 × 10 ^0^	0.0000 × 10 ^0^
	Worst	1.4900 × 10^−2^	2.6000 × 10^−3^	1.6500 × 10^−3^	2.8500 × 10^−3^	4.0000 × 10^−4^	3.0000 × 10^−4^	3.0000 × 10^−4^	3.0000 × 10^−4^
	Rank	14	12	9	11	6	5	2	3
3D-P2	Mean	3.0043 × 10^−2^	2.2017 × 10^−3^	2.0050 × 10^−3^	2.9417 × 10^−3^	5.5333 × 10^−4^	8.0333 × 10^−4^	2.8167 × 10^−4^	2.5667 × 10^−4^
	SD	8.3623 × 10^−3^	1.1319 × 10^−3^	6.6958 × 10^−4^	1.3978 × 10^−3^	4.0192 × 10^−4^	5.8838 × 10^−4^	2.3653 × 10^−4^	2.0542 × 10^−4^
	Best	1.6800 × 10^−2^	1.2000 × 10^−3^	5.5000 × 10^−4^	6.0000 × 10^−4^	0.0000 × 10 ^0^	1.0000 × 10^−4^	0.0000 × 10 ^0^	0.0000 × 10 ^0^
	Worst	4.7850 × 10^−2^	5.7500 × 10^−3^	3.3500 × 10^−3^	5.0500 × 10^−3^	1.3000 × 10^−3^	3.1000 × 10^−3^	1.0000 × 10^−3^	9.0000 × 10^−4^
	Rank	15	9	8	11	6	7	4	2
3D-P3	Mean	5.6775 × 10^−2^	6.1333 × 10^−3^	1.0180 × 10^−2^	1.0565 × 10^−2^	3.7193 × 10^−3^	3.5650 × 10^−3^	2.9083 × 10^−3^	3.2400 × 10^−3^
	SD	8.5074 × 10^−3^	3.2211 × 10^−3^	3.0154 × 10^−3^	6.4573 × 10^−3^	2.4136 × 10^−3^	1.7082 × 10^−3^	3.6318 × 10^−3^	2.7056 × 10^−3^
	Best	3.8800 × 10^−2^	2.3000 × 10^−3^	6.1000 × 10^−3^	3.6000 × 10^−3^	1.3000 × 10^−3^	1.8000 × 10^−3^	4.5000 × 10^−4^	1.3000 × 10^−3^
	Worst	6.6700 × 10^−2^	1.4900 × 10^−2^	1.6450 × 10^−2^	2.5700 × 10^−2^	1.0000 × 10^−2^	9.3000 × 10^−3^	1.1500 × 10^−2^	9.5000 × 10^−3^
	Rank	15	8	10	11	5	4	1	3
3D-P4	Mean	1.2212 × 10^−1^	2.2768 × 10^−2^	4.3535 × 10^−2^	4.0413 × 10^−2^	2.0227 × 10^−2^	2.0723 × 10^−2^	1.6943 × 10^−2^	1.9212 × 10^−2^
	SD	1.8287 × 10^−2^	8.1682 × 10^−3^	1.0910 × 10^−2^	1.2045 × 10^−2^	6.6108 × 10^−3^	6.8721 × 10^−3^	1.1469 × 10^−2^	2.2516 × 10^−2^
	Best	6.6700 × 10^−2^	1.0300 × 10^−2^	2.8750 × 10^−2^	2.2950 × 10^−2^	1.1050 × 10^−2^	1.0100 × 10^−2^	8.9500 × 10^−3^	7.8500 × 10^−3^
	Worst	1.5105 × 10^−1^	4.4750 × 10^−2^	8.5500 × 10^−2^	8.7000 × 10^−2^	3.5450 × 10^−2^	4.3000 × 10^−2^	5.0000 × 10^−2^	8.6000 × 10^−2^
	Rank	15	6	12	11	4	5	1	3
Mean rank		14.75	8.75	9.75	11	5.25	5.25	2	2.75
Final rank		15	8	9	12	4	4	2	3
